# A difference ring theory for symbolic summation^☆^

**DOI:** 10.1016/j.jsc.2015.02.002

**Published:** 2016

**Authors:** Carsten Schneider

**Affiliations:** Research Institute for Symbolic Computation (RISC), Johannes Kepler University Linz, Altenbergerstraße 69, 4040 Linz, Austria

**Keywords:** Difference ring extensions, Roots of unity, Indefinite nested sums and products, Parameterized telescoping (telescoping creative telescoping), Semi-constants, Semi-invariants

## Abstract

A summation framework is developed that enhances Karr's difference field approach. It covers not only indefinite nested sums and products in terms of transcendental extensions, but it can treat, e.g., nested products defined over roots of unity. The theory of the so-called $R\Pi \Sigma^{⁎}$-extensions is supplemented by algorithms that support the construction of such difference rings automatically and that assist in the task to tackle symbolic summation problems. Algorithms are presented that solve parameterized telescoping equations, and more generally parameterized first-order difference equations, in the given difference ring. As a consequence, one obtains algorithms for the summation paradigms of telescoping and Zeilberger's creative telescoping. With this difference ring theory one gets a rigorous summation machinery that has been applied to numerous challenging problems coming, e.g., from combinatorics and particle physics.

## 1 Introduction

In his pioneering work M. Karr (1981, 1985) introduced a very general class of difference fields, the so-called ΠΣ-fields, in which expressions in terms of indefinite nested sums and products can be represented. In particular, he developed an algorithm that decides constructively if for a given expression f(k) represented in a ΠΣ-field F there is an expression g(k) represented in the field F such that the telescoping equation (anti-difference)

(1) f(k)=g(k+1)−g(k)

holds. If such a solution exists, one obtains for an appropriately chosen a∈N the identity

(2) $$\sum_{k=a}^{b}f(k)=g(b+1)-g(a).$$

His algorithms can be viewed as the discrete version of Risch's integration algorithm; see Risch (1969), Bronstein (1997). In the last years the ΠΣ-field theory has been pushed forward. It is now possible to obtain sum representations, i.e., right hand sides in (2) with certain optimality criteria such as minimal nesting depth (Schneider, 2008, 2010c), minimal number of generators in the summands (Schneider, 2004c) or minimal degrees in the denominators (Schneider, 2007b). For the simplification of products see Schneider (2005c), Abramov and Petkovšek (2010). We emphasize that exactly such refined representations give rise to more efficient telescoping algorithms worked out in Schneider (2010b, 2015).

A striking application is that Karr's algorithm and all the enhanced versions can be used to solve the parameterized telescoping problem (Schneider, 2000, 2010a): for given indefinite nested product-sum expressions $f_{1}(k),\ldots ,f_{n}(k)$ represented in F, find constants $c_{1},\ldots ,c_{n}$, free of *k* and not all zero, and find g(k) represented in F such that

(3) $$g(k+1)-g(k)=c_{1}\;f_{1}(k)+\cdots +c_{n}\;f_{n}(k)$$

holds. In particular, this problem covers Zeilberger's creative telescoping paradigm (Zeilberger, 1991) for a bivariate function F(m,k) by setting $f_{i}(k)=F(m+i-1,k)$ with i∈{1,…,n} and representing these $f_{i}(k)$ in F. Namely, if one finds such a solution, one ends up at the recurrence

$$g(m,b+1)-g(m,a)=c_{1}\;\sum_{k=a}^{b}f(m,k)+\cdots +c_{n}\;\sum_{k=a}^{b}f(m+n-1,k).$$

In a nutshell, one cannot only treat indefinite summation but also definite summation problems. In this regard, also recurrence solvers have been developed where the coefficients of the recurrence and the inhomogeneous part can be elements from a ΠΣ-field (Bronstein, 2000; Schneider, 2005d; Abramov et al., in preparation). All these algorithms generalize and enhance substantially the (*q*-)hypergeometric and holonomic toolbox (Abramov, 1971; Gosper, 1978; Zeilberger, 1990, 1991; Petkovšek, 1992; Paule, 1995; Petkovšek et al., 1996; Paule and Riese, 1997; Bauer and Petkovšek, 1999; Chyzak, 2000; Kauers and Paule, 2011; Koutschan, 2013) in order to rewrite definite sums to indefinite nested sums. For details on these aspects we refer to Schneider (2014).

Besides all these sophisticated developments, e.g., within the summation package Sigma (Schneider, 2007c), there is one critical gap which concerns all the developed tools in the setting of difference fields: Algebraic products, like

(4) $${\left(-1\right)}^{k}=\prod_{i=1}^{k}(-1),\;{\left(-1\right)}^{\left(\begin{array}{c} k+1\\ 2 \end{array}\right)}=\prod_{i=1}^{k}\prod_{j=1}^{i}(-1),\;{\left(-1\right)}^{\left(\begin{array}{c} k+2\\ 3 \end{array}\right)}=\prod_{i=1}^{k}\prod_{j=1}^{i}\prod_{k=1}^{j}(-1),\ldots$$

cannot be expressed in ΠΣ-fields, which are built by a tower of transcendental field extensions. Even worse, the objects given in (4) introduce zero-divisors, like

(5) $$(1-{\left(-1\right)}^{k})(1+{\left(-1\right)}^{k})=0$$

which cannot be treated in a field or in an integral domain. In applications these objects occur rather frequently as standalone objects or in nested sums (Ablinger et al., 2011, 2013). It is thus a fundamental challenge to include such objects in an enhanced summation theory.

With the elegant theory of van der Put and Singer (1997), Hardouin and Singer (2008) one can handle such objects by several copies of the underlying difference field, i.e., by implementing the concept of interlacing in an algebraic way. First steps to combine these techniques with ΠΣ-fields have been made in Eröcal (2011).

Within the package Sigma a different approach (Schneider, 2001) has been implemented. Summation objects like ${\left(-1\right)}^{k}$ and sums over such objects are introduced by a tower of generators subject to the relations such as (5). In this way one obtains a direct translation between the summation objects and the generators of the corresponding difference rings. This enhancement has been applied non-trivially, e.g., to combinatorial problems (Schneider, 2004b; Prodinger et al., 2011), number theory (Schneider, 2007a; Osburn and Schneider, 2009) or to problems from particle physics (Blümlein et al., 2012); for the most recent evaluations of Feynman integrals (Blümlein et al., 2013; Ablinger et al., 2014a, 2014b) up to 300 generators were used to model the summation objects in difference rings. But so far, this successful and very efficient machinery of Sigma was built, at least partially, on heuristic considerations.

In this article we shall develop the underlying difference ring theory and supplement it with the missing algorithmic building blocks in order to obtain a rigorous summation machinery. More precisely, we will enhance the difference field theory of Karr (1981, 1985) to a difference ring theory by introducing besides Π-extensions (for transcendental product extensions) and $\Sigma^{⁎}$-extensions (for transcendental sum extensions) also *R*-extensions which enables one to represent objects such as (4). An important ingredient of this theory is the exploration of the so-called semi-constants (resp. semi-invariants) and the formulation of the symbolic summation problems within these notions. In particular, we obtain algorithms that can solve certain classes of parameterized first-order linear difference equations. As special instances we obtain algorithms for the parameterized telescoping problem, in particular for the summation paradigms of telescoping and creative telescoping. In addition, we provide an algorithmic toolbox that supports the construction of the so-called simple $R\Pi \Sigma^{⁎}$-extensions automatically. As a special case we demonstrate, how d'Alembertian solutions (Abramov and Petkovšek, 1994) of a recurrence, a subclass of Liouvillian solutions (Hendriks and Singer, 1999; Petkovšek and Zakrajšek, 2013), can be represented in such $R\Pi \Sigma^{⁎}$-extensions. In particular, we will illustrate the underlying problems and their solutions by discovering the following identities

(6) $$\sum_{k=1}^{b}{\left(-1\right)}^{\left(\begin{array}{c} k+1\\ 2 \end{array}\right)}k^{2}\sum_{j=1}^{k}\frac{{\left(-1\right)}^{j}}{j}=\frac{1}{2}\sum_{j=1}^{b}\frac{{\left(-1\right)}^{\left(\begin{array}{c} j+1\\ 2 \end{array}\right)}}{j}-\frac{1}{4}{\left(-1\right)}^{\left(\begin{array}{c} b+1\\ 2 \end{array}\right)}(-1+{\left(-1\right)}^{b}+2b)+{\left(-1\right)}^{\left(\begin{array}{c} b+1\\ 2 \end{array}\right)}\frac{1}{2}(b(b+2)+{\left(-1\right)}^{b}(b^{2}-1))\sum_{j=1}^{b}\frac{{\left(-1\right)}^{j}}{j},$$

(7) $$\prod_{k=1}^{b}\frac{-(\iota^{k})}{1+k}=(-\frac{\iota }{2}-\frac{1}{2})\frac{-{\left(-1\right)}^{b}+\iota }{b(b+1)}(\prod_{j=1}^{b-1}\frac{\iota^{j}}{j});$$

here the imaginary unit is denoted by *ι*, i.e., $\iota^{2}=-1$.

The outline is as follows. In Section 2 we will introduce the basic notations of difference rings (resp. fields) and define $R\Pi \Sigma^{⁎}$-ring extensions. Furthermore, we will work out the underlying problems in the setting of difference rings and motivate the different challenges that will be treated in this article. In addition, we give an overview of the main results and show how they can be applied for symbolic summation. In the remaining sections these results will be worked out in detail. In Section 3 we present the crucial properties of single nested $R\Pi \Sigma^{⁎}$-extensions. Special emphasis will be put on the properties of the underlying ring. In Section 4 we will consider a tower of such extensions and explore the set of semi-constants. In Section 5 we present algorithms that calculate the order, period and factorial order of the generators of *R*-extensions. Finally, in Section 6 and Section 7 we elaborate algorithms that are needed to construct $R\Pi \Sigma^{⁎}$-extensions and that solve as a special case the (parameterized) telescoping problem. A conclusion is given in Section 8.

## 2 Basic definitions, the outline of the problems, and the main results

In this article all rings are commutative with 1 and all rings (resp. fields) have characteristic 0; in particular, they contain the rational numbers Q as a subring (resp. subfield). A ring (resp. field) is called computable if there are algorithms available that can perform the standard operations (including zero recognition and deciding constructively if an element is invertible). The multiplicative group of units (invertible elements) of a ring A is denoted by $A^{⁎}$. The ideal generated by S⊆A is denoted by 〈S〉. If A is a subring (resp. subfield/multiplicative subgroup) of $\overset{˜}{A}$ we also write $A\leq \overset{˜}{A}$. The non-negative integers are denoted by N={0,1,2,…}.

In this section we will present a general framework in which our symbolic summation problems can be formulated and tackled in the setting of difference rings. Here an indefinite nested product-sum expression f(k) (like in (1) or (3)) is described in a ring (resp. field) A and the shift behavior of such an expression is reflected by a ring automorphism (resp. field automorphism) σ:A→A, i.e., $\sigma^{i}(f)$ with i∈Z represents the expression f(k+i). In the following we call such a ring A (resp. field) equipped with a ring automorphism (resp. field automorphism) *σ* a difference ring (resp. difference field) (Cohn, 1965; Levin, 2008) and denote it by (A,σ). We remark that any difference field is also a difference ring. Conversely, any difference ring (A,σ) with A being a field is automatically a difference field. A difference ring (resp. field) (A,σ) is called computable if both, A and the function *σ* are computable; note that in such rings one can decide if an element is a constant, i.e., if σ(c)=c. The set of constants is also denoted by const(A,σ)={c∈A|σ(c)=c}, and if it is clear from the context, we also write constA=const(A,σ). It is easy to check that constA is a subring (resp. a subfield) of A which contains as subring (resp. subfield) the rational numbers Q. Throughout this article we will take care that constA is always a field (and not just a ring), called the constant field and denoted by K.

In the first subsection we introduce the class of difference rings in which we will model indefinite nested sums and products. They will be introduced by a tower of ring extensions, the so-called $R\Pi \Sigma^{⁎}$-ring extensions.

In Subsection 2.2 we will focus on two tasks:

(1) Introduce techniques that enable one to test if the given tower of extensions is an $R\Pi \Sigma^{⁎}$-extension; even more, derive tactics that enable one to represent sums and products automatically in $R\Pi \Sigma^{⁎}$-extensions.
(2) Work out the underlying subproblems in order to solve two central problems of symbolic summation: telescoping (compare (1)) and parameterized telescoping (compare (3)). In their simplest form they can be specified as follows.

**Problem T for** (A,σ)**.** *Given* a difference ring (A,σ) and given f∈A. *Find*, if possible, a g∈A such that the telescoping (T) equation holds:

(8) σ(g)−g=f.

**Problem PT for** (A,σ)**.** *Given* a difference ring (A,σ) with constant field K and given $f_{1},\ldots ,f_{n}\in A$. *Find*, if possible, $c_{1},\ldots ,c_{n}\in K$ (not all $c_{i}$ being zero) and a g∈A such that the parameterized telescoping (PT) holds:

(9) $$\sigma (g)-g=c_{1}\;f_{1}+\cdots +c_{n}\;f_{n}.$$

In Subsection 2.3 we will present the main results of theoretical and algorithmic nature to handle these problems, and in Subsection 2.4 we demonstrate how the new summation theory can be used to represent d'Alembertian solutions in $R\Pi \Sigma^{⁎}$-extensions.

### 2.1 The definition of $R\Pi \Sigma^{⁎}$-extensions

A difference ring $\left(\overset{˜}{A},\overset{˜}{\sigma }\right)$ is a difference ring extension of a difference ring (A,σ) if $A\leq \overset{˜}{A}$ and $\overset{˜}{\sigma }{|}_{A}=\sigma$, i.e., A is a subring of $\overset{˜}{A}$ and $\overset{˜}{\sigma }(a)=\sigma (a)$ for all a∈A. The definition of difference field extensions is the same by replacing the word ring with field. In short (for the ring and field version) we also write $\left(A,\sigma )\leq (\overset{˜}{A},\overset{˜}{\sigma }\right)$. If it is clear from the context, we do not distinguish anymore between *σ* and $\overset{˜}{\sigma }$.

For the construction of $R\Pi \Sigma^{⁎}$-extensions, we start with the following basic properties.

Lemma 2.1 *Let* A *be a ring with* $\alpha \in A^{⁎}$ *and* β∈A *equipped with a ring automorphism* σ:A→A*. Let* A[t] *be a polynomial ring and* $A[t,\frac{1}{t}]$ *be a ring of Laurent polynomials.*

(1) *There is a unique automorphism* $\sigma^{\prime }:A[t]\to A[t]$ *with* $\sigma^{\prime }{|}_{A}=\sigma$ *and* $\sigma^{\prime }(t)=\alpha \;t+\beta$*.*
(2) *There is a unique automorphism* $\sigma^{\prime \prime }:A[t,\frac{1}{t}]\to A[t,\frac{1}{t}]$ *with* $\sigma^{\prime \prime }{|}_{A}=\sigma$ *and* $\sigma^{\prime \prime }(t)=\alpha \;t$ *(where* $\sigma^{\prime \prime }(\frac{1}{t})=\alpha^{-1}\;\frac{1}{t}$*). In particular, if* β=0*,* $\sigma^{\prime \prime }{|}_{A[t]}=\sigma^{\prime }$*.*
(3) *If* A *is field and* A(t) *is a rational function field, there is a unique field automorphism* $\sigma^{\prime \prime \prime }:A(t)\to A(t)$ *with* $\sigma^{\prime \prime \prime }{|}_{A}=\sigma$ *and* $\sigma^{\prime \prime \prime }(t)=\alpha \;t+\beta$*. In particular,* $\sigma^{\prime \prime \prime }{|}_{A[t]}=\sigma^{\prime }$*; moreover,* $\sigma^{\prime \prime \prime }{|}_{A[t,1/t]}=\sigma^{\prime \prime }$ *if* β=0*.*

In summary, let (A,σ) be a difference ring and *t* be transcendental over A. Then we obtain the uniquely determined difference ring extension (A[t],σ) of (A,σ) with σ(t)=αt+β where $\alpha \in A^{⁎}$ and β∈A. In particular, we get the uniquely determined difference ring extension $\left(A[t,\frac{1}{t}],\sigma \right)$ of (A,σ) with σ(t)=αt. Thus for β=0, we have the chain of extensions $\left(A,\sigma )\leq (A[t],\sigma )\leq (A[t,\frac{1}{t}],\sigma \right)$. Moreover, if A is a field, we obtain the uniquely determined difference field extension (A(t),σ) of (A,σ) with σ(t)=αt+β. Following the notions of Bronstein (2000) each of the extensions, i.e., (A,σ)≤(A[t],σ), $\left(A,\sigma )\leq (A[t,\frac{1}{t}],\sigma \right)$ or (A,σ)≤(A(t),σ) are called unimonomial extensions (of polynomial, Laurent polynomial or of rational function type, respectively).

Example 2.2

(0) Take the difference field (Q,σ) with σ(c)=c for all c∈Q.
(1) Take the unimonomial field extension (Q(k),σ) of (Q,σ) with σ(k)=k+1: Q(k) is a rational function field and *σ* is extended from Q to Q(k) with σ(k)=k+1.
(2) Take the unimonomial ring extension $\left(Q(k)[t,\frac{1}{t}],\sigma \right)$ of (Q(k),σ) with σ(t)=(k+1)t: $Q(k)[t,\frac{1}{t}]$ is a ring of Laurent polynomials with coefficients from Q(k) and the automorphism is extended from Q(k) to $Q(k)[t,\frac{1}{t}]$ with σ(t)=(k+1)t.

Finally, we consider those extensions where the constants remain unchanged.

Definition 2.3 Let (A,σ) be a difference ring.

• A unimonomial ring extension (A[t],σ) of (A,σ) with σ(t)−t∈A and constA[t]=constA is called $\Sigma^{⁎}$-ring extension (in short $\Sigma^{⁎}$-extension).
• If A is a field, a unimonomial field extension (A(t),σ) of (A,σ) with σ(t)−t∈A and constA(t)=constA is called $\Sigma^{⁎}$-field^1^ extension.
• A unimonomial ring extension $\left(A[t,\frac{1}{t}],\sigma \right)$ of (A,σ) with $\frac{\sigma (t)}{t}\in A^{⁎}$ and $\mathrm{const}\;A[t,\frac{1}{t}]=\mathrm{const}\;A$ is called Π-ring extension (in short Π-extension).
• If A is a field, a unimonomial field extension (A(t),σ) of (A,σ) with $\frac{\sigma (t)}{t}\in A^{⁎}=A(t)\setminus \{0\}$ and constA(t)=constA is called Π-field extension.

The generators of a $\Sigma^{⁎}$-extension (in the ring or field version) and a Π-extension (in the ring or field version) are called $\Sigma^{⁎}$-monomial and Π-monomial, respectively.

Remark 2.4 Keeping the constants unchanged is a central property to tackle the (parameterized) telescoping problem. E.g., if the constants are extended, there do not exist bounds on the degrees as utilized in Subsection 7.1.1. Additionally, introducing no extra constants is *the* essential property to embed the derived difference rings into the ring of sequences; this fact has been worked out, e.g., in Schneider (2010a) which is related to Hardouin and Singer (2008).

Example 2.5 **Cont. Example 2.2**

For $\left(Q,\sigma )\leq (Q(k),\sigma )\leq (Q(k)[t,\frac{1}{t}],\sigma \right)$ from Example 2.2 we have that $\mathrm{const}\;Q(k)[t,\frac{1}{t}]=\mathrm{const}\;Q(k)=\mathrm{const}\;Q=Q$, which can be checked easily. Thus (Q(k),σ) is a $\Sigma^{⁎}$-field extension of (Q,σ) and $\left(Q(k)[t,\frac{1}{t}],\sigma \right)$ is a Π-extension of (Q(k),σ). The generator *k* is a $\Sigma^{⁎}$-monomial and the generator *t* is a Π-monomial.

For more complicated extensions it is rather demanding to check if the constants remain unchanged. In this regard, we refer to the field-algorithms given in Karr (1981) or to our enhanced ring-algorithms given below which can perform these checks automatically.

For further considerations we introduce the order function ord:A→N with

(10) $$\mathrm{ord}(h)=\left\{ \begin{array}{cc} 0 & \text{if }∄n>0\text{ s.t. }h^{n}=1\\ \min\{n>0|\;h^{n}=1\} & \text{otherwise}. \end{array}\right.$$

The third type of extensions is concerned with algebraic objects like (4). Let λ∈N with λ>1, take a root of unity $\alpha \in A^{⁎}$ with $\alpha^{\lambda }=1$ and construct the unimonomial extension (A[y],σ) of (A,σ) with σ(y)=αy. Now take the ideal $I:=\langle y^{\lambda }-1\rangle$ and consider the quotient ring E=A[y]/I. Since *I* is closed under *σ*, i.e., *I* is a reflexive difference ideal (Cohn, 1965, p. 71), one can verify that σ:E→E with σ(f+I)=σ(f)+I forms a ring automorphism. In other words, (E,σ) is a difference ring. Moreover, there is the natural embedding of A into E with a→a+I. By identifying *a* with a+I, (E,σ) is a difference ring extension of (A,σ).

Lemma 2.6 *Let* (A,σ) *be a difference ring and* $\alpha \in A^{⁎}$ *with* $\alpha^{\lambda }=1$ *for some* λ>1*. Then there is (up to a difference ring isomorphism) a unique difference ring extension* (A[x],σ) *of* (A,σ) *with* x∉A *subject to the relations* $x^{\lambda }=1$ *and* σ(x)=αx*.*

Proof Consider the difference ring extension (E,σ) of (A,σ) constructed above. Define x:=y+I. Then σ(x)=αx and $x^{\lambda }=y^{\lambda }+I=1+I=1$. Further, $E=\{\sum_{i=0}^{\lambda -1}a_{i}x^{i}|a_{i}\in A\}$. Thus we obtain a difference ring extension as claimed in the lemma. Now suppose that there is another difference ring extension $\left(A[x^{\prime }],\sigma^{\prime }\right)$ of (A,σ) with $x^{\prime }\notin A$ subject to the relations $\sigma^{\prime }(x^{\prime })=\alpha \;x^{\prime }$ and $x^{\prime \;\lambda }=1$. Then by the first isomorphism theorem, there is the ring isomorphism $\tau :E\to A[x^{\prime }]$ with $\tau (\sum_{i=0}^{\lambda -1}f_{i}\;x_{i})=\sum_{i=0}^{\lambda -1}f_{i}\;x_{i}^{\prime }$. Since $\tau (\sigma (x))=\tau (\alpha \;x)=\tau (\alpha )\;\tau (x)=\alpha \;x^{\prime }=\sigma^{\prime }(x^{\prime })$, it follows that $\tau (\sigma (f))=\sigma^{\prime }(\tau (f))$ for all f∈A[x]. Summarizing, *τ* is a difference ring isomorphism.  □

The extension (A[x],σ) of (A,σ) in Lemma 2.6 is called algebraic extension of order *λ*.

Example 2.7

(0) Take the $\Sigma^{⁎}$-ext. (Q(k),σ) of (Q,σ) with σ(k)=k+1 from Example 2.5.
(1) Take the algebraic extension (Q(k)[x],σ) of (Q(k),σ) with σ(x)=−x of order 2: Q(k)[x] is an algebraic ring extension of Q(k) subject to the relation $x^{2}=1$ and *σ* is extended from Q(k) to Q(k)[x] with σ(x)=−x. Note that *x* represents the expression $X(k)={\left(-1\right)}^{k}$ with X(k+1)=−X(k).
(2) Take the algebraic extension (Q(k)[x][y],σ) of (Q(k)[x],σ) with σ(y)=−xy of order 2: Q(k)[x][y] is a ring extension of Q(k)[x] with $y^{2}=1$ and *σ* is extended from Q(k)[x] to Q(k)[x][y] with σ(y)=−xy. Note that *y* represents the expression $Y(k)={\left(-1\right)}^{\left(\begin{array}{c} k+1\\ 2 \end{array}\right)}=\prod_{j=1}^{k}{\left(-1\right)}^{j}$ with $Y(k+1)=-{\left(-1\right)}^{k}\;Y(k)$.

As for unimonomial extensions, we restrict now to those algebraic extensions where the constants remain unchanged. For the underlying motivation we refer to Remark 2.4.

Definition 2.8 Let λ∈N∖{0,1}. An algebraic extension (A[x],σ) of (A,σ) of order *λ* with constA[x]=constA is called root of unity extension (in short *R*-extension) of order *λ*. The generator *x* is called *R*-monomial.

Example 2.9 **Cont. Example 2.7**

For (Q,σ)≤(Q(k),σ)≤(Q(k)[x],σ)≤(Q(k)[x][y],σ) from Example 2.7 we have that constQ(k)[x][y]=constQ(k)[x]=constQ(k)=Q, which can be checked algorithmically; see Example 2.13 below. Thus (Q(k)[x],σ) is an *R*-extension of (Q(k),σ) and (Q(k)[x][y],σ) is an *R*-extension of (Q(k)[x],σ).

To this end, we define a tower of such extensions. First, we introduce the following notion. Let (A,σ)≤(E,σ) with t∈E. In the following A〈t〉 denotes the polynomial ring A[t] if (A[t],σ) is a $\Sigma^{⁎}$-extension of (A,σ). A〈t〉 denotes the ring of Laurent polynomials $A[t,\frac{1}{t}]$ if $\left(A[t,\frac{1}{t}],\sigma \right)$ is a Π-extension of (A,σ). Finally, A〈t〉 denotes the ring A[t] with t∉A subject to the relation $t^{\lambda }=1$ if (A[t],σ) is an *R*-extension of (A,σ) of order *λ*.

Definition 2.10 A difference ring extension (A〈t〉,σ) of (A,σ) is called $R\Pi \Sigma^{⁎}$-extension if it is an *R*-extension, Π-extension or $\Sigma^{⁎}$-extension. Analogously, it is called $R\Sigma^{⁎}$-extension, *R*Π-extension or $\Pi \Sigma^{⁎}$-extension if it is one of the corresponding extensions. More generally, $\left(G\langle t_{1}\rangle \langle t_{2}\rangle \ldots \langle t_{e}\rangle ,\sigma \right)$ is a (nested) $R\Pi \Sigma^{⁎}$-extension (resp. *R*Π, $R\Sigma^{⁎}$, $\Pi \Sigma^{⁎}$-, *R*-, Π-, $\Sigma^{⁎}$-extension) of (G,σ) if it is a tower of such extensions.

Similarly, if A is a field, (A(t),σ) is called a $\Pi \Sigma^{⁎}$-field extension if it is either a Π-field extension or a $\Sigma^{⁎}$-field extension. $\left(G(t_{1})\ldots (t_{e}),\sigma \right)$ is called a $\Pi \Sigma^{⁎}$-field extension (resp. Π-field extension, $\Sigma^{⁎}$-field extension) of (G,σ) if it is a tower of such extensions. In particular, if constG=G, $\left(G(t_{1})(t_{2})\ldots (t_{e}),\sigma \right)$ is called a $\Pi \Sigma^{⁎}$-field over G.

In both, the ring and field versions, $t_{i}$ is called $R\Pi \Sigma^{⁎}$-monomial (resp. *R*Π-, $R\Sigma^{⁎}$-, $\Pi \Sigma^{⁎}$-monomial) if it is a generator of an $R\Pi \Sigma^{⁎}$-extension (resp. *R*Π-, $R\Sigma^{⁎}$-, $\Pi \Sigma^{⁎}$-extension).

Example 2.11 **Cont. Example 2.9**

(1) (Q(k),σ) is a $\Pi \Sigma^{⁎}$-field over Q.
(2) (Q(k)〈x〉〈y〉,σ) is an *R*-extension of (Q(k),σ).

The generators with their sequential arrangement, incorporating the recursive definition of the automorphism, are always given explicitly. In particular, any reordering of the generators must respect the recursive nature induced by the automorphism.

### 2.2 A characterization of $R\Pi \Sigma^{⁎}$-extensions and their algorithmic construction

For the construction of $R\Pi \Sigma^{⁎}$-extensions we rely on the following result; for the proofs of part 1, part 2 and part 3 we refer to Proof 3.9, Proof 3.16 and Proof 3.22, respectively.

Theorem 2.12 *Let* (A,σ) *be a difference ring. Then the following holds.*

(1) *Let* (A[t],σ) *be a unimonomial ring extension of* (A,σ) *with* σ(t)=t+β *where* β∈A *such that* constA *is a field. Then this is a* $\Sigma^{⁎}$*-extension (i.e.,* constA[t]=constA*) iff there does not exist a* g∈A *with* σ(g)=g+β*.*
(2) *Let* $\left(A[t,\frac{1}{t}],\sigma \right)$ *be a unimonomial ring extension of* (A,σ) *with* σ(t)=αt *where* $\alpha \in A^{⁎}$*. Then this is a* Π*-extension (i.e.,* $\mathrm{const}\;A[t,\frac{1}{t}]=\mathrm{const}\;A$*) iff there are no* g∈A∖{0} *and* m∈Z∖{0} *with* $\sigma (g)=\alpha^{m}\;g$*. If it is a* Π*-extension,* ord(α)=0*.*
(3) *Let* (A[t],σ) *be an algebraic ring extension of* (A,σ) *of order* λ>1 *with* σ(t)=αt *where* $\alpha \in A^{⁎}$*. Then this is an R-extension (i.e.,* constA[t]=constA*) iff there are no* g∈A∖{0} *and* m∈{1,…,λ−1} *with* $\sigma (g)=\alpha^{m}\;g$*. If it is an R-extension, then α is primitive, i.e.,* ord(α)=λ*.*

For Karr's celebrated field version (Karr, 1981, 1985) of this result we refer to Theorems 3.11 and 3.18 below, that can be nicely embedded in the general difference ring framework. We emphasize that Theorem 2.12 facilitates algorithmic tactics to build difference ring extensions and to verify simultaneously if they form $R\Pi \Sigma^{⁎}$-extensions. Here we consider two cases.

#### 2.2.1 Testing and constructing *R*Π-extensions

Let (A,σ) be a difference ring and let α∈A. Then we want to decide if we can construct an *R*Π-extension (A〈t〉,σ) of (A,σ) with σ(t)=αt. First, we have to check if $\alpha \in A^{⁎}$. E.g., for the class of difference rings (A,σ), built by simple $R\Pi \Sigma^{⁎}$-extensions introduced in Definition 2.19 below, this task will be straightforward. Next, we need the order of *α*, i.e., we have to solve the following Problem O with $G:=A^{⁎}$.

**Problem O in** *G***.** *Given* a group *G* and α∈G. *Find* ord(α).

Given λ=ord(α), we can decide which case has to be treated. If λ=0, only the construction of a Π-extension might be possible due to Theorem 2.12. Thus we construct the unimonomial extension $\left(A[t,\frac{1}{t}],\sigma \right)$ of (A,σ) with σ(t)=αt. Otherwise, if λ>0, we construct the algebraic extension (A[t],σ) of (A,σ) with σ(t)=αt of order *λ*. Finally, we check if our construction is indeed a Π-extension or *R*-extension, i.e., if the constants remain unchanged. Using Theorem 2.12 this test can be accomplished by solving

**Problem MT in** (A,σ)**.** *Given* a difference ring (A,σ) and $\alpha \in A^{⁎}$ with λ=ord(α). *Decide* if there are a g∈A∖{0} and an m∈Z∖{0} for the case λ=0 (resp. m∈{1,…,λ−1} for the case λ>0) such that the multiplicative version of the telescoping equation (MT) holds:

(11) $$\sigma (g)=\alpha^{m}\;g.$$

More generally, if we are given a tower of algebraic and unimonomial extensions, which model indefinite nested products, Problem MT can be used to check if the construction constitutes a nested *R*Π-extension.

Example 2.13 **Cont. Example 2.9**

We will verify that (Q(k)[x][y],σ) is an *R*-extension of (Q(k),σ).

(1) Take α=−1 with λ=ord(α)=2. We solve Problem MP by the algorithms presented below: there are no $g\in Q{\left(k\right)}^{⁎}$ and m∈{1} with $\sigma (g)={\left(-1\right)}^{m}\;g$. Hence by Theorem 2.12.(3)^2^ (Q(k)[x],σ) is an *R*-extension of (Q(k),σ).
(2) Now we solve Problem O for α=−x and get λ=ord(−x)=2; see Example 5.4.(2). In addition, solving Problem MP for *α* shows that there is no g∈Q(k)[x]∖{0} with σ(g)=−xg. Thus by Theorem 2.12.(3) (Q(k)[x][y],σ) forms an *R*-extension of (Q(k)[x],σ).

Example 2.14 We construct a ring in which the objects in (7) can be represented.

(0) Take the $\Pi \Sigma^{⁎}$-field (K(k),σ) over K=Q(ι) with σ(k)=k+1.
(1) Take α=ι. Then solving Problem O provides λ=ord(α)=4. In particular solving the corresponding Problem MP proves that there are no $g\in K{\left(k\right)}^{⁎}$ and m∈{1,2,3} with (11). Hence by Theorem 2.12.(3) we can construct the *R*-extension (K(k)[x],σ) of (K(k),σ) with σ(x)=ιx. Note that the *R*-monomial *x* represents $\iota^{k}$.
(2) Take α=xk. Solving Problem O yields λ=ord(α)=0 and solving Problem MP shows that there are no g∈K(k)[x]∖{0} and m∈Z∖{0} with (11). With Theorem 2.12.(2) we can construct the Π-extension (K(k)[x],σ)≤(K(k)[x]〈t〉,σ) with σ(t)=xkt; here the Π-monomial *t* represents $\prod_{j=1}^{k-1}j\iota^{j}$.

#### 2.2.2 Testing and constructing $\Sigma^{⁎}$-extensions

In order to verify if a unimonomial extension as given in Theorem 2.12.(1) is a $\Sigma^{⁎}$-extension, it suffices to solve Problem T with f=β and to check if there is not a telescoping solution. We illustrate this feature by actually constructing a difference ring in which the summand

(12) $$f(k)={\left(-1\right)}^{\left(\begin{array}{c} k+1\\ 2 \end{array}\right)}k^{2}\sum_{j=1}^{k}\frac{{\left(-1\right)}^{j}}{j}$$

given on the left hand side of (6) and the additional sum

(13) $$\sum_{j=1}^{k}\frac{{\left(-1\right)}^{\left(\begin{array}{c} j+1\\ 2 \end{array}\right)}}{j}$$

occurring on the right hand side of (6) can be represented. In particular, we demonstrate how identity (6) can be discovered in this difference ring.

Example 2.15 **Cont. Example 2.9**

(0) Take the difference ring (A,σ) with A=Q(k)[x][y].
(1) Take $f=\sigma (\frac{x}{k})=\frac{-x}{k+1}$. Then solving Problem T shows that there is no g∈A with $\sigma (g)-g=\frac{-x}{k+1}$. Hence we can construct the $\Sigma^{⁎}$-extension (A[s],σ) of (A,σ) with $\sigma (s)=s+\frac{-x}{k+1}$; note that the $\Sigma^{⁎}$-monomial *s* represents $\sum_{j=1}^{k}\frac{{\left(-1\right)}^{j}}{j}$.
(2) Take $f=\sigma (\frac{y}{k})=\frac{-x\;y}{k+1}$. Then solving Problem T shows that there is no g∈A[s] with $\sigma (g)-g=\frac{-x\;y}{k+1}$. Hence we can construct the $\Sigma^{⁎}$-extension (A[s][S],σ) of (A[s],σ) with $\sigma (S)=S+\frac{-x\;y}{k+1}$; note that the $\Sigma^{⁎}$-monomial *S* represents the sum (13).
(3) Take $f=y\;k^{2}\;s$ which represents (12). Solving Problem T produces the solution
  (14) $$g=sy(\frac{1}{2}(k-1)(k+1)x-\frac{1}{2}(k-2)k)+y(\frac{1}{4}(1-2k)-\frac{1}{4}x)+\frac{1}{2}S;$$
  for further details see Example 7.3. Hence this yields the solution of the telescoping equation (1) for our summand (12) by replacing the $R\Sigma^{⁎}$-monomials *x*, *y*, *s*, *S* with the corresponding summation objects. Taking a=1 in (2) and performing the evaluation c:=g(1)=0∈Q gives the identity (6).
(4) Note that we succeeded in representing the sum $F(k)=\sum_{i=1}^{k}f(i)$ with *f* from (12) in the difference ring in A[s][S] with σ(g)−c=σ(g). Namely, replacing the variables in σ(g) with the corresponding summation objects yields the right hand side of (6). This is of particular interest if there are further sums defined over F(k) which one wants to represent in a $\Sigma^{⁎}$-extension over (A[s][S],σ).

We remark that for the derivation of the identity (6) it is crucial to introduce the extra sum (13). Here this was accomplished manually. But, using algorithms from Schneider (2008, 2015) in combination with the results of this article, this sum can be determined automatically.

#### 2.2.3 The underlying problems for $R\Pi \Sigma^{⁎}$-extensions

As in the difference field approach (Karr, 1981; Schneider, 2005d, 2008, 2015), Problem T and more generally Problem PT will be solved by reducing them from (A,σ) to smaller difference rings (i.e., rings built by less $R\Pi \Sigma^{⁎}$-monomials). Likewise, this reduction technique can be applied in order to solve a special case of Problem MT that will cover all the cases needed for our difference ring constructions. However, in order to carry out these reductions, one has to tackle generalized problems within the recursion steps.

For Problem MT the following generalization is needed. Let (A,σ) be a difference ring, let W⊆A and let $f=(f_{1},\ldots ,f_{n})\in {\left(A^{⁎}\right)}^{n}$. Then we define the set (Karr, 1981)

$$M(f,W):=\{(m_{1},\ldots ,m_{n})\in Z^{n}|\;\sigma (g)=f_{1}^{m_{1}}\ldots f_{n}^{m_{n}}\;g\text{ for some }g\in W\setminus \{0\}\}.$$

In the following, we want to calculate a finite representation of M(f,A). If A is a field, i.e., $A^{⁎}=A\setminus \{0\}$, it is immediate that M(f,A) is a submodule of $Z^{n}$ over Z and there is a basis of M(f,A) with rank ≤*n*; see Karr (1981). In the setting of rings, this result carries over if the set of semi-constants (also called semi-invariants, Bronstein, 2000) of (A,σ) defined by

$$\mathrm{sconst}(A,\sigma )=\{c\in A|\sigma (c)=u\;c\text{ for some }u\in A^{⁎}\}$$

forms a multiplicative group (excluding the 0 element). Note: if A is a field, we have that $\mathrm{sconst}(A,\sigma )\setminus \{0\}=A\setminus \{0\}=A^{⁎}$. Unfortunately, for a general difference ring the set sconst(A,σ)∖{0} is only a multiplicative monoid (Bronstein, 2000). In order to gain more flexibility, we introduce the following refinement. For a given multiplicative subgroup *G* of $A^{⁎}$ (in short $G\leq A^{⁎}$), we define the set of semi-constants (semi-invariants) of (A,σ) over *G* by

$${\mathrm{sconst}}_{G}(A,\sigma )=\{c\in A|\sigma (c)=u\;c\text{ for some }u\in G\}.$$

Note that ${\mathrm{sconst}}_{\left(A^{⁎}\right)}(A,\sigma )=\mathrm{sconst}(A,\sigma )$ and ${\mathrm{sconst}}_{\left\{1\right\}}(A,\sigma )=\mathrm{const}(A,\sigma )$. If it is clear from the context, we drop *σ* and just write ${\mathrm{sconst}}_{G}\;A$ and sconstA, respectively.

Here is one of the main challenges: For all our considerations we will choose *G* such that ${\mathrm{sconst}}_{G}\;A\setminus \{0\}$ is a subgroup of $A^{⁎}$ (in short, $\mathrm{sconst}\;A\setminus \{0\}\leq A^{⁎}$). Then with this careful choice of *G* we can summarize the above considerations with the following lemma.

Lemma 2.16 *Let* (A,σ) *be a difference ring and let* $G\leq A^{⁎}$ *with* ${\mathrm{sconst}}_{G}\;A\setminus \{0\}\leq A^{⁎}$*; let* $f\in G^{n}$*. Then* $M(f,A)=M(f,{\mathrm{sconst}}_{G}\;A)$*. In particular,* M(f,A) *is a submodule of* $Z^{n}$ *over* Z*, and it has a finite* Z*-basis with rank* ≤*n.*

In the light of this property, we can state Problem PMT.

**Problem PMT in** (A,σ) **for** *G***.** *Given* a difference ring (A,σ) with $G\leq A^{⁎}$ such that ${\mathrm{sconst}}_{G}\;A\setminus \{0\}\leq A^{⁎}$ holds; given $f\in G^{n}$. *Find* a Z-basis of M(f,A).

Observe that Problem MT can be reduced to Problem PMT for a group *G* with ${\mathrm{sconst}}_{G}\;A\setminus \{0\}\leq A^{⁎}$ if we restrict^3^ to the situation that α∈G. More precisely, assume that we have calculated λ=ord(α) and succeeded in solving Problem PMT, i.e., we are given a basis of $M=M((\alpha ),A)\subseteq Z^{1}$. If the basis is empty, there cannot be an m∈Z∖{0} and a g∈A∖{0} with (11). Otherwise, if the basis is not empty, the rank is 1. More precisely, we obtain m>0 with M=mZ. Hence *m* is the smallest positive choice such that there is a g∈A∖{0} with (11). Therefore we can again decide^4^ Problem MT.

For the generalization of Problems T and PT we introduce the following set. Let (A,σ) be a difference ring with constant field K, let W⊆A, and let u∈A∖{0} and $f=(f_{1},\ldots ,f_{n})\in A^{n}$. Then we define (Karr, 1981)

$$V(u,f,(W,\sigma ))=\{(c_{1},\ldots ,c_{n},g)\in K^{n}\times W|\;\sigma (g)-u\;g=c_{1}\;f_{1}+\cdots +c_{n}\;f_{n}\};$$

if it is clear from the context, we write V(u,f,W) and suppress the automorphism *σ*.

As with Lemma 2.16 the following result will be crucial for further considerations.

Lemma 2.17 *Let* (A,σ) *be a difference ring with constant field* K *and let* $G\leq A^{⁎}$ *with* ${\mathrm{sconst}}_{G}\;A\setminus \{0\}\leq A^{⁎}$*. Let W be a* K*-subspace of* A*. Then for* $f\in A^{n}$ *and* u∈G *we have that* V(u,f,W) *is a* K*-subspace of* $K^{n}\times W$ *with* dim⁡V(u,f,W)≤n+1*.*

Proof Suppose that there are *m* linearly independent solutions with m>n+1, say $\left(c_{i,1},\ldots ,c_{i,n},g_{i}\right)$ with 1≤i≤m. Then by row operations over the field K we can derive at least two linearly independent vectors, say $v_{1}=(0,\ldots ,0,g)$ and $v_{2}=(0,\ldots ,0,h)$. Hence we have that σ(g)=ug and σ(h)=uh where $g,h\in {\mathrm{sconst}}_{G}\;A\setminus \{0\}\leq A^{⁎}$. Consequently, $\sigma (\frac{g}{h})=\frac{g}{h}$, thus $c=g/h\in K^{⁎}$ and therefore $v_{1}=c\;v_{2}$; a contradiction that the vectors are linearly independent.  □

This result gives rise to the following problem specification.

**Problem PFLDE in** (A,σ) **for** *G* **(with constant field** K**).** *Given* a difference ring (A,σ) with constant field K and $G\leq A^{⁎}$ such that ${\mathrm{sconst}}_{G}\;A\setminus \{0\}\leq A^{⁎}$ holds; given u∈G and $f\in A^{n}$. *Find* a K-basis of V(u,f,A).

In particular, if we can solve Problem PFLDE in (A,σ) for *G*, it follows with 1∈G that we can solve Problems T and PT in (A,σ). Furthermore, we can solve the multiplicative version of telescoping: if α∈G, we can determine a g∈A∖{0}, in case of existence, such that σ(g)=αg holds. This feature is illustrated by the following example.

Example 2.18 **Cont. Example 2.14**

Given $Q(b)=\prod_{k=1}^{b}\frac{-(\iota^{k})}{1+k}$ on the left hand side of (7), we want to rewrite it in terms of the product $P(b)=\prod_{j=1}^{b-1}j\iota^{j}$. In a preparation step we constructed already the $R\Pi \Sigma^{⁎}$-extension (K(k)[x]〈t〉,σ) of (K(k),σ) with K=Q(ι), σ(x)=ιx and σ(t)=kxt in Example 2.14. There we can represent $\frac{-(\iota^{k})}{k+1}$ with $u=\frac{-x}{k+1}$ and P(k) with *t*. Now we search for a g∈K(k)[x]〈t〉∖{0} such that σ(g)=ug holds. More precisely, we are interested in a basis of V=V(u,(0),K(k)[x]〈t〉). Activating our machinery, we get the basis {(0,g),(1,0)} of *V* with $g=\frac{x(\iota +x^{2})}{k}t^{-1}$. For the chosen group *G* with u∈G, that we use to solve the underlying Problem PFLDE in (K(k)[x]〈t〉,σ), and the corresponding calculation steps we refer to Example 7.6 below. Since *g* is a solution of σ(g)=ug, $g(k)=(\iota +{\left(-1\right)}^{k})\frac{\iota^{k}}{k}P{\left(k\right)}^{-1}$ is a solution of $-\frac{\iota^{k}}{k+1}=\frac{g(k+1)}{g(k)}$. Hence by the telescoping trick we get $\prod_{k=1}^{b}-\frac{\iota^{k}}{k+1}=\frac{g(b+1)}{g(1)}$ which produces (7).

### 2.3 The main results

Suppose that we are given a difference ring (G,σ) which is computable and we are given a group $G\leq G^{⁎}$ with ${\mathrm{sconst}}_{G}\;G\setminus \{0\}\leq G^{⁎}$. In this article we will restrict to certain classes of $R\Pi \Sigma^{⁎}$-extensions (E,σ) of (G,σ) equipped with a group $\overset{˜}{G}$ with $G\leq \overset{˜}{G}\leq E^{⁎}$ and ${\mathrm{sconst}}_{\overset{˜}{G}}\;E\setminus \{0\}\leq E^{⁎}$ such that we can derive the following algorithmic machinery:

(1) Problem O in $\overset{˜}{G}$ can be reduced to Problem O in *G*;
(2) Problem PMT in (E,σ) for $\overset{˜}{G}$ can be reduced to Problem PMT in (G,σ) for *G*;
(3) Problem PFLDE in (E,σ) for $\overset{˜}{G}$ can be reduced to Problem PFLDE in (G,σ) for *G* (see Subsection 2.3.1) or to Problem PFLDE in $\left(G,\sigma^{k}\right)$ for *G* for all k≥1 (see Subsection 2.3.2).

In a nutshell, if we choose as base case a difference ring (G,σ) and a group $G\leq G^{⁎}$ in which we can solve Problem O in *G* and Problems PMT and PFLDE in (G,σ) for *G* (resp. $\left(G,\sigma^{k}\right)$ for *G* for all k≥1), we obtain recursive algorithms that solve the corresponding problems in the larger difference ring (E,σ) and larger group $\overset{˜}{G}$.

As it turns out, we will succeed in this task for a subclass of $R\Pi \Sigma^{⁎}$-extensions (G,σ)≤(E,σ) and a properly chosen group $\overset{˜}{G}\leq E^{⁎}$ that can treat all objects (among the general class of $R\Pi \Sigma^{⁎}$-extensions) that the author has encountered in practical problem solving so far. More precisely, we will restrict to simple $R\Pi \Sigma^{⁎}$-extensions.

Let $\left(G\langle t_{1}\rangle \ldots \langle t_{e}\rangle ,\sigma \right)$ be an $R\Pi \Sigma^{⁎}$-extension of (G,σ) and let $G\leq G^{⁎}$. Then we define

(15) $$G_{G}^{E}=\{gt_{1}^{m_{1}}\ldots t_{e}^{m_{e}}|\;h\in G\text{ and }m_{i}\in Z\text{ where }m_{i}=0\text{ if }t_{i}\text{ is a }\Sigma^{⁎}\text{-monomial}\}.$$

It is easy to see that $\overset{˜}{G}=G_{G}^{E}$ forms a group. More precisely, we obtain the following chain of subgroups: $G\leq G_{G}^{E}\leq E^{⁎}$. We call $G_{G}^{E}$ also the product-group over *G* for the $R\Pi \Sigma^{⁎}$-extension (E,σ) of (G,σ). We are now ready to define (*G*-)simple $R\Pi \Sigma^{⁎}$-extensions.

Definition 2.19 Let (G,σ) be a difference ring and let $G\leq G^{⁎}$ be a group. An $R\Pi \Sigma^{⁎}$-extension (E,σ) of (G,σ) with $E=G\langle t_{1}\rangle \langle t_{2}\rangle \ldots \langle t_{e}\rangle$ is called *G*-simple if for any *R*Π-monomial $t_{i}$ we have that $\sigma (t_{i})/t_{i}\in G_{G}^{E}$. Moreover, an *R*Π, $R\Sigma^{⁎}$, $\Pi \Sigma^{⁎}$-, *R*-, Π-, and $\Sigma^{⁎}$-extension of (G,σ) is *G*-simple if it is a *G*-simple $R\Pi \Sigma^{⁎}$-extension. We call any such extension simple if it is $G^{⁎}$-simple. Analogously, we call an *R*Π, $R\Sigma^{⁎}$, $\Pi \Sigma^{⁎}$-, *R*-, Π-, and $\Sigma^{⁎}$-monomial *G*-simple (resp. simple) if the extension is *G*-simple (resp. simple).

In all our examples the difference rings have been built by a simple $R\Pi \Sigma^{⁎}$-extension (E,σ) of (G,σ) where (G,σ) is a $\Pi \Sigma^{⁎}$-field (K(k),σ) over K with σ(k)=k+1. In particular, the Problems PMT and PFLDE have been considered for the constructed (E,σ) in $G={\left(K{\left(k\right)}^{⁎}\right)}_{K(k)}^{E}$. Before we finally turn to the class of simple $R\Pi \Sigma^{⁎}$-extensions, we present one example which cannot be treated properly with our toolbox under consideration.

Example 2.20 Take $\left(Q(k)[t,\frac{1}{t}],\sigma \right)$ from Example 2.5 with σ(k)=k+1 and σ(t)=(k+1)t. Subsequently, we will use our notation $Q(k)\langle t\rangle =Q(k)[t,\frac{1}{t}]$. Then we can construct the *R*-extension (Q(k)〈t〉[x],σ) of (Q(k)〈t〉,σ) with σ(x)=−x of order 2. In this ring we are given the idempotent elements $e_{1}=(1-x)/2$ and $e_{2}=(x+1)/2$ with $e_{1}^{2}=e_{1}$ and $e_{2}^{2}=e_{2}$. Finally take $\alpha =e_{1}+e_{2}\;t$. Then observe that $\alpha \cdot (e_{1}+e_{2}/t)=1$, i.e., $\alpha \in Q(k)\langle t\rangle {\left[x\right]}^{⁎}$. Note that ord(α)=0. Otherwise it would follow that $e_{2}^{\lambda }=0$ with λ=ord(α)>0; a contradiction that $e_{2}$ is idempotent. Consequently, *T* cannot be an *R*-extension, and we construct the unimonomial extension $\left(Q(k)\langle t\rangle [x][T,\frac{1}{T}],\sigma \right)$ of (Q(k)〈t〉[x],σ) with σ(T)=αT. It seems non-trivial to derive an (algorithmic) proof (or disproof) that *T* is a Π-monomial, and it would be nice to see a solution to this problem.

Summarizing, we aim at solving Problems PMT and PFLDE in a *G*-simple $R\Pi \Sigma^{⁎}$-extension (G,σ)≤(E,σ) for $\overset{˜}{G}=G_{G}^{E}$, and we want to solve Problem O in $\overset{˜}{G}$. In order to accomplish this task, we will restrict ourselves further to the following two situations.

#### 2.3.1 A solution for single-rooted $R\Pi \Sigma^{⁎}$-extensions

In most applications *R*-extensions are not nested, e.g., only objects like ${\left(-1\right)}^{k}$ arise. In addition, such objects do not occur in transcendental products, but only in sums, like cyclotomic sums (Ablinger et al., 2011) or generalized harmonic sums (Ablinger et al., 2013). A formal definition of this special, but very practical oriented class of $R\Pi \Sigma^{⁎}$-extensions is as follows.

Definition 2.21 An $R\Pi \Sigma^{⁎}$-extension (E,σ) of (G,σ) is called single-rooted if the generators of the extension can be reordered to

(16) $$E=G\langle t_{1}\rangle \ldots \langle t_{r}\rangle \langle x_{1}\rangle \ldots \langle x_{u}\rangle \langle s_{1}\rangle \ldots \langle s_{v}\rangle ,$$

respecting the recursive nature of the automorphism, such that the $t_{i}$ are Π-monomials, the $x_{i}$ are *R*-monomials with $\sigma (x_{i})/x_{i}\in G^{⁎}$ and the $s_{i}$ are $\Sigma^{⁎}$-monomials.

Given this class of single-rooted and simple^5^ $R\Pi \Sigma^{⁎}$-extension, we will show the following theorem in Proof 4.8.

Theorem 2.22 *Let* (G,σ) *be a difference ring and let* $G\leq G^{⁎}$ *with* ${\mathrm{sconst}}_{G}\;G\setminus \{0\}\leq G^{⁎}$*. Let* (E,σ) *be a simple and single-rooted* $R\Pi \Sigma^{⁎}$*-extension of* (G,σ) *with* (16) *as specified in* Definition 2.21*, and let* $\overset{˜}{G}=G_{G}^{G\langle t_{1}\rangle \ldots \langle t_{r}\rangle }$*. Then* ${\mathrm{sconst}}_{\overset{˜}{G}}\;E\setminus \{0\}\leq E^{⁎}$ *with*

$${\mathrm{sconst}}_{\overset{˜}{G}}\;E=\{h\;t_{1}^{m_{1}}\ldots t_{r}^{m_{r}}\;x_{1}^{n_{1}}\ldots x_{u}^{n_{u}}|\;h\in {\mathrm{sconst}}_{G}\;G,m_{i}\in Z\text{and}n_{i}\in N\}.$$

In particular, we obtain the following reduction algorithms summarized in Theorem 2.23; for a proof of part 1 see Proof 6.7 and of part 2 see Proof 7.10.

Theorem 2.23 *Let* (G,σ) *be a computable difference ring with* $G\leq G^{⁎}$ *and* ${\mathrm{sconst}}_{G}\;G\setminus \{0\}\leq G^{⁎}$*. Let* (E,σ) *be a single-rooted and G-simple* $R\Pi \Sigma^{⁎}$*-extension of* (G,σ) *with* (16) *as given in* Definition 2.21*, and let* $\overset{˜}{G}=G_{G}^{G\langle t_{1}\rangle \ldots \langle t_{r}\rangle }$*. Then the following holds.*

(1) *Problem PMT is solvable in* (E,σ) *for* $\overset{˜}{G}$ *if it is solvable in* (G,σ) *for G.*
(2) *Problem PFLDE is solvable in* (E,σ) *for* $\overset{˜}{G}$ *if Problems PFLDE and PMT are solvable in* (G,σ) *for G and if*^6^ *Problem O is solvable in G.*

All the calculations in Schneider (2004b, 2007a), Prodinger et al. (2011), Osburn and Schneider (2009), Blümlein et al. (2013), Ablinger et al., (2014a, 2014b) rely precisely on this machinery. For one of the most important applications we refer to Subsection 2.4.

#### 2.3.2 A solution for simple $R\Pi \Sigma^{⁎}$-extensions of a strong constant-stable difference field

In the following we restrict to simple $R\Pi \Sigma^{⁎}$-extensions where the ground domain G=F is a field. In this setting, the semi-constants form a multiplicative group. More precisely, we will show the following result in Proof 4.11.

Theorem 2.24 *Let* (E,σ) *be a simple* $R\Pi \Sigma^{⁎}$*-extension of a difference field* (F,σ) *and consider its product-group* $\overset{˜}{G}={\left(F^{⁎}\right)}_{F}^{E}$*. Then* ${\mathrm{sconst}}_{\overset{˜}{G}}\;E\setminus \{0\}\leq E^{⁎}$*.*

For a solution of Problems PMT and PFLDE we require in addition that (F,σ) is strong constant-stable.

Definition 2.25 A difference ring (A,σ) with constant field K is called constant-stable if for all k>0 we have that $\mathrm{const}(A,\sigma^{k})=K$. It is called strong constant-stable if it is constant-stable and any root of unity of A is in K.

In this setting we can treat products over roots of unity from K and, more generally, products that are built recursively over such products; for examples see (4) and for further (algorithmic) properties see Corollary 5.6 below. More precisely, given such a tower of $R\Pi \Sigma^{⁎}$-extensions, we can solve Problems PMT and PFLDE as follows; for the proofs, resp. the underlying algorithms, of part 1 see Proof 5.7, of part 2 see Proof 6.15 and of part 3 see Proof 7.16.

Theorem 2.26 *Let* (F,σ) *be a computable difference field where Problem O is solvable in* ${\left(\mathrm{const}\;F\right)}^{⁎}$*. Let* (E,σ) *be a simple* $R\Pi \Sigma^{⁎}$*-extension of* (F,σ)*. Then the following holds.*

(1) *Problem O is solvable in* ${\left(F^{⁎}\right)}_{F}^{E}$*.*

*If* (F,σ) *is in addition strong constant-stable, then*

(2) *Problem PMT is solvable in* (E,σ) *for* ${\left(F^{⁎}\right)}_{F}^{E}$ *if it is solvable in* (F,σ) *for* $F^{⁎}$*;*
(3) *Problem PFLDE is solvable in* (E,σ) *for* ${\left(F^{⁎}\right)}_{F}^{E}$ *if Problem PMT is solvable in* (F,σ) *for* $F^{⁎}$ *and Problem PFLDE is solvable*^7^ *in* $\left(F,\sigma^{k}\right)$ *for* $F^{⁎}$ *for all* k>0*.*

We remark that this reduction machinery has been utilized in Examples 2.15 and 2.18 to obtain the identities (6) and (7), respectively. Further details will be given below.

#### 2.3.3 A complete machinery: algorithms for the ground difference rings

Both, Theorems 2.23 and 2.26 provide algorithms to reduce the Problems PMT and PFLDE (and thus the Problems T, PT and special cases of Problem MT) from an $R\Pi \Sigma^{⁎}$-extension (E,σ) of (G,σ) to the ground difference ring (G,σ). Theorem 2.23 requires less conditions on (G,σ), but considers only single-rooted $R\Pi \Sigma^{⁎}$-extensions, whereas Theorem 2.26 requires more properties on (G,σ) but allows nested *R*-extensions which are of the type as given in Corollary 5.6 below. Note that the algorithms for the latter case are more demanding, in particular, one has to solve Problem PFLDE in $\left(G,\sigma^{k}\right)$ with k>0 instead of k=1 only.

We emphasize that both theorems are applicable for a rather general class of difference fields (G,σ). Namely, (G,σ) itself can be a $\Pi \Sigma^{⁎}$-field extension of (H,σ) where certain properties in the difference field (H,σ) hold. Here the following remarks are in place.

(a) By Karr (1981) a $\Pi \Sigma^{⁎}$-field extension (G,σ) of (H,σ) is constant-stable if (H,σ) is constant-stable. In particular, if we are given a root of unity from G, it cannot depend on transcendental elements and is therefore from H. Thus (G,σ) is strong constant-stable if (H,σ) is strong constant-stable.
(b) It has been shown in Kauers and Schneider (2006b) that one can solve Problem PMT in (G,σ) for $G^{⁎}$ and Problem PFLDE in $\left(G,\sigma^{k}\right)$ for $G^{⁎}$ for k>0 if certain properties hold for the difference field (H,σ). Among others (see Def. 1 and 2 in Kauers and Schneider, 2006b) Problem PMT must be solvable in (H,σ) for $H^{⁎}$ and Problem PFLDE must be solvable in $\left(H,\sigma^{k}\right)$ for $H^{⁎}$.

Summarizing, if we are given the tower of extensions

$$\left(H,\sigma )\overset{\Pi \Sigma^{⁎}\text{-}\text{field}\;\mathrm{ext}.}{\leq }(G,\sigma )\overset{R\Pi \Sigma^{⁎}\text{-}\text{ring}\;\mathrm{ext}.}{\leq }(E,\sigma \right)$$

where (H,σ) is strong constant-stable and the properties given in Def. 1 and 2 of Kauers and Schneider (2006b) hold in (H,σ), then we can solve Problems PMT and PFLDE in (E,σ) for ${\left(G^{⁎}\right)}_{G}^{E}$.

So far, the required properties have been verified and the necessary algorithms have been worked out for the following difference fields (H,σ) with constant field K.

(1) K=H, i.e., (G,σ) is a $\Pi \Sigma^{⁎}$-field over K; here the constant field K can be a rational function field over an algebraic number field; see Schneider (2005c, Theorem 3.5).
(2) (H,σ) is a free difference field, i.e., $H=K(\ldots ,x_{-1},x_{0},x_{1},\ldots )$ with $\sigma (x_{i})=x_{i+1}$; here K is of the type as given in case (1). Note that in this field one can model unspecified sequences; see Kauers and Schneider (2006a, 2006b).
(3) (H,σ) can be a radical difference field representing objects like $\sqrt[{d}]{k}$; see Kauers and Schneider (2007).

For simplicity, all our examples are chosen from case (1). More precisely, we always take the $\Pi \Sigma^{⁎}$-field (H,σ)=(K(k),σ) over K∈{Q,Q(ι)} with σ(k)=k+1.

### 2.4 Application: representation of d'Alembertian solutions in $R\Pi \Sigma^{⁎}$-extensions

We illustrate how an important class of d'Alembertian solutions (Abramov and Petkovšek, 1994), a subclass of Liouvillian solutions (Hendriks and Singer, 1999; Petkovšek and Zakrajšek, 2013), of a given linear difference operator, can be represented completely automatically in $R\Pi \Sigma^{⁎}$-extensions. In order to obtain the d'Alembertian solutions, one starts as follows: first the linear difference operator is factored as much as possible into linear right hand factors. This can be accomplished, e.g., with the algorithms from Petkovšek (1992), van Hoeij (1999), Horn et al. (2012) or, within the setting of $\Pi \Sigma^{⁎}$-fields with the algorithms given in Abramov et al. (in preparation) which are based on Bronstein (2000), Schneider (2001, 2005d). The latter machinery is available within the summation package Sigma. Then given this factored form of the operator, the d'Alembertian solutions can be read off. They can be given by a finite number of hypergeometric expressions and indefinite nested sums defined over such expressions. More precisely, each solution is of the form

(17) $$\sum_{i_{1}=\lambda_{1}}^{k}h_{1}(i_{1})\sum_{i_{2}=\lambda_{2}}^{i_{1}}h_{2}(i_{2})\cdots \sum_{i_{r}=\lambda_{r-1}}^{i_{r-1}}h_{r}(i_{r})$$

where $\lambda_{i}\in N$ and the hypergeometric expression $h_{i}(k)$ can be written in the form $\prod_{j=\lambda_{i}}^{k}\alpha_{i}(j)$ with $\alpha_{i}(z)$ being a rational function from K(z).

Subsequently, we restrict ourselves to a field K which is a rational function field $K=Q(n_{1},\ldots ,n_{r})$ over the rational numbers. Now take the $\Pi \Sigma^{⁎}$-field (K(k),σ) over K with σ(k)=k+1. Then the solutions, all being of the form (17), can be represented in a single-rooted simple $R\Pi \Sigma^{⁎}$-extension as follows.

(1) In Schneider (2005c, Section 6) an algorithm has been presented that calculates a single-rooted simple *R*Π-extension (G,σ) of (K(k),σ) in which all hypergeometric expressions occurring in the d'Alembertian solutions are explicitly represented.

(2) Then the challenging task is to construct a $\Sigma^{⁎}$-extension of (G,σ) and to represent there the arising sums of the d'Alembertian solutions. Given (G,σ) from step 1, this can be accomplished by applying iteratively Theorem 2.12.(1). Suppose we represented already an inner summand in a $\Sigma^{⁎}$-extension (A,σ) of (G,σ) with β∈A. Since (A,σ) is a simple $R\Pi \Sigma^{⁎}$-extension of (K(k),σ) and (K(k),σ) is a $\Pi \Sigma^{⁎}$-field over K, we can solve Problem T with f=β by using the underlying algorithm of Theorem 2.23 in combination with the base case algorithms; see Subsection 2.3.3. If we find a g∈A with σ(g)=g+β, we can represent the sum under consideration with g+c where c∈K is determined by the boundary condition (lower summation bound) of the given sum; for further details we refer to Example 2.15.(4). Otherwise, we construct the $\Sigma^{⁎}$-extension (A[t],σ) of (A,σ) with σ(t)=t+β by Theorem 2.12.(1) and we succeeded in representing the sum under consideration by *t* with the appropriate shift behavior. Note that (A[t],σ) is again a single-rooted simple $R\Pi \Sigma^{⁎}$-extension of (K(k),σ). Proceeding iteratively, all the nested hypergeometric sums are represented in terms of an $R\Pi \Sigma^{⁎}$-extension over (K(k),σ).

Exactly this difference ring machinery is implemented in Sigma and has been used to tackle challenging applications, like Schneider (2004b, 2007a), Prodinger et al. (2011), Osburn and Schneider (2009), Blümlein et al. (2013), Ablinger et al., (2014a, 2014b) mentioned already in the introduction. In particular, this toolbox has been combined with the algorithms worked out in Schneider (2004c, 2005c, 2007b, 2008, 2015), Abramov and Petkovšek (2010) in order to find representations of d'Alembertian solutions with certain optimality properties, like minimal nesting depth. For a recent summary of all these features (unfortunately, in the setting of difference fields) we refer to Schneider (2013, 2014).

## 3 Single nested $R\Pi \Sigma^{⁎}$-extensions

This section delivers relevant properties of single nested $R\Pi \Sigma^{⁎}$-extensions. The characterization of $R\Pi \Sigma^{⁎}$-extensions (Theorem 2.12) will be elaborated. In addition, properties of the semi-constants within $R\Pi \Sigma^{⁎}$-extensions are derived to gain further insight in the nature of $R\Pi \Sigma^{⁎}$-extensions and to prove Theorems 2.22 and 2.24 in Section 4.

We start with some general properties which will be essential throughout this article.

Definition 3.1 A ring A is called reduced if there are no non-zero nilpotent elements, i.e., for any f∈A∖{0} and any n>0 we have that $f^{n}\neq 0$. A is called connected if 0 and 1 are the only idempotent elements, i.e., for any f∈A∖{0,1} we have that $f^{2}\neq f$.

Namely, we rely on the following ring properties. A polynomial $\sum_{i=0}^{n}a_{i}x^{i}\in A[t]$ with coefficients from a ring A is invertible if and only if $a_{0}\in A^{⁎}$ and $a_{i}$ with i≥1 are nilpotent elements. Thus in a reduced ring, i.e., a ring which has no nilpotent non-zero elements, we have that $A{\left[t\right]}^{⁎}=A^{⁎}$. Besides, there is a complete characterization of invertible elements in the ring of Laurent polynomials $A[t,\frac{1}{t}]$ presented in Karpilovsky (1983, Theorem 1) (see also Neher, 2009). Based on this work we extract the following crucial result.

Lemma 3.2 *Let* A *be a commutative ring with 1. If* A *is reduced, then* $A{\left[t\right]}^{⁎}=A^{⁎}$*. If* A *is reduced and connected, then* $A{\left[t,\frac{1}{t}\right]}^{⁎}=\{u\;t^{r}|\;u\in A^{⁎}\text{and}r\in Z\}$*.*

Since our rings are usually not connected, Lemma 3.2 can be applied only partially.

Example 3.3 The generators in the ring given in Example 2.20 can be reordered to Q(k)[x]〈t〉. Since Q(k)[x] has the idempotent elements $e_{1}$, $e_{2}$, it is not connected. Therefore we get relations such as $(e_{1}+e_{2}\;t)(e_{1}+\frac{e_{2}}{t})=1$ which are predicted in Karpilovsky (1983), Neher (2009).

Subsequently, we enumerate further definitions and properties in difference rings and fields that will be used throughout the article. Let (A,σ) be a difference ring. The rising factorial (or *σ*-factorial) of $f\in A^{⁎}$ to k∈Z is defined by

$$f_{\left(k,\sigma \right)}=\left\{ \begin{array}{cc} f\;\sigma (f)\ldots \;\sigma^{k-1}(f) & \text{if }k>0\\ 1 & \text{if }k=0\\ \sigma^{-1}(f^{-1})\;\sigma^{-2}(f^{-1})\ldots \sigma^{k}(f^{-1}) & \text{if }k<0. \end{array}\right.$$

If the automorphism is clear from the context, we also will write $f_{\left(k\right)}$ instead of $f_{\left(k,\sigma \right)}$. We will rely on the following simple identities (compare also Karr, 1985, p. 307). The proofs are omitted to the reader.

Lemma 3.4 *Let* (A,σ) *be a difference ring,* $f,h\in A^{⁎}$ *and* n,m∈Z*. Then:*

(1) ${\left(f\;h\right)}_{\left(n\right)}=f_{\left(n\right)}\;h_{\left(n\right)}$*.*
(2) $f_{\left(n+m\right)}=\sigma^{n}(f_{\left(m\right)})\;f_{\left(n\right)}$*.*
(3) $f_{\left(n\;m\right)}={\left(f_{\left(n,\sigma \right)}\right)}_{\left(m,\sigma^{n}\right)}$*.*
(4) *If* σ(h)=fh*, then* $\sigma^{n}(h)=f_{\left(n\right)}\;h$*.*
(5) $\sigma^{k}(f)\in A^{⁎}$ *and* $f_{\left(n\right)}\in A^{⁎}$*.*

Let A〈t〉 be a ring of (Laurent) polynomials. For $f=\sum_{i}f_{i}\;t^{i}\in A\langle t\rangle$ we define

$$\deg(f)=\left\{ \begin{array}{cc} \max\{i|f_{i}\neq 0\} & \text{if }f\neq 0\\ -\infty & \text{if }f=0 \end{array}\right.\;\text{ and }\;\mathrm{ldeg}(f)=\left\{ \begin{array}{cc} \min\{i|f_{i}\neq 0\} & \text{if }f\neq 0\\ \infty & \text{if }f=0. \end{array}\right.$$

In addition, for a,b∈Z we introduce the set of truncated (Laurent) polynomials by

(18) $$A{\langle t\rangle }_{a,b}=\{\sum_{i=a}^{b}f_{i}\;t^{i}|f_{i}\in A\}.$$

We conclude this part with the following two lemmas.

Lemma 3.5 *Let* (A〈t〉,σ) *be a unimonomial ring extension of* (A,σ) *of (Laurent) polynomial type. Then for any* k∈Z *and* f∈A〈t〉 *we have that* $\deg(\sigma^{k}(f))=\deg(f)$*.*

Proof Let $f=\sum_{i}f_{i}\;t_{i}$. If f=0, $\sigma^{k}(f)=0$ and thus with deg⁡(0)=−∞ the statement holds. Otherwise, let m:=deg⁡(f)∈Z. Then note that $\sigma^{k}(f)=\sum_{i}\sigma^{k}(f_{i}){\left(\sigma^{k}(t)\right)}^{i}$, i.e., $t^{m}$ is the largest possible monomial in $\sigma^{k}(f)$ with the coefficient $h:=\alpha_{\left(k\right)}^{m}\;\sigma^{k}(f_{m})$. Since $\sigma^{k}(f_{m})\neq 0$ and $\alpha_{\left(k\right)}\in A^{⁎}$ by Lemma 3.4.(5), the coefficient *h* is non-zero.  □

Lemma 3.6 *Let* (F(t),σ) *be a unimonomial field extension of* (F,σ)*, and let* $p,q\in F{\left[t\right]}^{⁎}$ *with* gcd⁡(p,q)=1 *and* k∈Z*. Then the following holds.*

(1) *If* p|q *then* $\sigma^{k}(p)|\sigma^{k}(q)$*.*
(2) $\gcd(\sigma^{k}(p),\sigma^{k}(q))=1$*.*
(3) $\frac{\sigma (p/q)}{p/q}\in F$ *if and only if* σ(p)/p∈F *and* σ(q)/q∈F*.*

Proof (1) If p|q, i.e., pw=q for some w∈F[t]∖{0}, then $\sigma^{k}(p)=\sigma^{k}(w)\;\sigma^{k}(q)$, and thus $\sigma^{k}(p)|\sigma^{k}(q)$. (2) Suppose that $1\neq \gcd(\sigma^{k}(p),\sigma^{k}(q))=:u\in F[t]\setminus F$. Then $\sigma^{-k}(u)\in F[t]\setminus F$. Since $\sigma^{-k}(u)|p$ and $\sigma^{-k}(u)|q$ by part 1 of the lemma, gcd⁡(p,q)≠1, a contradiction to the assumption. (3) The implication ⇐ is immediate. Suppose that u:=σ(p/q)/(p/q)∈F, i.e., σ(p)q=upσ(q). By part 2 of the lemma, σ(p)|p and p|σ(p) which implies that σ(p)/p∈F. Analogously, it follows that σ(q)/q∈F.  □

### 3.1 $\Sigma^{⁎}$-extensions

The essence of all the properties of $\Sigma^{⁎}$-extensions is contained in the following lemma.

Lemma 3.7 *Let* (A,σ) *be a difference ring and let* $G\leq A^{⁎}$ *with* ${\mathrm{sconst}}_{G}\;A\setminus \{0\}\leq A^{⁎}$*. Let* (A[t],σ) *be a unimonomial ring extension of* (A,σ) *with* σ(t)=t+β *for some* β∈A*. If there are a* u∈G *and a* g∈A[t] *with* deg⁡(g)≥1 *such that*

(19) deg⁡(σ(g)−ug)<deg⁡(g)−1

*holds, then there is a* γ∈A *with* σ(γ)−γ=β*.*

Proof Let $g=\sum_{i=0}^{n}g_{i}t^{i}\in A[t]$ with deg⁡(g)=n≥1 and u∈G as stated in the lemma, and define f=σ(g)−ug∈A[t]. With (19) it follows that $f=\sum_{i=0}^{n-2}f_{i}\;t^{i}$. Thus comparing the nth and (n−1)th coefficients in $\sum_{i=0}^{n-2}f_{i}\;t^{i}=f=\sigma (g)-ug=\sum_{i=0}^{n}\sigma (g_{i}){\left(t+\beta \right)}^{i}-u\sum_{i=0}^{n}g_{i}t^{i}$ and using ${\left(t+\beta \right)}^{i}=\sum_{j=0}^{i}\left(\begin{array}{c} i\\ j \end{array}\right)t^{i-j}\beta^{j}$ for 0≤i≤n yield

$$\sigma (g_{n})-ug_{n}=0\;\text{ and }\;\sigma (g_{n-1})+\sigma (g_{n})\left(\begin{array}{c} n\\ 1 \end{array}\right)\beta -ug_{n-1}=0.$$

The first equation shows that $g_{n}\in {\mathrm{sconst}}_{G}\;A\setminus \{0\}\leq A^{⁎}$. Hence we get $u=\sigma (g_{n})/g_{n}$. Substituting *u* for $\sigma (g_{n})/g_{n}$ in the second equation gives $\sigma (g_{n-1})-\frac{\sigma (g_{n})}{g_{n}}g_{n-1}=-n\beta \sigma (g_{n})$. Dividing this equation by $-n\;\sigma (g_{n})\in A^{⁎}$ yields σ(γ)−γ=β with $\gamma :=\frac{-g_{n-1}}{ng_{n}}\in A$.  □

Lemma 3.7 leads to the following equivalent properties of $\Sigma^{⁎}$-extensions.

Lemma 3.8 *Let* (A,σ) *be a difference ring and let* $G\leq A^{⁎}$ *with* ${\mathrm{sconst}}_{G}\;A\setminus \{0\}\leq A^{⁎}$*. Let* (A[t],σ) *be a unimonomial ring extension of* (A,σ) *with* σ(t)=t+β *for some* β∈A*. Then the following statements are equivalent.*

(1) *There are a* g∈A[t]∖A *and* u∈G *with* σ(g)=ug*.*
(2) *There is a* g∈A *with* σ(g)=g+β*.*
(3) constA[t]⊋constA*.*

Proof (1)⇒(2): Let g∈A[t]∖A, u∈G with σ(g)=ug. Since deg⁡(g)≥1 and deg⁡(σ(g)−ug)<0≤deg⁡(g)−1, there is a γ∈A with σ(γ)=γ+β by Lemma 3.7.

(2)⇒(3): Let g∈A with σ(g)=g+β. Since σ(t)=t+β, it follows that σ(t−g)=(t−g), i.e., t−g∈constA[t]. Since t−g∉A, t−g∉constA.

(3)⇒(1): Suppose that constA⊊constA[t] and take g∈constA[t]∖constA. Then σ(g)=ug with u=1∈G. Thus the lemma is proven.  □

As a consequence we can now establish the characterization theorem of $\Sigma^{⁎}$-extensions.

Proof 3.9 **Theorem 2.12.(1)**

For G={1} we have that ${\mathrm{sconst}}_{G}\;A=\mathrm{const}\;A=K$. By assumption K is a field and thus ${\mathrm{sconst}}_{G}\;A\setminus \{0\}\leq A^{⁎}$. Therefore we can apply Lemma 3.8 and its equivalence (2)⇔(3) establishes Theorem 2.12.(1). □

In order to rediscover the difference field version from Karr (1981, 1985), we specialize Lemma 3.8 to difference fields by exploiting Lemma 3.6.(3).

Lemma 3.10 *Let* (F(t),σ) *be a unimonomial field extension of* (F,σ) *with* σ(t)=t+β *for some* β∈F*. Then the following statements are equivalent.*

(1) *There is a* g∈F(t)∖F *with* $\frac{\sigma (g)}{g}\in F$*.*
(2) *There is a* g∈F *with* σ(g)=g+β*.*
(3) constF(t)⊋constF*.*

Proof (1)⇒(2): Let g∈F(t)∖F with σ(g)/g∈F. Write $g=\frac{p}{q}$ with $p,q\in F{\left[t\right]}^{⁎}$ and gcd⁡(p,q)=1. By Lemma 3.6, σ(p)/p∈F and σ(q)/q∈F. Since g∉F, we have that p∉F or q∉F. Thus there is a $g^{\prime }\in F[t]$ with $\deg(g^{\prime })\geq 1$ and $\deg(\sigma (g^{\prime })-g^{\prime })=\deg(0)=-\infty <0\leq \deg(g^{\prime })-1$. Hence by Lemma 3.7 there is a γ∈F with σ(γ)=γ+β.

(2)⇒(3) follows by Lemma 3.8. (3)⇒(1) is analogous to the proof of Lemma 3.8.  □

Note that the above lemma is contained in Karr's work by combining Theorems 2.1 and 2.3 from Karr (1985). As a consequence, we obtain the following result.

Theorem 3.11 *Let* (F(t),σ) *be a unimonomial field extension of* (F,σ) *with* σ(t)=t+β *for some* β∈F*. Then this is a* $\Sigma^{⁎}$*-extension iff there is no* g∈F *with* σ(g)=g+β*.*

By the equivalence (3) ⇔ (1) of Lemma 3.8 we obtain the following result concerning the semi-constants.

Theorem 3.12 *Let* (A,σ) *be a difference ring and let* $G\leq A^{⁎}$ *with* ${\mathrm{sconst}}_{G}\;A\setminus \{0\}\leq A^{⁎}$*. If* (A[t],σ) *is a* $\Sigma^{⁎}$*-extension of* (A,σ)*, then* ${\mathrm{sconst}}_{G}\;A[t]={\mathrm{sconst}}_{G}\;A$*.*

Furthermore, if we specialize to $G=A{\left[t\right]}^{⁎}$ and assume that A is reduced, we get Theorem 3.14. For its proof given below we use in addition the following lemma.

Lemma 3.13 *Let* (A,σ) *be a difference ring:* $\mathrm{sconst}\;A\setminus \{0\}\leq A^{⁎}$ *iff* $\mathrm{sconst}\;A\setminus \{0\}=A^{⁎}$*.*

Proof Suppose that $\mathrm{sconst}\;A\setminus \{0\}\leq A^{⁎}$. If $a\in A^{⁎}$, then $\sigma (a)\in A^{⁎}$. Thus $u:=\frac{\sigma (a)}{a}\in A^{⁎}$. With σ(a)=ua it follows that a∈sconstA∖{0}. Hence $\mathrm{sconst}\;A\setminus \{0\}⊇A^{⁎}$ and with $\mathrm{sconst}\;A\setminus \{0\}\leq A^{⁎}$ we have $\mathrm{sconst}\;A\setminus \{0\}\subseteq A^{⁎}$. The other implication is immediate.  □

Theorem 3.14 *Let* (A[t],σ) *be a* $\Sigma^{⁎}$*-extension of* (A,σ) *where* A *is reduced and* $\mathrm{sconst}\;A\setminus \{0\}\leq A^{⁎}$*. Then* $\mathrm{sconst}\;A[t]\setminus \{0\}=\mathrm{sconst}\;A\setminus \{0\}=A^{⁎}$*.*

Proof By Lemma 3.13 it follows that $\mathrm{sconst}\;A\setminus \{0\}=A^{⁎}$. Since A is reduced, $A{\left[t\right]}^{⁎}=A^{⁎}$ by Lemma 3.2 and thus $\mathrm{sconst}\;A[t]={\mathrm{sconst}}_{A{\left[t\right]}^{⁎}}\;A[t]={\mathrm{sconst}}_{A^{⁎}}\;A[t]$. Now take $G=A^{⁎}$ and apply Theorem 3.12. Hence ${\mathrm{sconst}}_{A^{⁎}}\;A[t]={\mathrm{sconst}}_{A^{⁎}}\;A=\mathrm{sconst}\;A$.  □

### 3.2 Π-extensions

Analogously to Lemma 3.7 we obtain by coefficient comparison the following lemma.

Lemma 3.15 *Let* $\left(A[t,\frac{1}{t}],\sigma \right)$ *be a unimonomial ring extension of* (A,σ) *with* $\alpha =\frac{\sigma (t)}{t}\in A^{⁎}$*; let* u∈A *and* $g=\sum_{i=0}^{n}g_{i}\;t^{i}\in A[t,\frac{1}{t}]$*. If* σ(g)=ug*, then* $\sigma (g_{i})=u\;\alpha^{-i}g_{i}$ *for all i.*

Now we are in the position to obtain the characterization theorem of Π-extensions.

Proof 3.16 **Theorem 2.12.(2)**

“⇐”: Let m∈Z∖{0} and g∈A∖{0} with $\sigma (g)=\alpha^{m}\;g$. Since $\sigma (t^{m})=\alpha^{m}\;t^{m}$, it follows that $\sigma (g/t^{m})=g/t^{m}$, i.e., $g/t^{m}\in \mathrm{const}\;A[t,\frac{1}{t}]$. Clearly $g/t^{m}\notin A$ which implies that $g/t^{m}\notin \mathrm{const}\;A$.

“⇒”: Let $g=\sum_{i}g_{i}t^{i}\in A[t,\frac{1}{t}]\setminus A$ such that σ(g)=g. Thus $g_{m}\neq 0$ for some m≠0. By Lemma 3.15 we have that $\sigma (g_{m})=\alpha^{-m}g_{m}$.

Suppose that *t* is a Π-monomial, but ord(α)=n>0. Then $\sigma (t^{n})=\alpha^{n}\;t^{n}=t^{n}$, which is a contradiction to the first part of the statement. □

Requiring in addition that the semi-constants form a group, this result can be sharpened.

Theorem 3.17 *Let* (A,σ) *be a difference ring and let* $G\leq A^{⁎}$ *with* ${\mathrm{sconst}}_{G}\;A\setminus \{0\}\leq A^{⁎}$*. Let* $\left(A[t,\frac{1}{t}],\sigma \right)$ *be a unimonomial extension of* (A,σ) *with* σ(t)=αt *for some* α∈G*. Then this is a* Π*-extension iff there are no* $g\in {\mathrm{sconst}}_{G}\;A\setminus \{0\}$ *and* m>0 *with* $\sigma (g)=\alpha^{m}\;g$*.*

Proof ⇒: Suppose that *t* is not a Π-monomial. Then we can take g∈A∖{0} and m∈Z∖{0} with $\sigma (g)=\alpha^{m}\;g$. Hence $g\in {\mathrm{sconst}}_{G}\;A\setminus \{0\}\leq A^{⁎}$. Thus if m<0, we get $\sigma (\overset{˜}{g})=\alpha^{-m}\overset{˜}{g}$ with $\overset{˜}{g}=\frac{1}{g}\in A^{⁎}$. The other direction is immediate by Theorem 2.12.(2).  □

Together with Lemma 3.6 we rediscover Karr's field version; see Karr (1985, Theorem 2.2):

Theorem 3.18 *Let* (F(t),σ) *be a unimonomial field extension of* (F,σ) *with* $\alpha =\frac{\sigma (t)}{t}\in F^{⁎}$*. Then this is a* Π*-extension iff there are no* $g\in F^{⁎}$ *and* m>0 *with* $\sigma (g)=\alpha^{m}\;g$*.*

Proof The direction from right to left follows by Theorem 2.12.(2) and the fact that any Π-field extension is a Π-ring extension. Now let g∈F(t)∖F with σ(g)=g. Write g=p/q with p,q∈F(t) where gcd⁡(p,q)=1 and *q* is monic. W.l.o.g. suppose that deg⁡(q)≥deg⁡(p) (otherwise take 1/g instead of *g*). By Lemma 3.6,

(20) σ(p)/p∈Fandσ(q)/q∈F.

We consider two cases. First suppose that $p\in F^{⁎}$ and $q=t^{m}$ with m>0. Then $\frac{p}{t^{m}}=g=\sigma (g)=\frac{\sigma (p)}{\alpha^{m}t^{m}}$ which implies that $\sigma (p)=\alpha^{m}p$. What remains to consider is the case that p∉F or $q\neq t^{m}$ for some m>0. Define

$$a:=\left\{ \begin{array}{cc} p & \text{if }q=t^{m}\text{ for some }m>0,\\ q & \text{otherwise}. \end{array}\right.$$

The following holds.

(1) a∈F[t]∖F: If a=q, note that q∉F by deg⁡(p)≤deg⁡(q) and p/q∉F; if a=p, $q=t^{m}$ and hence p∉F by assumption.
(2) $u:=\sigma (a)/a\in F^{⁎}$ by (20).
(3) $a\neq ut^{m}$ for all $u\in F^{⁎}$ and m>0: *a* could be only of this form, if $q=t^{m}$ for some m>0. Hence a=p. But since gcd⁡(p,q)=1, t∤p.

By the properties (1) and (3), it follows that $a=\sum_{i=k}^{n}a_{i}t^{i}$ with $a_{k}\neq 0\neq a_{n}$ where n>k≥0. Property (2) and Lemma 3.15 yield $\sigma (a_{k})=\frac{u}{\alpha^{k}}a_{k}$ and $\sigma (a_{n})=\frac{u}{\alpha^{n}}a_{n}$ which implies $\sigma (\frac{a_{k}}{a_{n}})=\alpha^{n-k}\frac{a_{k}}{a_{n}}$. Since $\frac{a_{k}}{a_{n}}\in F^{⁎}$ and n−k>0, the theorem is proven.  □

Finally, we characterize the set of semi-constants for Π-extensions.

Proposition 3.19 *Let* (A,σ) *be a difference ring with* $G\leq A^{⁎}$ *and* ${\mathrm{sconst}}_{G}\;A\setminus \{0\}\leq A^{⁎}$*. Let* $\left(A[t,\frac{1}{t}],\sigma \right)$ *be* Π*-extension of* (A,σ) *with* σ(t)=αt *for some* α∈G*. Then* ${\mathrm{sconst}}_{G}\;A[t,\frac{1}{t}]=\{h\;t^{m}|h\in {\mathrm{sconst}}_{G}\;A\text{and}m\in Z\}$ *and* ${\mathrm{sconst}}_{G}\;A[t,\frac{1}{t}]\setminus \{0\}\leq A{\left[t,\frac{1}{t}\right]}^{⁎}$*.*

Proof “⊆”: Let $g\in {\mathrm{sconst}}_{G}\;A[t,\frac{1}{t}]$, i.e., $g=\sum_{i}g_{i}t^{i}\in A[t,\frac{1}{t}]$ with σ(g)=ug for some u∈G. By Lemma 3.15 we get $\sigma (g_{i})\alpha^{i}=u\;g_{i}$ and thus $\sigma (g_{i})=\frac{u}{\alpha^{i}}\;g_{i}$. Now suppose that there are r,s∈Z with s>r and $g_{r}\neq 0\neq g_{s}$. As $\frac{u}{\alpha^{s}}\in G$, it follows that $g_{s}\in {\mathrm{sconst}}_{G}\;A\setminus \{0\}\leq A^{⁎}$. Thus we conclude that $\sigma (\frac{g_{r}}{g_{s}})=\alpha^{s-r}\frac{g_{r}}{g_{s}}$ with s−r>0; a contradiction to Theorem 2.12.(2). Hence $g=h\;t^{m}$ for some $h\in {\mathrm{sconst}}_{G}\;A$, m∈Z.

“⊇”: Let $g=h\;t^{m}$ with $h\in {\mathrm{sconst}}_{G}\;A$, m∈Z. Then there is a u∈G with σ(h)=uh. Hence $\sigma (g)=\sigma (h)\;\alpha^{m}t^{m}=u\;\alpha^{m}h\;t^{m}=u\;\alpha^{m}\;g$ with $u\;\alpha^{m}\in G$. Thus $g\in {\mathrm{sconst}}_{G}\;A[t,\frac{1}{t}]$.

Summarizing, we proved equality which implies that ${\mathrm{sconst}}_{G}\;A[t,\frac{1}{t}]\setminus \{0\}\leq A{\left[t,\frac{1}{t}\right]}^{⁎}$.  □

So far we obtained a description of the semi-constants for a subgroup *G* of $A^{⁎}$. Now we will lift this result to the group

$$\overset{˜}{G}=G_{A}^{A\langle t\rangle }=\{ht^{m}|\;h\in G\text{ and }m\in Z\}\leq A{\langle t\rangle }^{⁎}.$$

Theorem 3.20 *Let* (A,σ) *be a difference ring and let* $G\leq A^{⁎}$ *with* ${\mathrm{sconst}}_{G}\;A\setminus \{0\}\leq A^{⁎}$*. Let* $\left(A[t,\frac{1}{t}],\sigma \right)$ *be* Π*-extension of* (A,σ) *with* σ(t)=αt *for some* α∈G *and let* $\overset{˜}{G}=G_{A}^{A\langle t\rangle }$*. Then* ${\mathrm{sconst}}_{\overset{˜}{G}}\;A[t,\frac{1}{t}]={\mathrm{sconst}}_{G}\;A[t,\frac{1}{t}]=\{h\;t^{m}|h\in {\mathrm{sconst}}_{G}\;A\text{and}m\in Z\}$*.*

Proof We show that ${\mathrm{sconst}}_{\overset{˜}{G}}\;A[t,\frac{1}{t}]={\mathrm{sconst}}_{G}\;A[t,\frac{1}{t}]$. Then by Proposition 3.19 the theorem is proven. Since $G\leq \overset{˜}{G}$, the inclusion ${\mathrm{sconst}}_{\overset{˜}{G}}\;A[t,\frac{1}{t}]⊇{\mathrm{sconst}}_{G}\;A[t,\frac{1}{t}]$ is immediate. Now suppose that $g=\sum_{i}g_{i}\;t^{i}\in {\mathrm{sconst}}_{\overset{˜}{G}}\;A[t,\frac{1}{t}]$. Hence there are an m∈Z and an h∈G with $\sigma (g)=h\;t^{m}g$. By coefficient comparison it follows that $\sigma (g_{i})\alpha^{i}=hg_{i-m}$. If m≥1, take *s* minimal such that $g_{s}\neq 0$. Then $\sigma (g_{s})\alpha^{s}\neq 0$. But by the choice of *s*, we get $g_{s-m}=0$ and thus $h\;g_{s-m}=0$, a contradiction. Otherwise, if m<0, take *s* maximal such that $g_{s-m}\neq 0$. Then $h\;g_{s-m}\neq 0$. But by the choice of *s*, we get $\sigma (g_{s})\alpha^{s}=0$, again a contradiction. Thus m=0 and consequently, $g\in {\mathrm{sconst}}_{G}\;A[t,\frac{1}{t}]$.  □

We close this subsection with Theorem 3.21. It provides a description of $\mathrm{sconst}\;A[t,\frac{1}{t}]$ under the assumption that A is reduced and connected. This result is not applicable if general *R*-extensions pop up; see Example 3.3. But, it will be used for further insights summarized in Corollary 4.6.(2), Proposition 4.14 and Corollary 4.15 below.

Theorem 3.21 *Let* (A,σ) *be a difference ring being reduced and connected with* $\mathrm{sconst}\;A\setminus \{0\}=A^{⁎}$*. Let* $\left(A[t,\frac{1}{t}],\sigma \right)$ *be* Π*-extension of* (A,σ) *with* σ(t)=αt *for some* $\alpha \in A^{⁎}$*. Then* $\mathrm{sconst}\;A[t,\frac{1}{t}]=\{h\;t^{m}|\;h\in \mathrm{sconst}\;A\text{and}m\in Z\}$*.*

Proof Take $\overset{˜}{G}={\left(A^{⁎}\right)}_{A}^{A\langle t\rangle }$. Then $\overset{˜}{G}=A{\left[t,\frac{1}{t}\right]}^{⁎}$ by Lemma 3.2. Thus we conclude that $\mathrm{sconst}\;A[t,\frac{1}{t}]={\mathrm{sconst}}_{A{\left[t,1/t\right]}^{⁎}}A[t,\frac{1}{t}]={\mathrm{sconst}}_{\overset{˜}{G}}\;A[t,\frac{1}{t}]\overset{\mathrm{Thm}.\;\text{3.20}}{=}\{h\;t^{m}|\;h\in \mathrm{sconst}\;A\text{ and }m\in Z\}$.  □

### 3.3 *R*-extensions

We start with the proof of the characterization theorem of *R*-extensions.

Proof 3.22 **Theorem 2.12.(3)**

“⇐”: Let m∈{1,…,λ−1} and g∈A∖{0} with $\sigma (g)=\alpha^{m}\;g$. Since $\sigma (t^{m})=\alpha^{m}\;t^{m}$, it follows that $\sigma (g\;t^{\lambda -m})=g\;t^{\lambda -m}$, i.e., $g\;t^{\lambda -m}\in \mathrm{const}\;A[t]$. Clearly $g\;t^{\lambda -m}\notin A$ which implies that $g\;t^{\lambda -m}\notin \mathrm{const}\;A$.

“⇒”: Let $g=\sum_{i=0}^{\lambda -1}g_{i}\;t^{i}\in A[t]\setminus A$ with σ(g)=g. Thus $g_{r}\neq 0$ for some r∈{1,…,λ−1}. By coefficient comparison we get $\sigma (g_{r})=\alpha^{\lambda -r}g_{r}$ with λ−r∈{1,…,λ−1}.

Let *t* be an *R*-monomial and let m:=ord(α)<λ. Then with g=1∈A∖{0} we have that $\sigma (g)=1=\alpha^{m}\;1=\alpha^{m}\;g$. A contradiction to the first statement.  □

Finally, we work out properties for the set of semi-constants. Since the proof of the following theorem is completely analogous to the proof of Proposition 3.19, it is skipped.

Proposition 3.23 *Let* (A,σ) *be a difference ring with* $G\leq A^{⁎}$ *and* ${\mathrm{sconst}}_{G}\;A\setminus \{0\}\leq A^{⁎}$*. Let* (A[x],σ) *be an R-extension of* (A,σ) *with* $\alpha =\frac{\sigma (x)}{x}\in G$ *and* λ:=ord(x)=ord(α)>1*. Then* ${\mathrm{sconst}}_{G}\;A[x]=\{h\;x^{m}|h\in {\mathrm{sconst}}_{G}\;A,\;0\leq m<\lambda \}$ *and* ${\mathrm{sconst}}_{G}\;A[x]\setminus \{0\}\leq A{\left[x\right]}^{⁎}$*.*

As in Theorem 3.20 we will lift this result from the group $G\leq A^{⁎}$ to

$$\overset{˜}{G}=G_{A}^{A[x]}=\{h\;x^{m}|\;h\in G\text{ and }m\in \{0,\ldots ,\lambda -1\}\}\leq A{\left[x\right]}^{⁎}.$$

We remark that there is the following subtlety. We have to assume that A[x] is reduced in order to prove the result below. In order to take care of this extra property, further investigations will be necessary in Subsection 4.1.

Theorem 3.24 *Let* (A[x],σ) *be an R-extension of* (A,σ) *and let* $G\leq A^{⁎}$ *with* ${\mathrm{sconst}}_{G}\;A\setminus \{0\}\leq A^{⁎}$*. If* A[x] *is reduced, then* ${\mathrm{sconst}}_{\overset{˜}{G}}\;A[x]\setminus \{0\}\leq A{\left[x\right]}^{⁎}$ *for* $\overset{˜}{G}=G_{A}^{A[x]}$*.*

Proof Let $\alpha :=\frac{\sigma (x)}{x}\in A^{⁎}$ and n=ord(α)=ord(x). Let $g\in {\mathrm{sconst}}_{\overset{˜}{G}}\;A[x]\setminus \{0\}$, i.e., $\sigma (g)=u\;x^{m}\;g$ with u∈G and 0≤m<n. Since $x^{m\;n}=1$, $\sigma (g^{n})=u^{n}\;g^{n}$ with $u^{n}\in G$.

First suppose that $v:=g^{n}\in A$. Since A[x] is reduced, v≠0 and thus $v\in {\mathrm{sconst}}_{G}\;A\setminus \{0\}\leq A^{⁎}$, i.e., $g\;(g^{n-1}/v)=1$. Hence *g* is invertible, i.e., $g\in A{\left[x\right]}^{⁎}$.

Otherwise, suppose that $v:=g^{n}\notin A$. Define $a:=u^{n}\in G$. We consider two sub-cases. Suppose that there are a k>0 and a w∈A∖{0} with $\sigma (w)=a^{k}\;w$. Then $w\in {\mathrm{sconst}}_{G}\;A\setminus \{0\}\leq A^{⁎}$. Hence $\sigma ({\left(g^{n}\right)}^{k}/w)={\left(g^{n}\right)}^{k}/w$, i.e., $c:={\left(g^{n}\right)}^{k}/w\in K$, and since A[x] is reduced, c≠0. Thus (as above) $g\;(g^{k\;n-1}/w/c)=1$ and therefore $g\in K{\left[x\right]}^{⁎}$.

Finally, suppose that there are no k>0 and w∈A∖{0} with $\sigma (w)=a^{k}\;g$. Hence by Theorem 3.17 there is the Π-extension $\left(A[t,\frac{1}{t}],\sigma \right)$ of (A,σ) with σ(t)=at ($a\in G\leq A^{⁎}$). Let $v=g^{n}=\sum_{i=0}^{n-1}v_{i}\;x^{i}\in A[x]\setminus A$. Then σ(v)=av and thus by coefficient comparison it follows that $\sigma (v_{i})=a\;\alpha^{n-i}\;v_{i}$ for some $v_{i}\in A\setminus \{0\}$ with 1≤i<n. Hence $\sigma (\frac{v_{i}}{t})=\alpha^{n-i}\frac{v_{i}}{t}$, and thus $\sigma (\frac{v_{i}^{n}}{t^{n}})=\frac{v_{i}^{n}}{t^{n}}$. Since A is reduced, we have $v_{i}^{n}\neq 0$, and consequently $\frac{v_{i}^{n}}{t^{n}}\in \mathrm{const}\;A[t,\frac{1}{t}]\setminus A$, a contradiction that *t* is a Π-monomial. Thus this case can be excluded. Summarizing, any element in ${\mathrm{sconst}}_{\overset{˜}{G}}\;A[x]\setminus \{0\}$ is from $A{\left[x\right]}^{⁎}$.  □

## 4 Nested $R\Pi \Sigma^{⁎}$-extensions and simple $R\Pi \Sigma^{⁎}$-extensions

We explore the set of semi-constants. First we deal with nested *R*-extensions in Subsection 4.1 and with nested $\Pi \Sigma^{⁎}$-extensions in Subsection 4.2. Finally, we obtain Theorems 2.22 and 2.24 for nested $R\Pi \Sigma^{⁎}$-extensions in Subsection 4.3. In addition, we work out further structural properties of (simple) $R\Pi \Sigma^{⁎}$-extensions.

### 4.1 Nested *R*-extensions

We derive a first result of the semi-constants by applying iteratively Proposition 3.23.

Proposition 4.1 *Let* (A,σ) *be a difference ring with* $G\leq A^{⁎}$ *and* ${\mathrm{sconst}}_{G}\;A\setminus \{0\}\leq A^{⁎}$*. Let* (E,σ) *with* $E=A\langle x_{1}\rangle \ldots \langle x_{e}\rangle$ *be an R-extension of* (A,σ) *with* $\frac{\sigma (x_{i})}{x_{i}}\in G$ *and* $n_{i}=\mathrm{ord}(x_{i})$*. Then* ${\mathrm{sconst}}_{G}\;E=\{h\;x_{1}^{m_{1}}\ldots x_{1}^{m_{e}}|\;h\in {\mathrm{sconst}}_{G}\;A\text{and}0\leq m_{i}<n_{i}\text{for}1\leq i\leq e\}$ *and* ${\mathrm{sconst}}_{G}\;E\setminus \{0\}\leq E^{⁎}$*.*

In order to treat nested *R*-extensions, we proceed as follows. Let $\left(A\langle x_{1}\rangle \ldots \langle x_{e}\rangle ,\sigma \right)$ be an *R*-extension of (A,σ) with $\lambda_{i}=\mathrm{ord}(x_{i})$ and $\sigma (x_{i})=\alpha_{i}\;x_{i}$. Moreover, take the polynomial ring $R=A[y_{1},\ldots ,y_{e}]$ and define $\alpha_{i}^{\prime }=\alpha {|}_{x_{1}\to y_{1},\ldots ,x_{i-1}\to y_{i-1}}$. Then we obtain the automorphism $\sigma^{\prime }:R\to R$ by $\sigma^{\prime }{|}_{A}=\sigma$ and $\sigma (y_{i})=\alpha_{i}y_{i}$, i.e., $\left(R,\sigma^{\prime }\right)$ is a difference ring extension of (A,σ). Thus by iterative application of the construction used for Lemma 2.6 it follows that $A\langle x_{1}\rangle \ldots \langle x_{e}\rangle$ is isomorphic to R/I where *I* is the ideal

(21) $$I=\langle y_{1}^{\lambda_{1}}-1,\ldots ,y_{e}^{\lambda_{e}}-1\rangle$$

in *R*. In particular, we obtain the automorphism $\sigma^{\prime \prime }:R/I\to R/I$ defined by $\sigma^{\prime \prime }(f+I)=\sigma^{\prime }(f)+I$ and it follows that the difference ring $\left(A\langle x_{1}\rangle \ldots \langle x_{e}\rangle ,\sigma \right)$ is isomorphic to $\left(R/I,\sigma^{\prime \prime }\right)$; here $f\in A\langle x_{1}\rangle \ldots \langle x_{e}\rangle$ is mapped to $f^{\prime }+I$ with $f^{\prime }=f{|}_{x_{1}\to y_{1},\ldots ,x_{e}\to y_{e}}$.

Take $G={\left(F^{⁎}\right)}_{F}^{E}\leq E^{⁎}$. In order to show that ${\mathrm{sconst}}_{G}\;E\setminus \{0\}\leq E^{⁎}$ holds as claimed in Corollary 4.3 below, we use Gröbner bases theory.

Lemma 4.2 *Let* $\lambda_{i}\in N\setminus \{0\}$*. Then the zero-dimensional ideal I given in* (21) *in the polynomial ring* $R=F[y_{1},\ldots ,y_{e}]$ *is radical.*

Proof The ideal *I* is zero-dimensional. Since F has characteristic 0, it is perfect. We therefore apply Seidenberg's criterion (algorithm) given in Becker and Weispfenning (1993, Thm. 8.22). Define $f_{i}=y_{i}^{\lambda_{i}}-1$. Then for each *i* (1≤i≤e) we have that $f_{i}\in R\cap F[y_{i}]$ and $\gcd(f_{i},\frac{d}{dy_{i}}f_{i})=\gcd(y_{i}^{\lambda_{i}}-1,\lambda_{i}y^{\lambda_{i}-1})=1$. Thus Becker and Weispfenning (1993, Thm. 8.22) imply that $\langle f_{1},\ldots ,f_{e}\rangle$ is radical.  □

Corollary 4.3 *Let* (E,σ) *be an R-extension of a difference field* (F,σ) *and let* $G={\left(F^{⁎}\right)}_{F}^{E}$*. Then* E *is reduced and* ${\mathrm{sconst}}_{G}\;E\setminus \{0\}\leq E^{⁎}$*.*

Proof The difference ring (E,σ) with $E=F\langle x_{1}\rangle \ldots \langle x_{r}\rangle$ is isomorphic to $\left(R/I,\sigma^{\prime \prime }\right)$ as defined above with (21) where A=F. Suppose that E is not reduced. Then there are an f∈E∖{0} and an n>0 with $f^{n}=0$. Hence there is an h∈R with h+I≠I and ${\left(h+I\right)}^{n}=h^{n}+I=I$. This implies that h∉I and $h^{n}\in I$. Therefore *I* is not radical, a contradiction to Lemma 4.2. Hence E is reduced. Thus we can apply Theorem 3.24 iteratively and it follows that ${\mathrm{sconst}}_{G}\;E\setminus \{0\}\leq E^{⁎}$.  □

### 4.2 Nested $\Pi \Sigma^{⁎}$-extensions

In Corollary 4.6 we will characterize the set of semi-constants within $\Pi \Sigma^{⁎}$-extensions. Part 1 will deal with the general case. Part 2 assumes in addition that the ground ring is reduced and connected. In this setting, we rely on the following two lemmas.

Lemma 4.4 *Let* (A〈t〉,σ) *be a* $\Pi \Sigma^{⁎}$*-extension of* (A,σ)*. If* A *is reduced,* A〈t〉 *is reduced. If* A *is reduced and connected,* A〈t〉 *is reduced and connected.*

Proof Let *t* be a Π-monomial. Moreover, let A be reduced. Now take $f=\sum_{i}f_{i}t^{i}\in A\langle t\rangle =A[t,\frac{1}{t}]$ with f≠0 and $f^{n}=0$ for some n>0. Since A is reduced, f∉A. Let m∈Z be maximal such that $f_{m}\neq 0$. Then the coefficient of $t^{n\;m}$ in $f^{n}$ is $f_{m}^{n}$. Hence $f_{m}^{n}=0$ and thus $f_{m}$ is a nilpotent element in A, a contradiction.

Now let A be reduced and connected and take $f=\sum_{i}f_{i}t^{i}\in A\langle t\rangle =A[t,\frac{1}{t}]$ with $f^{2}=f$ and f∉{0,1}. Since A is connected, f∉A. Let *m* be maximal such that $f_{m}\neq 0$. If m>0, then the coefficient of $t^{2m}$ in $f^{2}$ is $f_{m}^{2}$ and thus with $f^{2}=f$ we have that $f_{m}^{2}=0$; a contradiction that A is reduced. Otherwise, if m=0, we take $\overset{¯}{m}$ minimal with $f_{\overset{¯}{m}}\neq 0$. Note that $\overset{¯}{m}<0$ since f∉A. As above, it follows that $f_{\overset{¯}{m}}^{2}=0$, again a contradiction. Summarizing, if A is reduced (and connected), $A[t,\frac{1}{t}]$ is reduced (and connected). For a $\Sigma^{⁎}$-monomial *t*, the same implications hold since $A\langle t\rangle =A[t]\leq A[t,\frac{1}{t}]$.  □

If A is reduced, the shift behavior of Π-monomials does not depend on $\Sigma^{⁎}$-monomials.

Lemma 4.5 *Let* (E,σ) *be a* $\Pi \Sigma^{⁎}$*-ring extension of* (A,σ) *where* A *is reduced. Then the generators can be reordered such that we get the form* $E=A\langle t_{1}\rangle \ldots \langle t_{p}\rangle \langle s_{1}\rangle \ldots \langle s_{e}\rangle$ *where the* $t_{i}$ *are* Π*-monomials and the* $s_{i}$ *are* $\Sigma^{⁎}$*-monomials.*

Proof Let $E=A\langle t_{1}\rangle \ldots \langle t_{e}\rangle$. By iterative application of Lemma 4.4 it follows that E is reduced. Let $t_{i}$ be a Π-monomial where $\alpha =\sigma (t_{i})/t_{i}\in A\langle t_{1}\rangle \ldots \langle t_{i-1}\rangle$ depends on a $\Sigma^{⁎}$-monomial $t_{j}$ with j<i. Then we can reorder the generators such that we get $H=A\langle t_{1}\rangle \ldots \langle t_{j-1}\rangle \langle t_{j+1}\rangle \ldots \langle t_{i-1}\rangle$; here we forget *σ* and argue purely in the given ring. In particular, $\alpha \in H\langle t_{j}\rangle =H[t_{j}]\setminus H$. Since *α* is invertible, α∈H by Lemma 3.2; a contradiction. Summarizing, for all Π-monomials $t_{j}$ we have that $\sigma (t_{j})/t_{j}$ is free of $\Sigma^{⁎}$-monomials. Thus we can shuffle all Π-monomials to the left and all $\Sigma^{⁎}$-monomials to the right and obtain again a $\Pi \Sigma^{⁎}$-extension.  □

Corollary 4.6 *Let* (E,σ) *be a* $\Pi \Sigma^{⁎}$*-extension of* (A,σ) *with* $E=A\langle t_{1}\rangle \langle t_{2}\rangle \ldots \langle t_{e}\rangle$*.*

(1) *Let* $G\leq A^{⁎}$ *with* ${\mathrm{sconst}}_{G}\;A\setminus \{0\}\leq A^{⁎}$ *and let* $\overset{˜}{G}=G_{A}^{E}$*. If* (E,σ) *is a G-simple* $\Pi \Sigma^{⁎}$*-extension of* (A,σ)*, then* ${\mathrm{sconst}}_{\overset{˜}{G}}\;E\setminus \{0\}\leq E^{⁎}$ *where*
  $${\mathrm{sconst}}_{\overset{˜}{G}}\;E=\{h\;t_{1}^{m_{1}}\ldots t_{e}^{m_{e}}|\;h\in {\mathrm{sconst}}_{G}\;A\text{and}m_{i}\in Z\text{where}m_{i}=0\text{if}t_{i}\text{is a}\Sigma^{⁎}\text{-monomial}\}.$$
(2) *If* A *is reduced and connected and* $\mathrm{sconst}\;A\setminus \{0\}=A^{⁎}$*, then*
  (22) $$\mathrm{sconst}\;E\setminus \{0\}=\{h\;t_{1}^{m_{1}}\ldots t_{e}^{m_{e}}|\;h\in A^{⁎}\text{and}m_{i}\in Z\;\text{where}m_{i}=0\text{if}t_{i}\text{is a}\Sigma^{⁎}\text{-monomial}\}=E^{⁎}.$$
(3) *If* A *is a field then we have that* (22)*.*

Proof The first part is proven by induction on the number *e* of extensions. If e=0, nothing has to be shown. Now suppose that the first part holds and consider one extra $\overset{˜}{G}$-simple $\Pi \Sigma^{⁎}$-monomial $t_{e+1}$ on top. Define $\overset{˜}{\overset{˜}{G}}={\overset{˜}{G}}_{E}^{E\langle t_{e+1}\rangle }=G_{A}^{E\langle t_{e+1}\rangle }$. If $t_{i}$ is a $\Sigma^{⁎}$-monomial, $\overset{˜}{\overset{˜}{G}}=\overset{˜}{G}$. Together with Theorem 3.12 it follows that ${\mathrm{sconst}}_{\overset{˜}{\overset{˜}{G}}}\;E[t_{e+1}]={\mathrm{sconst}}_{\overset{˜}{G}}\;E[t_{e+1}]={\mathrm{sconst}}_{\overset{˜}{G}}\;E$ and ${\mathrm{sconst}}_{\overset{˜}{\overset{˜}{G}}}\;E[t_{e+1}]\setminus \{0\}\leq E^{⁎}\leq E{\left[t_{e+1}\right]}^{⁎}$. If $t_{i}$ is a Π-monomial, we have $\sigma (t_{e+1})/t_{e+1}\in \overset{˜}{G}$. Hence Theorem 3.20 yields ${\mathrm{sconst}}_{\overset{˜}{\overset{˜}{G}}}\;E[t_{e+1},\frac{1}{t_{e+1}}]=\{h\;t_{e+1}^{m}|\;m\in Z\text{ and }h\in {\mathrm{sconst}}_{\overset{˜}{G}}\;E\}$ and thus by the induction assumption we have that

$${\mathrm{sconst}}_{\overset{˜}{\overset{˜}{G}}}\;E[t_{e+1},\frac{1}{t_{e+1}}]=\{h\;t_{1}^{m_{1}}\ldots t_{e+1}^{m_{e+1}}|\;h\in {\mathrm{sconst}}_{G}\;A\text{ and }m_{i}\in Z\;\text{where }m_{i}=0\text{ if }t_{i}\text{ is a }\Sigma^{⁎}\text{-monomial}\}$$

and thus ${\mathrm{sconst}}_{\overset{˜}{\overset{˜}{G}}}\;E[t_{e+1},\frac{1}{t_{e+1}}]\setminus \{0\}\leq E{\left[t_{e+1},\frac{1}{t_{e+1}}\right]}^{⁎}$. This completes the induction step.

Similarly, the first equality of part 2 follows by Theorems 3.14 and 3.21. The second equality follows by Lemmas 3.2 and 4.4. Since any field is connected and reduced and $\mathrm{sconst}\;A\setminus \{0\}=A^{⁎}$ by Lemma 3.13, part 3 follows by part 2.  □

Restricting to $\Sigma^{⁎}$-extensions, the above result simplifies as follows.

Corollary 4.7 *Let* (E,σ) *be a* $\Sigma^{⁎}$*-extension of* (A,σ)*. Then the following holds.*

(1) *If* $G\leq A^{⁎}$ *with* ${\mathrm{sconst}}_{G}\;A\setminus \{0\}\leq A^{⁎}$*, then* ${\mathrm{sconst}}_{G}\;E={\mathrm{sconst}}_{G}\;A$*.*
(2) *If* A *is reduced and* $\mathrm{sconst}\;A\setminus \{0\}=A^{⁎}$*, then* E *is reduced and* sconstE=sconstA*.*
(3) *If* A *is a field, then* $\mathrm{sconst}\;E\setminus \{0\}=A^{⁎}=A\setminus \{0\}$*.*

### 4.3 $R\Pi \Sigma^{⁎}$-extensions and their simple and single-rooted restrictions

We turn to the set of semi-constants within nested $R\Pi \Sigma^{⁎}$-extensions. The case of simple and single-rooted $R\Pi \Sigma^{⁎}$-extensions is immediate.

Proof 4.8 **Theorem 2.22**

This follows by Corollary 4.6.(1) and Proposition 4.1. □

Likewise, simple $R\Pi \Sigma^{⁎}$-extension can be treated if they are built in a particular form.

Theorem 4.9 *Let* (H,σ) *be an R-extension of a difference field* (F,σ) *and let* (E,σ) *with* $E=H\langle t_{1}\rangle \langle t_{2}\rangle \ldots \langle t_{e}\rangle$ *be a simple* $\Pi \Sigma^{⁎}$*-extension of* (H,σ)*. Let* $G={\left(F^{⁎}\right)}_{F}^{H}$ *and define* $\overset{˜}{G}=G_{H}^{E}$*. Then we have* ${\mathrm{sconst}}_{\overset{˜}{G}}\;E\setminus \{0\}\leq E^{⁎}$ *where*

$${\mathrm{sconst}}_{\overset{˜}{G}}\;E=\{h\;t_{1}^{m_{1}}\ldots t_{e}^{m_{e}}|\;h\in {\mathrm{sconst}}_{G}\;H,\;m_{i}\in Z\text{where}m_{i}=0\text{if}t_{i}\text{is a}\Sigma^{⁎}\text{-monomial}\}.$$

Proof By Corollary 4.3, ${\mathrm{sconst}}_{G}\;H\setminus \{0\}\leq H^{⁎}$. Hence the result follows by Corollary 4.6.(1).  □

Next, we show that simple $R\Pi \Sigma^{⁎}$-extensions can be always brought to the shape as assumed in Theorem 4.9. This will finally produce a proof of Theorem 2.24.

Lemma 4.10 *Let* (A,σ) *be a difference ring with a group* $G\leq A^{⁎}$ *and let* (E,σ) *be a G-simple* $R\Pi \Sigma^{⁎}$*-extension of* (A,σ)*.*

(1) *The* $R\Pi \Sigma^{⁎}$*-monomials can be reordered to the form* $E=A\langle t_{1}\rangle \langle t_{2}\rangle \ldots \langle t_{e}\rangle$ *with* r,p∈N *(*0≤r≤p≤e*) such that the following holds.*
  • *For all i* (1≤i≤r)*,* $t_{i}$ *is an R-monomial with* $\sigma (t_{i})/t_{i}=u_{i}\;t_{1}^{z_{1}}\ldots t_{i-1}^{z_{i-1}}$ *for some root of unity* $u_{i}\in G$ *and* $z_{i}\in N$*.*
  • *For all i* (r<i≤p)*,* $t_{i}$ *is a* Π*-monomial with* $\sigma (t_{i})/t_{i}=u_{i}\;t_{1}^{z_{1}}\ldots t_{i-1}^{z_{i-1}}$ *for some* $u_{i}\in G$ *and* $z_{i}\in Z$*.*
  • *For all i* (p<i≤e)*,* $t_{i}$ *is a* $\Sigma^{⁎}$*-monomial with* $\sigma (t_{i})-t_{i}\in A\langle t_{1}\rangle \langle t_{2}\rangle \ldots \langle t_{i-1}\rangle$*.*
(2) *For any* $f\in G_{A}^{E}$ *which depends on a* Π*-monomial we have that* ord(f)=0*.*

Proof We show the lemma by induction on the number of $R\Pi \Sigma^{⁎}$-monomials. Suppose that the lemma holds for *e* extensions. Now let $E=A\langle t_{1}\rangle \ldots \langle t_{e}\rangle$ and consider the $R\Pi \Sigma^{⁎}$-monomial $t_{e+1}$ on top of E. By the induction assumption we can reorder E such that it has the desired form (all *R*-monomials are on the left, all Π-monomials are in the middle and all $\Sigma^{⁎}$-monomials are on the right). If $t_{e+1}$ is a $\Sigma^{⁎}$-monomial, the required shape is fulfilled. If $t_{e+1}$ is an *R*-monomial, observe that $\alpha :=\sigma (t_{e+1})/t_{e+1}\in G_{A}^{E}$. Since $\mathrm{ord}(\alpha )=\mathrm{ord}(t_{e+1})>1$ by Theorem 2.12.(3), *α* is free of Π-monomials by the induction assumption and (by definition) free of $\Sigma^{⁎}$-monomials. Thus we can shuffle $t_{e+1}$ to the left (such that all $\Pi \Sigma^{⁎}$-monomials are to the right), and the required shape is satisfied. Similarly, if $t_{e+1}$ is a Π-monomial, $\sigma (t_{e+1})/t_{e+1}\in G_{A}^{E}$ is free of $\Sigma^{⁎}$-monomials by definition and we can shuffle $t_{e+1}$ to the left such that all $\Sigma^{⁎}$-monomials are to the right. This completes the first part of the lemma. Now let $E=A\langle x_{1}\rangle \ldots \langle x_{e+1}\rangle$ be in the desired ordered form. If $x_{e+1}$ is a $\Sigma^{⁎}$-monomial, we have that $G_{A}^{E\langle x_{e+1}\rangle }=G_{A}^{E}$. Thus the second part holds by the induction assumption. If $x_{e+1}$ is an *R*-extension, also all $x_{i}$ with 1≤i≤e are *R*-monomials, and the second statement holds trivially. Finally, let $x_{e+1}$ be a Π-monomial and take $f\in G_{A}^{E\langle x_{e+1}\rangle }$. If f∈E and *f* depends on Π-monomials, we have again that ord(f)=0 by the induction assumption. To this end, suppose that *f* depends on $x_{e+1}$ and we have that ord(f)=n>0. Then $f=u\;x_{e+1}^{m}$ where m≠0 and $u\in E^{⁎}$. Since $f^{n}=1$, $u^{n}\;x_{e+1}^{m\;n}=1$ where $u^{n}\neq 0$. Hence $x_{e+1}$ is not transcendental over E, a contradiction to the definition of a Π-monomial. Thus ord(f)=n=0. This completes the proof.  □

Proof 4.11 **Theorem 2.24**

By Lemma 4.10 we can reorder the simple $R\Pi \Sigma^{⁎}$-extension such that Theorem 4.9 is applicable.  □

In the remaining part of this section we deliver insight into the structure of (simple) $R\Pi \Sigma^{⁎}$-extensions. First observe that a tower of simple $R\Pi \Sigma^{⁎}$-extensions is again simple.

Lemma 4.12 *Let* (A,σ) *be a difference ring with a group* $G\leq A^{⁎}$ *and let* (A,σ)≤(H,σ)≤(E,σ) *be* $R\Pi \Sigma^{⁎}$*-extensions. Then* ${\left(G_{A}^{H}\right)}_{H}^{E}=G_{A}^{E}$*. Moreover, if* (A,σ)≤(H,σ) *is G-simple and* (H,σ)≤(E,σ) *is* $G_{A}^{H}$*-simple, then* (A,σ)≤(E,σ) *is G-simple.*

Further, the reordering as described in Lemma 4.10 is also possible if one relaxes the condition that the $R\Pi \Sigma^{⁎}$-extension is simple but requires that the ground ring is a field.

Lemma 4.13 *Let* (E,σ) *be an* $R\Pi \Sigma^{⁎}$*-ring extension of a difference field* (F,σ)*. Then* (E,σ) *can be reordered to the form* $E=F\langle x_{1}\rangle \ldots \langle x_{r}\rangle \langle t_{1}\rangle \ldots \langle t_{p}\rangle \langle s_{1}\rangle \ldots \langle s_{e}\rangle$ *where the* $x_{i}$ *are R-monomials, the* $t_{i}$ *are* Π*-monomials and the* $s_{i}$ *are* $\Sigma^{⁎}$*-monomials.*

Proof First we try to shuffle all *R*-extensions to the front. Suppose that this fails at the first time. Then there are an *R*-extension (H,σ) of (F,σ), a $\Pi \Sigma^{⁎}$-extension (G,σ) of (H,σ) with $G=H\langle y_{1}\rangle \ldots \langle y_{l}\rangle$ and an *R*-extension (G〈x〉,σ) of (G,σ) with α=σ(x)/x in which $y_{l}$ occurs. Note that H is reduced by Corollary 4.3, and G is reduced by iterative application of Lemma 4.4. Write $\alpha =\sum_{i}f_{i}y_{l}^{i}$. Let m≠0 such that $f_{m}\neq 0$ and such that |m|≥1 is maximal (we remark that m<0 can only happen if $y_{l}$ is a Π-monomial). By the choice of *m*, we have that the coefficient of $y_{l}^{m\;n}$ in $\alpha^{n}$ is $f_{m}^{n}$. Hence with $\alpha^{n}=1$ it follows that $f_{m}^{n}=0$, a contradiction to the assumption that G is reduced. Therefore we can shuffle all *R*-monomials to the left and all $\Pi \Sigma^{⁎}$-monomials to the right. Since the nested *R*-extension is reduced by Corollary 4.3, we can apply Lemma 4.5 to reorder the $\Pi \Sigma^{⁎}$-monomials further as claimed in the statement.  □

By definition any (nested) $\Sigma^{⁎}$-extension is also a simple $\Sigma^{⁎}$-extension. If the ground ring is reduced and connected, we obtain the following stronger result.

Proposition 4.14 *Let* (G,σ) *be a difference ring where* G *is reduced and connected and where* $\mathrm{sconst}\;G\setminus \{0\}=G^{⁎}$*. Then a* $\Pi \Sigma^{⁎}$*-extension* (E,σ) *of* (G,σ) *is simple.*

Proof Let $E=G\langle t_{1}\rangle \ldots \langle t_{e}\rangle$. By Lemma 4.5 we may suppose that the generators are ordered such that the $t_{1}\ldots ,t_{p}$ are Π-monomials and the $t_{p+1}\ldots ,t_{e}$ are $\Sigma^{⁎}$-monomials. By Corollary 4.6.(2) we have that $\frac{\sigma (t_{i})}{t_{i}}\in G\langle t_{1}\rangle \ldots {\langle t_{i-1}\rangle }^{⁎}={\left(G^{⁎}\right)}_{G}^{G\langle t_{1}\rangle \ldots \langle t_{i-1}\rangle }$ with 1≤i≤p. Thus the Π-monomials $t_{i}$ are $G^{⁎}$-simple. Moreover, the $\Sigma^{⁎}$-monomials $t_{i}$ on top are all $G^{⁎}$-simple by definition. Summarizing (F,σ)≤(E,σ) is simple.  □

In other words, for a reduced and connected difference ring (A,σ) (e.g., if A is a field) the notions of $\Pi \Sigma^{⁎}$-ring extension and simple $\Pi \Sigma^{⁎}$-ring extension are equivalent. The situation becomes rather different if the ring is, e.g., not connected; see Example 2.20. But, for single-rooted $R\Pi \Sigma^{⁎}$-extensions over a difference field, the situation is again tame.

Corollary 4.15 *A single-rooted* $R\Pi \Sigma^{⁎}$*-extension* (E,σ) *of a field* (G,σ) *is simple.*

Proof By definition the $R\Pi \Sigma^{⁎}$-extension can be reordered to the form as given in Definition 2.21. Since G is a field, $\mathrm{sconst}\;G\setminus \{0\}=G^{⁎}$. By Proposition 4.14 the Π-extension $\left(G\langle t_{1}\rangle \ldots \langle t_{r}\rangle ,\sigma \right)$ of (G,σ) is simple. Since $\frac{\sigma (x_{i})}{x_{i}}\in G^{⁎}$ for 1≤i≤u, the *R*-monomials $x_{i}$ are $G^{⁎}$-simple. Since also the $\Sigma^{⁎}$-monomials $s_{i}$ are $G^{⁎}$-simple, we conclude that (G,σ)≤(E,σ) is simple.  □

## 5 The algorithmic machinery I: order, period, factorial order

An important ingredient for the development of our summation algorithms is the knowledge of the order (see its definition in (10) and the corresponding Problem O), the period and the factorial order. In (A,σ) we define the period of $h\in A^{⁎}$ by

$$\mathrm{per}(h)=\left\{ \begin{array}{cc} 0 & \text{if }∄n>0\text{ s.t. }\sigma^{n}(h)=h\\ \min\{n>0|\;\sigma^{n}(h)=h\} & \text{otherwise}; \end{array}\right.$$

and the factorial order of *h* by

$$\mathrm{ford}(h)=\left\{ \begin{array}{cc} 0 & \text{if }∄n>0\text{ s.t. }h_{\left(n\right)}=1\\ \min\{n>0|\;h_{\left(n\right)}=1\} & \text{otherwise}. \end{array}\right.$$

Using the properties of the automorphism *σ* and Lemma 3.4 it is easy to see that the Z-modules generated by ord(h), per(h) and ford(h) are $\langle \mathrm{ord}(h)\rangle =\mathrm{ord}(h)\;Z=\{k\in Z|\;h^{k}=1\}$, $\langle \mathrm{per}(h)\rangle =\mathrm{per}(h)\;Z=\{k\in Z|\;\sigma^{k}(h)=h\}$, and $\langle \mathrm{ford}(h)\rangle =\mathrm{ford}(h)\;Z=\{k\in Z|\;h_{\left(k\right)}=1\}$, respectively. In addition, the following basic properties hold.

Lemma 5.1 *Let* (A,σ) *be a difference ring with* $\alpha ,h\in A^{⁎}$*. Then the following holds.*

(1) *If* $\alpha \in {\left(\mathrm{const}\;A\right)}^{⁎}$*, then* per(α)=1 *and* ford(α)=ord(α)*.*
(2) *If* σ(h)=αh*, then* per(h)=ford(α)*.*
(3) *If* ord(α)>0 *and* per(α)>0*, then* per(α)|ford(α)|per(α)ord(α) *and*
  (23) $$\mathrm{ford}(\alpha )=\min(i\;\mathrm{per}(\alpha )|\;1\leq i\leq \mathrm{ord}(\alpha )\text{and}\alpha_{\left(i\;\mathrm{per}(\alpha )\right)}=1)>0.$$

Proof (1) Since σ(α)=α, per(α)=1. Since $\alpha_{\left(n\right)}=\alpha^{n}$ for n≥0, ford(α)=ord(α).

(2) By Lemma 3.4.(4) we have that $\sigma^{n}(h)=h$ iff $\alpha_{\left(n\right)}=1$. Hence per(h)=ford(α).

(3) Take p=per(α)>0 and v=ord(α)>0. Then we have that

$$\alpha_{\left(p\;v\right)}=\alpha \;\sigma (\alpha )\ldots \sigma^{p\;v-1}(\alpha )={\left(\alpha \;\sigma (\alpha )\ldots \sigma^{p-1}(\alpha )\right)}^{v}=\alpha^{v}\;\sigma (\alpha^{v})\ldots \sigma^{p-1}(\alpha^{v})=1.$$

Consequently, we can choose n=ord(α)per(α) to obtain $\alpha_{\left(n\right)}=1$. In particular, for any i≥0 with $\alpha_{\left(i\right)}=1$ we have that $1=\frac{\sigma (1)}{1}=\frac{\sigma (\alpha_{\left(i\right)})}{\alpha_{\left(i\right)}}=\frac{\sigma^{i}(\alpha )}{\alpha }$. Hence per(α)|i. Thus the smallest *λ* with $\alpha_{\left(\lambda \right)}=1$ is given by (23). In particular, per(α)|ford(α)|ord(α)per(α).  □

We will present methods to calculate the order, period and factorial order for the elements of ${\left(A^{⁎}\right)}_{A}^{E}$ of a simple *R*-extension (E,σ)≥(G,σ) by recursion. First, we assume that the orders of the *R*-monomials in (E,σ)≥(G,σ) are already computed and show how the orders of the elements of ${\left(A^{⁎}\right)}_{A}^{E}$ can be determined.

Lemma 5.2 *Let* (E,σ) *with* $E=A\langle x_{1}\rangle \ldots \langle x_{e}\rangle$ *be an R-extension of* (A,σ) *and define*

(24) $$\alpha :=u\;x_{1}^{z_{1}}\ldots x_{e}^{z_{e}}\in {\left(A^{⁎}\right)}_{A}^{E}$$

*with* $u\in A^{⁎}$ *and* $z_{i}\in N$*. Then* ord(α)>0 *iff* ord(u)>0*. If* ord(u)>0*, then*

(25) $$\mathrm{ord}(\alpha )=\mathrm{lcm}(\mathrm{ord}(u),\frac{\mathrm{ord}(x_{1})}{\gcd(\mathrm{ord}(x_{1}),z_{1})},\ldots ,\frac{\mathrm{ord}(x_{e})}{\gcd(\mathrm{ord}(x_{e}),z_{e})}).$$

Proof If e=0, the lemma holds. Now let n:=ord(α)>0. Suppose that $1\neq {\left(x_{1}^{z_{1}}\ldots x_{e}^{z_{e}}\right)}^{n}=x_{1}^{n\;z_{1}}\ldots x_{e}^{n\;z_{e}}$. Let *i* be maximal such that $\mathrm{ord}(x_{i})∤z_{i}\;n$. Then there is an *s* with $0<s<\mathrm{ord}(x_{i})$ with $x_{i}^{\mathrm{ord}\;x_{i}-s}=u^{n}\;x_{1}^{z_{1}}\ldots x_{i-1}^{z_{i-1}}\in A\langle x_{1}\rangle \ldots \langle x_{i-1}\rangle$ which contradicts to the construction that $x_{i}^{\mathrm{ord}(x_{i})}=1$ is the defining relation of the *R*-monomial. Thus ${\left(x_{1}^{z_{1}}\ldots x_{e}^{z_{e}}\right)}^{n}=1$ and $u^{n}=1$, i.e., ord(u)>0 and $\mathrm{ord}(x_{1}^{z_{1}}\ldots x_{e}^{z_{e}})>0$. In particular, $\mathrm{ord}(\alpha )=\mathrm{lcm}(\mathrm{ord}(u),\mathrm{ord}(x_{1}^{z_{1}}\ldots x_{e}^{z_{e}}))$. By similar arguments we can show that ${\left(x_{1}^{z_{1}}\right)}^{n}=\cdots ={\left(x_{e}^{z_{e}}\right)}^{n}=1$ and consequently $\mathrm{ord}(x_{1}^{z_{1}}\ldots x_{e}^{z_{e}})=\mathrm{lcm}(\mathrm{ord}(x_{1}^{z_{1}}),\ldots ,\mathrm{ord}(x_{e}^{z_{e}})$. Since also $\mathrm{ord}(x_{i}^{z_{i}})=\frac{\mathrm{ord}(x_{i})}{\gcd(\mathrm{ord}(x_{i}),z_{i})}$ holds, the identity (25) is proven.

Conversely, suppose that ord(u)>0. Then the value of the right hand side of (25) is positive. Denote it by *n*. Then one can check that $\alpha^{n}=1$. Therefore ord(α)>0.  □

In the next lemma we set the stage to calculate the period and factorial order.

Lemma 5.3 *Let* (E,σ) *with* $E=A\langle x_{1}\rangle \ldots \langle x_{e}\rangle$ *be an R-extension of* (A,σ) *where we have* $\mathrm{per}(x_{i})>0$ *for* 1≤i≤e*. Let* $\alpha \in {\left(A^{⁎}\right)}_{A}^{E}$ *as in* (24) *with* $z_{1},\ldots ,z_{e}\in N$ *and* $u\in A^{⁎}$*.*

(1) *Then* per(α)>0 *iff* per(u)>0*. If* per(u)>0*, then*
  (26) $$\mathrm{per}(\alpha )=\min(1\leq j\leq \mu |\;\sigma^{j}(\alpha )=\alpha \text{and}j|\mu )$$
  *with* $\mu =\mathrm{lcm}(\mathrm{per}(u),\mathrm{per}(x_{i_{1}}),\ldots ,\mathrm{per}(x_{i_{k}}))$ *where* $\left\{i_{1},\ldots ,i_{k}\}=\{i:\;\mathrm{ord}(x_{i})∤z_{i}\right\}$*.*
(2) *We have that* ford(α)>0 *iff* ford(u)>0*.*
(3) *If* per(u),ord(u)>0*, then* ford(α)>0 *and* 0<per(α)|ford(α)|per(α)ord(α)*.*
(4) *If the values* $\mathrm{ord}(x_{i})$ *and* $\mathrm{per}(x_{i})$ *for* 1≤i≤e *and the values* per(u)>0 *and* ord(u)>0 *are given explicitly, then* per(α) *and* ford(α) *can be calculated.*

Proof (1) Suppose that per(u)>0. Then μ>0. In particular, it follows that $\sigma^{\mu }(\alpha )=\alpha$. Consequently, per(α)>0 with per(α)|μ. Hence we have (26). Conversely, suppose that per(α)>0. Then with $\nu :=\mathrm{lcm}(\mathrm{per}(\alpha ),\mathrm{per}(x_{1}),\ldots ,\mathrm{per}(x_{e}))>0$ we get $u\;x_{1}^{z_{1}}\ldots x_{e}^{z_{e}}=\alpha =\sigma^{\nu }(\alpha )=\sigma^{\nu }(u)\;x_{1}^{z_{1}}\ldots x_{e}^{z_{e}}$. Thus $\sigma^{\nu }(u)=u$, and consequently ord(u)>0.

(2) Since $\mathrm{ord}(x_{i})$ and $\mathrm{per}(x_{i})>0$, it follows that $\mathrm{ford}(x_{i})>0$ by Lemma 5.1.(3) for all 1≤i≤e. If ford(u)>0, take $\nu =\mathrm{lcm}(\mathrm{ford}(u),\mathrm{ford}(x_{1}),\ldots ,\mathrm{ford}(x_{e}))>0$. By Lemma 3.4.(1), $\alpha_{\left(\nu \right)}=1$ and hence ford(α)>0. Conversely, if ford(α)>0, take $\nu^{\prime }=\mathrm{lcm}(\mathrm{ford}(\alpha ),\mathrm{ford}(x_{1}),\ldots ,\mathrm{ford}(x_{e}))>0$. Then again by Lemma 3.4.(1): $1=\alpha_{\left(\nu^{\prime }\right)}={\left(u\;x_{1}^{z_{1}}\ldots x_{e}^{z_{e}}\right)}_{\left(\nu^{\prime }\right)}=u_{\left(\nu^{\prime }\right)}$. Thus ford(u)>0.

(3) By part 1, per(α)>0. And with ord(u)>0 and Lemma 5.2 it follows that ord(α)>0. By Lemma 5.1.(3), per(α)|ford(α)|ord(α)per(α). In particular, ford(α)>0.

(4) If per(u) and the values $\mathrm{per}(x_{i})$ are given, *μ* from part 1 can be computed. In particular, if ord(u) and $\mathrm{ord}(x_{i})$ are given explicitly, ord(α) can be calculated by Lemma 5.2. Thus per(α) can be determined by (26) and then ford(α) can be computed by (23).  □

Example 5.4

(1) Take α=u=−1∈Q. We get ord(α)=2. In addition, per(−1)=1. Moreover, 1=per(−1)|ford(−1)|per(−1)ord(−1)=2. Hence (26) yields ford(−1)=2.
(2) Consider the *R*-extension (Q[x],σ) of (Q,σ) with σ(x)=−x and ord(x)=2, and take α=−x. We get ord(α)=lcm(ord(−1),ord(x))=2 by (25). With μ=lcm(per(−1),per(x))=2 we get per(α)=2 by using (26). Furthermore, we get 2=per(α)|ford(α)|per(α)ord(α)=4. Hence with (26) we get ford(α)=4.
(3) Consider the *R*-extension (K(k)[x],σ) of (K(k),σ) with σ(x)=ιx and ord(x)=4 from Example 2.14. We have per(x)=4. Take α=x. We obtain the following bounds 4=per(α)|ford(α)|per(α)ord(α)=16. Thus with (26) we determine ford(α)=8.

Combining the two lemmas from above we arrive at the following result.

Proposition 5.5 *Let* $\left(A\langle x_{1}\rangle \ldots \langle x_{e}\rangle ,\sigma \right)$ *be a simple R-extension of* (A,σ) *such that for* 1≤i≤e *we have that* $\sigma (x_{i})/x_{i}=u_{i}\;x_{1}^{m_{i,1}}\ldots x_{i-1}^{m_{i,i-1}}$ *with* $u_{i}\in A^{⁎}$ *and* $m_{i,j}\in N$*. Then the following holds.*

(1) $\mathrm{ord}(u_{i})>0$ *for* 1≤i≤e*. In particular, if the values* $\mathrm{ord}(u_{i})$ *are given explicitly (are computable), then the values* $\mathrm{ord}(x_{i})$ *are computable.*
(2) *If* $\mathrm{per}(u_{i})>0$ *for* 1≤i≤e*, then* $\mathrm{per}(x_{i})>0$ *for* 1≤i≤e*. In particular, if the values of* $\mathrm{ord}(u_{i})$ *and* $\mathrm{per}(u_{i})$ *for* 1≤i≤e *are given explicitly (are computed), the values* $\mathrm{per}(x_{i})$ *for all* 1≤i≤e *are computable.*

Proof (1) By iterative application of Lemma 5.3 it follows that $\mathrm{ord}(u_{i})>0$ for all 1≤i≤e. Moreover, suppose that $\mathrm{ord}(u_{i})$ is given for 1≤i≤e. Furthermore, assume that the values $\mathrm{ord}(x_{i})$ for 1≤i≤s with s<e are already determined. Then define $\alpha =\sigma (x_{s})/x_{s}$. By (25) we obtain ord(α) and thus $\mathrm{ord}(x_{s})=\mathrm{ord}(\alpha )$ by Theorem 2.12.(3). This completes the induction step.

(2) Suppose that $\mathrm{per}(u_{i})>0$ for 1≤i≤e. In addition, suppose that we have shown already that $d_{i}=\mathrm{per}(x_{i})>0$ for 1≤i<s with s≤e. Define $\alpha =\sigma (x_{s})/x_{s}$. By Lemma 5.3 we have per(α)>0 and ford(α)>0. By Lemma 5.1.(2) it follows that $\mathrm{per}(x_{s})=\mathrm{ford}(\alpha )>0$. If the values $\mathrm{ord}(u_{i})$ are given explicitly, we can compute ord(α) by part 1. If $\mathrm{per}(u_{s})$ is given explicitly and $d_{1},\ldots ,d_{s-1}$ are given (are already computed), per(α) can be computed with Lemma 5.1.(3). Hence ford(α) can be calculated with (23). Thus we get $\mathrm{ord}(x_{s})=\mathrm{ford}(\alpha )$ by Lemma 5.1.(2) which completes the induction step.  □

If we restrict to the case that the ground domain is a field F and all roots of unity of F are constants, we end up at the following properties of *R*-extensions.

Corollary 5.6 *Let* (E,σ) *with* $E=F\langle x_{1}\rangle \ldots \langle x_{e}\rangle$ *be a simple R-extension of a difference field* (F,σ) *with constant field* K *such that all roots of unity in* F *are constants (e.g., if* (F,σ) *is strong constant-stable). Then the following holds.*

(1) *For* 1≤i≤e *we have that*
  (27) $$\sigma (x_{i})/x_{i}=u_{i}\;x_{1}^{m_{i,1}}\ldots x_{i-1}^{m_{i,i-1}}$$
  *for some root of unity* $u_{i}\in K^{⁎}$ *with* $\mathrm{ord}(u_{i})|\mathrm{ord}(x_{i})$ *and* $m_{i,j}\in N$*.*
(2) $\left(K\langle x_{1}\rangle \ldots \langle x_{e}\rangle ,\sigma \right)$ *is a simple R-extension of* (K,σ)*.*
(3) *Let* $\alpha =u\;x_{1}^{z_{1}}\ldots x_{e}^{z_{e}}\in {\left(K^{⁎}\right)}_{K}^{K\langle x_{1}\rangle \ldots \langle x_{e}\rangle }$ *with* $z_{1},\ldots ,z_{e}\in N$ *and* $u\in K^{⁎}$*. Then*
  ord(u)>0⇔ord(α)>0⇔per(α)>0⇔ford(α)>0.
(4) *If* (K,σ) *is computable and Problem O is solvable in* $K^{⁎}$ *then the values of* ord(α)*,* per(α) *and* ford(α) *are computable for all* $\alpha \in {\left(K^{⁎}\right)}_{K}^{K\langle x_{1}\rangle \ldots \langle x_{e}\rangle }$*.*
(5) *Problem O is solvable in* ${\left(F^{⁎}\right)}_{F}^{E}$ *if it is solvable in* $K^{⁎}$ *and* (F,σ) *is computable.*

Proof (1) By definition we have that (27) with $m_{i}\in N$ and $u_{i}\in F^{⁎}$. By Lemma 5.2 it follows that $\mathrm{ord}(u_{i})>0$ and $\mathrm{ord}(u_{i})|\mathrm{ord}(x_{i})$. In particular, $u_{i}\in K^{⁎}$ since all roots of unity from F are constants by assumption.

(2) It is immediate that (H,σ) with $H=K\langle x_{1}\rangle \ldots \langle x_{e}\rangle$ forms a difference ring. Since constE=constF=K, (H,σ) is a simple *R*-extension of (K,σ).

(3) By part 1 we get $u_{i}\in K^{⁎}$ and $\mathrm{ord}(u_{i})>0$ for 1≤i≤e. In particular, $\mathrm{per}(u_{i})=1$. With Proposition 5.5 we get $\mathrm{per}(x_{i})>0$, and by Lemma 5.1.(1) we obtain per(u)=1 and ford(u)=ord(u). Thus the equivalences follow by Lemmas 5.2 and 5.3 (parts 1, 2).

(4) Since $u_{i}\in K^{⁎}$, the values of $\mathrm{ord}(u_{i})>0$ can be determined by solving Problem O in $K^{⁎}$. Thus by Proposition 5.5 the orders and periods of the $x_{i}$ can be computed. Let $\alpha :=u\;x_{1}^{z_{1}}\ldots x_{e}^{z_{e}}$ with $u\in K^{⁎}$ and $z_{i}\in N$. Then by Lemma 5.2 and the computation of ord(u) the order of *α* can be computed. Moreover, since per(u)=1 and ord(u)=ford(u) are given, we can invoke Lemma 5.3 to calculate the period and factorial order of *α*.

(5) Let *α* be given as in (24) with $u\in F^{⁎}$ and $m_{i}\in N$. By Lemma 5.2 ord(α)>0 iff ord(u)>0. By assumption, ord(u)>0 implies $u\in K^{⁎}$. Thus, if u∉K, ord(α)=0. Otherwise, if $u\in K^{⁎}$, we can apply part 4.  □

Finally, we are in the position to prove Theorem 2.26.(1).

Proof 5.7 **Theorem 2.26.(1)**

Let (E,σ) be a simple $R\Pi \Sigma^{⁎}$-extension of (F,σ) where (F,σ) is computable and where any root of unity of F is from K=constF. Reorder it to the shape as given in Lemma 4.10. In particular, the *R*-extension $\left(F\langle t_{1}\rangle \ldots \langle t_{r}\rangle ,\sigma \right)$ of (F,σ) has the shape as given in Corollary 5.6.(1). Let $f\in {\left(F^{⁎}\right)}_{F}^{E}$. Suppose first that *f* depends on a Π-monomial $t_{i}$. Now assume that ord(f)=n>0, and let *i* be maximal such that a Π-monomial depends on *f*. Then $f=v\;t_{i}^{m}$ with $v\in F\langle t_{1}\rangle \ldots {\langle t_{i-1}\rangle }^{⁎}$ and m∈Z∖{0}. Hence $1=f^{n}=v^{n}\;t_{i}^{m\;n}$ and thus $t_{i}$ is not algebraically independent over $F\langle t_{1}\rangle \ldots \langle t_{i-1}\rangle$; a contradiction. Consequently, if *f* depends on Π-monomials, ord(f)=0. Otherwise, $f=u\;t_{1}^{m_{1}}\ldots t_{r}^{m_{r}}$ with $u\in F^{⁎}$ and $m_{i}\in N$ where the $t_{i}$ are all *R*-monomials. Therefore the value ord(f) can be computed by Corollary 5.6.(5).  □

## 6 The algorithmic machinery II: Problem PMT

We aim at proving Theorems 2.23.(1) and 2.26.(2), i.e., providing recursive algorithms that reduce Problem PMT from a given $R\Pi \Sigma^{⁎}$-extension to its ground ring (resp. field). For this reduction we assume that for the given ground ring (G,σ) and given group $G\leq G^{⁎}$ we have that ${\mathrm{sconst}}_{G}\;G\setminus \{0\}\leq G^{⁎}$. This property guarantees that for any $f\in G^{n}$ a Z-basis of M(f,G) with rank≤n exists; see Lemma 2.16. In particular, we rely on the fact that there are algorithms available that solve Problem PMT in (G,σ) for *G*. For concrete classes of difference fields (G,σ) with these algorithmic properties we refer to Subsection 2.3.3.

### 6.1 A reduction strategy for $\Pi \Sigma^{⁎}$-extensions

First, we treat the reduction for $\Pi \Sigma^{⁎}$-extensions. More precisely, we will obtain

Theorem 6.1 *Let* (G,σ) *be a computable difference ring with* $G\leq G^{⁎}$ *where* ${\mathrm{sconst}}_{G}\;G\setminus \{0\}\leq G^{⁎}$*. Let* (E,σ) *be a G-simple* $\Pi \Sigma^{⁎}$*-extension of* (G,σ)*. Then* ${\mathrm{sconst}}_{G_{G}^{E}}\;E\setminus \{0\}\leq E^{⁎}$ *and Problem PMT is solvable in* (E,σ) *for* $G_{G}^{E}$ *if it is solvable in* (G,σ) *for G.*

For the underlying reduction method we use the following two lemmas.

Lemma 6.2 *Let* (A[t],σ) *be a* $\Sigma^{⁎}$*-extension of* (A,σ) *and let* $H\leq A^{⁎}$ *be a group with* ${\mathrm{sconst}}_{H}\;A\setminus \{0\}\leq A^{⁎}$*. Then for* $f\in H^{n}$ *we have that* M(f,A[t])=M(f,A)*.*

Proof “⊆”: Let $m=(m_{1},\ldots ,m_{n})\in M(f,A[t])$ with $f=(f_{1},\ldots ,f_{n})\in H^{n}$. Thus take g∈A[t]∖{0} with $\sigma (g)=f_{1}^{m_{1}}\ldots f_{n}^{m_{n}}\;g$. Since $g\in {\mathrm{sconst}}_{H}\;A[t]\setminus \{0\}$, we have $g\in {\mathrm{sconst}}_{H}\;A$ by Theorem 3.12. Hence m∈M(f,A). The inclusion ⊇ is obvious.  □

Lemma 6.3 *Let* (A〈t〉,σ) *be a* Π*-extension of* (A,σ) *and let* $H\leq A^{⁎}$ *with* ${\mathrm{sconst}}_{H}\;A\setminus \{0\}\leq A^{⁎}$ *and* α:=σ(t)/t∈H*. Let* $f=(f_{1},\ldots ,f_{n})\in {\left(H_{A}^{A\langle t\rangle }\right)}^{n}$ *with*

$$f_{i}=h_{i}\;t^{e_{i}},\;h_{i}\in H,\;e_{i}\in Z.$$

*Then* $M(f,A\langle t\rangle )=M_{1}\cap M_{2}$ *where*

$$M_{1}=\{(m_{1},\ldots ,m_{n})|\;(m_{1},\ldots ,m_{n},m_{n+1})\in M((h_{1},\ldots ,h_{n},\frac{1}{\alpha }),A)\},$$

$$M_{2}={\mathrm{Ann}}_{Z}((e_{1},\ldots ,e_{n}))=\{(m_{1}\ldots ,m_{n})\in Z^{n}|\;m_{1}\;e_{1}+\cdots +m_{n}\;e_{n}=0\}.$$

Proof “⊆”: Let $\left(m_{1},\ldots ,m_{n})\in M(f,A\langle t\rangle \right)$. Hence we can take g∈A〈t〉∖{0} with $\sigma (g)=f_{1}^{m_{1}}\ldots f_{n}^{m_{n}}\;g$, i.e., $g\in {\mathrm{sconst}}_{\overset{˜}{H}}\;A\langle t\rangle \setminus \{0\}$ with $\overset{˜}{H}=H_{A}^{A\langle t\rangle }$. Thus by Theorem 3.20 it follows that $g=\overset{˜}{g}\;t^{m}$ with m∈Z and $\overset{˜}{g}\in {\mathrm{sconst}}_{\overset{˜}{H}}\;A\setminus \{0\}\leq A^{⁎}$. Hence

$$\sigma (\overset{˜}{g})=f_{1}^{m_{1}}\ldots f_{n}^{m_{n}}\alpha^{-m}\overset{˜}{g}=h_{1}^{m_{1}}\ldots h_{n}^{m_{n}}\;\alpha^{-m}\;\overset{˜}{g}\;t^{m_{1}\;e_{1}+\cdots +m_{n}\;e_{n}}.$$

Since $\overset{˜}{g}\neq 0$, we conclude that $\sigma (\overset{˜}{g})\neq 0$. By coefficient comparison it follows then that $m_{1}\;e_{1}+\cdots +m_{n}\;e_{n}=0$, i.e., $(m_{1},\ldots ,m_{n})\in M_{2}$. Thus $\sigma (\overset{˜}{g})=h_{1}^{m_{1}}\ldots h_{n}^{m_{n}}\alpha^{-m}\;\overset{˜}{g}$ and consequently $\left(m_{1},\ldots ,m_{n},m)\in M((h_{1},\ldots ,h_{n},\frac{1}{\alpha }),A\right)$, i.e., $(m_{1},\ldots ,m_{n})\in M_{1}$.

“⊇”: Let $(m_{1},\ldots ,m_{n})\in M_{1}\cap M_{2}$. Thus we can take $\overset{˜}{g}\in A\setminus \{0\}$ and m∈Z with $\sigma (\overset{˜}{g})=h_{1}^{m_{1}}\ldots h_{n}^{m_{n}}\alpha^{-m}\overset{˜}{g}$. Moreover, we have that $e_{1}\;m_{1}+\cdots +e_{n}\;m_{n}=0$. Thus $\sigma (\overset{˜}{g}\;t^{m})={\left(h_{1}\;t^{e_{1}}\right)}^{m_{1}}\ldots {\left(h_{n}\;t^{e_{n}}\right)}^{m_{n}}\overset{˜}{g}$ and therefore $\left(m_{1},\ldots ,m_{n})\in M(f,A\langle t\rangle \right)$.  □

Now we can deal with the underlying algorithm resp. proof of Theorem 6.1.

Proof 6.4 **Theorem 6.1**

Let (G,σ) be a difference ring and let $G\leq G^{⁎}$ such that ${\mathrm{sconst}}_{G}\;G\setminus \{0\}\leq G^{⁎}$ holds. Suppose that Problem PMT is solvable in (G,σ) for *G*. Now let (E,σ) be a *G*-simple $\Pi \Sigma^{⁎}$-extension of (G,σ) as in the theorem with $\overset{˜}{G}=G_{G}^{E}$ and let $f\in {\overset{˜}{G}}^{n}$. By Corollary 4.6.(1) it follows that ${\mathrm{sconst}}_{\overset{˜}{G}}\;E\setminus \{0\}\leq E^{⁎}$ and together with Lemma 2.16 it follows that $M(f,E)=M(f,{\mathrm{sconst}}_{\overset{˜}{G}}\;E)$ is a Z-module. The calculation of a basis of M(f,E) will be accomplished by recursion/induction. If E=A, nothing has to be shown. Otherwise, let (A,σ) be a *G*-simple $\Pi \Sigma^{⁎}$-extension of (G,σ) in which we know how one can solve Problem PMT for $H=G_{G}^{A}$, and let E=A〈t〉 where *t* is an *H*-simple $\Pi \Sigma^{⁎}$-monomial. We have to treat two cases. First, suppose that *t* is a $\Sigma^{⁎}$-monomial. Then it follows that $\overset{˜}{G}=G_{G}^{E}=G_{G}^{A}=H\leq A^{⁎}$ and thus $f\in H^{n}$. Hence we can activate Lemma 6.2 and it follows that M(f,E)=M(f,A). Thus by assumption we can compute a basis. Second, suppose that *t* is an *H*-simple Π-monomial. Then we can utilize Lemma 6.3: We calculate a basis of $M_{2}$ by linear algebra. Furthermore, we compute a basis of $M((h_{1},\ldots ,h_{n},\frac{1}{\alpha }),A)$ by the induction assumption (by recursion). Hence we can derive a basis of $M_{2}$ and thus of $M_{1}\cap M_{2}=M(f,A\langle t\rangle )$. This completes the proof.  □

Note that the reduction presented in Lemma 6.3 is accomplished by increasing the rank of $M_{1}$ by one. In general, the more Π-monomials are involved, the higher the rank will be in the arising Problems PMT of the recursions.

Looking closer at the reduction algorithm, we can extract the following shortcut, resp. a refined version of Theorem 2.12.(2).

Corollary 6.5 *Let* (A,σ) *be a difference ring and let* $G\leq A^{⁎}$ *with* ${\mathrm{sconst}}_{G}\;A\setminus \{0\}\leq A^{⁎}$*. Let* (H,σ) *be a G-simple* Π*-extension of* (A,σ) *and let* (E,σ) *be a* $\Sigma^{⁎}$*-extension of* (H,σ)*. Then* $G_{A}^{E}=G_{A}^{H}$ *and the following holds.*

(1) M(f,E)=M(f,H) *for any* $f\in {\left(G_{A}^{E}\right)}^{n}$*.*
(2) *Let* $\alpha \in G_{A}^{E}$*. Then there is a* Π*-extension* (E〈t〉,σ) *of* (E,σ) *with* σ(t)=αt *iff there is a* Π*-extension* (H〈t〉,σ) *of* (H,σ) *with* σ(t)=αt*.*

Proof Note that $G_{A}^{E}\leq H^{⁎}$. Hence by iterative application of Lemma 6.2 part 1 is proven. Part 2 follows by part 1 and Theorem 2.12.(2).  □

If one restricts to the special case that (G,σ) is a $\Pi \Sigma^{⁎}$-field with $G=G^{⁎}$, the presented reduction techniques boil down to the reduction presented in Karr (1981, Theorem 8). The major contribution here is that Theorem 6.1 can be applied for any computable difference ring (G,σ) with the properties given in Theorem 6.1. Subsequently, we utilize this additional flexibility to tackle (nested) *R*-extensions.

### 6.2 A reduction strategy for *R*-extensions and thus for $R\Pi \Sigma^{⁎}$-extensions

First, we treat the special case of single-rooted and simple *R*-extensions.

Lemma 6.6 *Let* (A,σ) *be a difference ring and let* $G\leq A^{⁎}$ *with* ${\mathrm{sconst}}_{G}\;A\setminus \{0\}\leq A^{⁎}$*. Let* (A[x],σ) *be an R-extension of* (A,σ) *with* σ(x)=αx *where* α∈G*; let* $f=(f_{1},\ldots ,f_{n})\in G^{n}$*. Then* $M(f,A[x])=\{(m_{1},\ldots ,m_{n})|\;(m_{1},\ldots ,m_{n+1})\in M((f_{1},\ldots ,f_{n},\frac{1}{\alpha }),A)$*.*

Proof Let $\left(m_{1},\ldots ,m_{n})\in M(f,A[x]\right)$. Hence there is a $g\in {\mathrm{sconst}}_{G}\;A[x]\setminus \{0\}$ with $\sigma (g)=f_{1}^{m_{1}}\ldots f_{n}^{m_{n}}\;g$. By Proposition 3.23 it follows that $g=\overset{˜}{g}\;x^{m}$ with $\overset{˜}{g}\in A\setminus \{0\}$ and m∈N. Thus

(28) $$\sigma (\overset{˜}{g})=f_{1}^{m_{1}}\ldots f_{n}^{m_{n}}\;\alpha^{-m}\;\overset{˜}{g}$$

and hence $\left(m_{1},\ldots ,m_{n},m)\in M((f_{1},\ldots ,f_{n},\frac{1}{\alpha }),A\right)$. Conversely, if $\left(m_{1},\ldots ,m_{n},m)\in M((f_{1},\ldots ,f_{n},1/\alpha ,A\right)$, there is a $\overset{˜}{g}\in A\setminus \{0\}$ with (28). Therefore we conclude that $\sigma (\overset{˜}{g}\;t^{m})=f_{1}^{m_{1}}\ldots f_{n}^{m_{n}}\;\overset{˜}{g}\;t^{m}$ which implies that $\left(m_{1},\ldots ,m_{n})\in M(f,A[x]\right)$.  □

As a consequence we obtain the proof of our Theorem 2.23.(1).

Proof 6.7 **Theorem 2.23.(1)**

Since Problem PMT is solvable in (G,σ) for *G*, it follows by Theorem 6.1 that Problem PMT is solvable in (H,σ) for $\overset{˜}{G}$ with $H=G\langle t_{1}\rangle \ldots \langle t_{r}\rangle$ and that ${\mathrm{sconst}}_{\overset{˜}{G}}\;H\setminus \{0\}\leq H^{⁎}$. Thus by iterative applications of Lemma 6.6 and Proposition 3.23 we conclude that Problem PMT is solvable in $\left(\overset{¯}{H},\sigma \right)$ for $\overset{˜}{G}$ with $\overset{¯}{H}=H\langle x_{1}\rangle \ldots \langle x_{u}\rangle$ and that ${\mathrm{sconst}}_{\overset{˜}{G}}\;\overset{¯}{H}\setminus \{0\}\leq {\overset{¯}{H}}^{⁎}$. Finally, by applying again Theorem 6.1 it follows that Problem PMT is solvable in (E,σ) for $\overset{˜}{G}$.  □

In order to tackle the more general case that the *R*-extensions are nested and that they might occur also in Π-extensions (see the underlying algorithms for Theorem 2.26.(2) in Proof 6.15 below), we require additional properties on the difference rings: they must be strong constant-stable; see Definition 2.25. With this extra condition the following structural property of the semi-constants holds. They factor into two parts: a factor which depends only on the *R*-monomials with constant coefficients and a factor which is free of the *R*-monomials.

Lemma 6.8 *Let* (A,σ) *be a difference ring which is constant-stable and let* $G\leq A^{⁎}$ *be closed under σ where* ${\mathrm{sconst}}_{G}(A,\sigma^{k})\setminus \{0\}\leq A^{⁎}$ *for any* k>0*. Let* (E,σ) *be a simple R-extension of* (A,σ) *with* $E=A[x_{1}]\ldots [x_{e}]$ *where we have* (27) *with* $m_{i,j}\in N$ *and* $u_{i}\in G$ *with* $\mathrm{per}(u_{i})>0$*. Define*

(29) $$r:=\left\{ \begin{array}{cc} \mathrm{lcm}(\mathrm{ford}(u_{1}),\ldots ,\mathrm{ford}(u_{e}),\mathrm{ford}(x_{1}),\ldots ,\mathrm{ford}(x_{e})) & \text{if}e>0\\ 1 & \text{if}e=0. \end{array}\right.$$

*Let* $\overset{˜}{G}=G_{A}^{E}$ *with* ${\mathrm{sconst}}_{\overset{˜}{G}}\;E\setminus \{0\}\leq E^{⁎}$*. Then the following holds.*

(1) r>0*.*
(2) *For any* $g\in {\mathrm{sconst}}_{\overset{˜}{G}}\;E\setminus \{0\}$ *we have that*
  (30) $$g=\overset{˜}{g}\;h$$
  *with* $\overset{˜}{g}\in {\mathrm{sconst}}_{G}(A,\sigma^{r})\setminus \{0\}\leq A^{⁎}$ *and* $h\in \mathrm{const}{\left(K[x_{1},\ldots ,x_{e}],\sigma^{r}\right)}^{⁎}$*.*
(3) *If* $\sigma (g)=v\;x_{1}^{m_{1}}\ldots x_{e}^{m_{e}}\;g$ *with* v∈G*,* $m_{i}\in N$*, then* $\sigma (\overset{˜}{g})=\lambda \;v\;\overset{˜}{g}$ *with* $\lambda \in A^{⁎}$*,* $\lambda^{r}=1$*.*

Proof Let $g\in {\mathrm{sconst}}_{\overset{˜}{G}}\;E\setminus \{0\}$, i.e., $\sigma (g)=v\;x_{1}^{m_{1}}\ldots x_{e}^{m_{e}}\;g$ with v∈G and $m_{i}\in Z$. Let *r* be given as in (29). If e=0, i.e., r=1, the lemma holds by taking $\overset{˜}{g}:=g\in A^{⁎}$ and h=1. Otherwise, we may suppose that e>0.

(1) Let 1≤i≤e. By Proposition 5.5.(1) it follows that $\mathrm{ord}(u_{i})>0$. Together with the assumption that $\mathrm{per}(u_{i})>0$ we have that $\mathrm{ford}(u_{i})>0$ by Lemma 5.1.(3). Moreover, by Proposition 5.5.(2) it follows that $\mathrm{per}(x_{i})>0$. Again with $\mathrm{ord}(x_{i})>0$ and $\mathrm{per}(x_{i})>0$ it follows that $\mathrm{ford}(x_{i})>0$ by Lemma 5.1.(3). Therefore r>0.

(2) By the choice of *r* it follows that for all 1≤i≤e we have

(31) $${\left(u_{i}\right)}_{\left(r\right)}=1,\;{\left(x_{i}\right)}_{\left(r\right)}=1\text{ and }\sigma^{r}(x_{i})=x_{i};$$

the last equality follows by Lemma 5.3.(3). Moreover, by Lemma 3.4 we conclude that

$$\sigma^{r}(g)={\left(v\;x_{1}^{m_{1}}\ldots x_{e}^{m_{e}}\right)}_{\left(r\right)}\;g=v_{\left(r\right)}\;{\left(x_{1}\right)}_{\left(r\right)}^{m_{1}}\ldots {\left(x_{e}\right)}_{\left(r\right)}^{m_{e}}\;g=\overset{˜}{u}\;g$$

with $\overset{˜}{u}:=v_{\left(r\right)}$. Since *G* is closed under *σ*, we have that $\overset{˜}{u}\in G$. Write $g=\sum_{s\in S}g_{s}x^{s}$ where $S\subseteq N^{e}$ is finite, $g_{s}\in F^{⁎}$ and for $(s_{1},\ldots ,s_{e})\in S$ and $x=(x_{1},\ldots ,x_{e})$ we use the multi-index notation $x^{s}=x_{1}^{s_{1}}\ldots x_{e}^{s_{e}}$. In particular, we suppose that if $s,s^{\prime }\in S$ with $x^{s}=x^{s^{\prime }}$ then $s=s^{\prime }$. Then by coefficient comparison w.r.t. $x^{i}$ and using (31) we obtain $\sigma^{r}(g_{i})=\overset{˜}{u}\;g_{i}$ for any i∈S. Note that $g_{i}\in {\mathrm{sconst}}_{G}(A,\sigma^{r})\setminus \{0\}\leq A^{⁎}$. Hence for any s,r∈S we have that $\sigma^{r}(g_{s}/g_{r})=g_{s}/g_{r}$. Thus it follows that $g_{s}/g_{r}\in {\left(\mathrm{const}(A,\sigma^{r})\right)}^{⁎}=K^{⁎}$, i.e., for all s∈S we have that $g_{s}=c_{s}\;\overset{˜}{g}$ for some $c_{s}\in K^{⁎}$ and $\overset{˜}{g}\in {\mathrm{sconst}}_{G}(A,\sigma^{r})\setminus \{0\}\leq A^{⁎}$ with

(32) $$\sigma^{r}(\overset{˜}{g})=\overset{˜}{u}\;\overset{˜}{g}.$$

Consequently, $g=\overset{˜}{g}\;h$ with $h=\sum_{s\in S}c_{s}x^{s}$. Since $g\in A^{⁎}$, $h\in K{\left[x_{1},\ldots ,x_{e}\right]}^{⁎}$. Finally, with (31) we conclude that $h\in \mathrm{const}{\left(K[x_{1},\ldots ,x_{e}],\sigma^{r}\right)}^{⁎}$.

(3) Taking $s=(s_{1},\ldots ,s_{e})\in S$, it is easy to see that there is exactly one $s^{\prime }\in S$ with

$$\sigma (c_{s}x^{s}\overset{˜}{g})=v\;x_{1}^{m_{1}}\ldots x_{e}^{m_{e}}c_{s^{\prime }}x^{s^{\prime }}.$$

This means that on both sides the same monomial $x^{s^{\prime }+(m_{1},\ldots ,m_{e})}$ in reduced form occurs. By coefficient comparison this gives $\sigma (\overset{˜}{g})=v\;u_{1}^{-s_{1}}\ldots u_{e}^{-s_{e}}\;\frac{c_{s^{\prime }}}{c_{s}}\overset{˜}{g}$. Thus with (31) and Lemma 3.4 we get $\sigma^{r}(\overset{˜}{g})=v_{\left(r\right)}{\left(\frac{c_{s^{\prime }}}{c_{s}}\right)}^{r}\overset{˜}{g}=\overset{˜}{u}\;{\left(\frac{c_{s^{\prime }}}{c_{s}}\right)}^{r}\;\overset{˜}{g}$. Hence with (32) we obtain ${\left(\frac{c_{s^{\prime }}}{c_{s}}\right)}^{r}=1$. Finally, with $\lambda :=u_{1}^{-s_{1}}\ldots u_{e}^{-s_{e}}\;\frac{c_{s^{\prime }}}{c_{s}}$ we have that $\sigma (\overset{˜}{g})=\lambda \;v\;\overset{˜}{g}$ with $\lambda^{r}=1$ and $\lambda \in A^{⁎}$.  □

Specializing A to a strong constant-stable difference field, the lemma reads as follows.

Corollary 6.9 *Let* (F,σ) *be a difference field with* K=constF *which is strong constant-stable. Let* (E,σ) *be a simple R-extension of* (F,σ) *with* $E=F[x_{1}]\ldots [x_{e}]$ *such that* (27) *holds with* $m_{i,j}\in N$*,* $u_{i}\in K^{⁎}$*, and define* (29)*. Let* $\overset{˜}{G}={\left(F^{⁎}\right)}_{F}^{E}$*. Then:*

(1) r>0*.*
(2) *For any* $g\in {\mathrm{sconst}}_{\overset{˜}{G}}\;E\setminus \{0\}$ *we have* (30) *with* $\overset{˜}{g}\in F^{⁎}$ *and* $h\in \mathrm{const}{\left(K[x_{1},\ldots ,x_{e}],\sigma^{r}\right)}^{⁎}$*.*
(3) *If* $\sigma (g)=v\;x_{1}^{m_{1}}\ldots x_{e}^{m_{e}}\;g$ *with* $v\in F^{⁎}$*,* $m_{i}\in Z$*, then* $\sigma (\overset{˜}{g})=\lambda \;v\;\overset{˜}{g}$ *with* $\lambda \in K^{⁎}$*,* $\lambda^{r}=1$*.*

Proof Since $u_{i}\in K^{⁎}$ by Corollary 5.6.(1), $\mathrm{per}(u_{i})=1$. Define $G=F^{⁎}$ which is closed under *σ*. In particular, ${\mathrm{sconst}}_{G}(F,\sigma^{k})\setminus \{0\}=F^{⁎}$ for any k>0. In addition, ${\mathrm{sconst}}_{\overset{˜}{G}}\;E\setminus \{0\}\leq E^{⁎}$ by Corollary 4.3. Thus we can apply Lemma 6.8. The corollary follows by observing that $\lambda \in F^{⁎}$ with $\lambda^{r}=1$. Then by our assumption it follows that $\lambda \in K^{⁎}$.  □

With this result we get the following reduction tactic for simple *R*-extensions.

Lemma 6.10 *Let* (F,σ) *be a difference field with* K=constF *which is strong constant-stable. Let* (E,σ) *be a simple R-extension of* (F,σ) *with* $E=F[x_{1}]\ldots [x_{e}]$ *where we have* (27) *with* $m_{i,j}\in N$ *and* $u_{i}\in K^{⁎}$*. Define* r>0 *as given in* (29) *and choose*^8^ *a set* $\{\alpha_{1},\ldots ,\alpha_{s}\}\subseteq K^{⁎}$ *of* rth *roots of unity which generate multiplicatively all* rth *roots of unity of* K*. Let* $G={\left(F^{⁎}\right)}_{F}^{E}$ *and let* $f=(f_{1},\ldots ,f_{n})\in G^{n}$ *with* $f_{i}={\overset{˜}{f}}_{i}\;h_{i}$ *where* ${\overset{˜}{f}}_{i}\in F^{⁎}$ *and* $h_{i}=x_{1}^{z_{i,1}}\ldots x_{e}^{z_{i,e}}$ *with* $z_{i,j}\in N$*. Then*

(33) $$M(f,E)=\{(m_{1},\ldots ,m_{n})|\;(m_{1},\ldots ,m_{n+s})\in M_{1}\cap M_{2}\}$$

*where*

$$M_{1}=M(({\overset{˜}{f}}_{1},\ldots ,{\overset{˜}{f}}_{n},\alpha_{1},\ldots ,\alpha_{s}),F),$$

$$M_{2}=M((h_{1},\ldots ,h_{n},\frac{1}{\alpha_{1}},\ldots ,\frac{1}{\alpha_{s}}),K[x_{1}]\ldots [x_{e}]).$$

Proof Let $\left(m_{1},\ldots ,m_{n})\in M(f,E\right)$, i.e., there is a $g\in {\mathrm{sconst}}_{G}\;E\setminus \{0\}$ with $\sigma (g)=f_{1}^{m_{1}}\ldots f_{n}^{m_{n}}\;g$. Hence by Corollary 6.9 it follows that $g=\overset{˜}{g}\;h$ with $\overset{˜}{g}\in F^{⁎}$ and $h\in K[x_{1}]\ldots {\left[x_{e}\right]}^{⁎}$. In particular, $\sigma (\overset{˜}{g})={\overset{˜}{f}}_{1}^{m_{1}}\ldots {\overset{˜}{f}}_{n}^{m_{n}}\;\lambda \;\overset{˜}{g}$ for $\lambda \in K^{⁎}$ being an rth root of unity. Hence we can take $m_{n+1},\ldots ,m_{n+s}\in N$ such that $\lambda =\alpha_{1}^{m_{n+1}}\ldots \alpha_{s}^{m_{n+s}}$. Consequently,

(34) $$\sigma (\overset{˜}{g})={\overset{˜}{f}}_{1}^{m_{1}}\ldots {\overset{˜}{f}}_{n}^{m_{n}}\alpha_{1}^{m_{n+1}}\ldots \alpha_{s}^{m_{n+s}}\;\overset{˜}{g},$$

which yields

(35) $$\sigma (h)=h_{1}^{m_{1}}\ldots h_{n}^{m_{n}}\alpha_{1}^{-m_{n+1}}\ldots \alpha_{s}^{-m_{n+s}}\;h.$$

Then (34) and (35) imply $(m_{1},\ldots ,m_{n+s})\in M_{1}\cap M_{2}$. Conversely, let $(m_{1},\ldots ,m_{n})\in M_{1}\cap M_{2}$. I.e., there are $m_{i}\in N$, $\overset{˜}{g}\in F^{⁎}$ and $h\in K[x_{1}]\ldots {\left[x_{e}\right]}^{⁎}$ s.t. (34) and (35) hold. Therefore $\sigma (\overset{˜}{g}\;h)=f_{1}^{m_{1}}\ldots f_{n}^{m_{n}}\;\overset{˜}{g}\;h$ which implies that $\left(m_{1},\ldots ,m_{n})\in M(f,E\right)$.  □

The following remarks are in place. By Corollary 4.3 it follows that ${\mathrm{sconst}}_{G}\;E\setminus \{0\}\leq E^{⁎}$ and thus M(f,E) in Lemma 6.10 has a Z-basis with rank ≤*n*. In particular, we can compute such a basis as follows. First note that both $M_{1}$ and $M_{2}$ given in Lemma 6.10 have Z-bases with rank ≤n+s: for $M_{1}$ this follows since F is a field. Moreover, if one takes $H=K[x_{1}]\ldots [x_{e}]\leq E$ and $H={\left(K^{⁎}\right)}_{K}^{H}$, it follows by Corollary 4.3 that ${\mathrm{sconst}}_{H}\;H\setminus \{0\}\leq H^{⁎}$ and thus a Z-basis exists with rank ≤n+s. Summarizing, we can determine a Z-basis of M(f,E) by using (33) if bases of $M_{1}$ and $M_{2}$ are available.

Example 6.11 Take the $\Pi \Sigma^{⁎}$-field (K(k),σ) over K=Q(ι) with σ(k)=k+1 and consider the *R*-extension (K(k)[x],σ) of (K(k),σ) with σ(x)=ιx and ord(x)=4 from Example 2.9. In order to obtain a degree bound in Example 7.6 below, we need a basis of M=M(f,K(k)[x]) with $f=(kx,-\frac{x}{k+1})$. Here we will apply Lemma 6.10. By Example 5.4.(3) we get ford(x)=8. With $u_{1}=1$ we determine r=8 by (29). We define $\overset{˜}{f_{1}}=k$, ${\overset{˜}{f}}_{2}=-1/(k+1)$ and $h_{1}=h_{2}=x$. All 8th roots of unity of K are generated by $\alpha_{1}=\iota$. For the activation of the above lemma, we have to determine a basis of $M_{1}=M(({\overset{˜}{f}}_{1},{\overset{˜}{f}}_{2},\alpha_{1}),K(k))=M((k,\frac{-1}{k+1},\iota ),K(k)$. Here we use, e.g., the algorithms worked out in Karr (1981) (this is the base case of our machinery, see Subsection 2.3.3) and obtain the basis {(1,1,2),(0,0,4)}. Moreover, we compute the basis {(1,1,0),(0,2,0),(0,0,1)} of $M_{2}=M((h_{1},h_{2},\alpha_{1}),K[x])=M((x,x,\iota ),K[x])$, for details see Example 6.14 below. Thus a basis of $M_{1}\cap M_{2}$ is {(1,1,2),(0,0,4)} and we get the basis {(1,1)} of *M*.

By assumption (i.e., the base case in our recursion) a basis of $M_{1}$ can be determined. The calculation of a Z-basis of $M_{2}$ can be accomplished by using the following proposition.

Proposition 6.12 *Let* (H,σ) *with* $H=K[x_{1}]\ldots [x_{e}]$ *be a simple R-extension of* (K,σ) *with a computable constant field* K *and given* $o_{i}=\mathrm{ord}(x_{i})$ *for* 1≤i≤e*. Define* $G={\left(K^{⁎}\right)}_{K}^{H}$ *and let* $f=(f_{1},\ldots ,f_{n})\in G^{n}$ *with given* $\lambda_{i}:=\mathrm{ord}(f_{i})>0$ *for* 1≤i≤n*. Then a basis of* M(f,H) *can be computed.*

Proof Define the finite sets

$$S:=\{(n_{1},\ldots ,n_{e})\in N^{e}|\;0\leq n_{i}<o_{i}\}\text{ and }\overset{˜}{M}:=\{(m_{1},\ldots ,m_{n})\in N^{n}|\;0\leq m_{i}<\lambda_{i}\}.$$

Then loop through all vectors $m=(m_{1}\ldots ,m_{n})\in \overset{˜}{M}$ and check if there is a $g\in H^{⁎}$ with $\sigma (g)=f_{1}^{m_{1}}\ldots f_{n}^{m_{n}}\;g$. More precisely, we can make the Ansatz $g=\sum_{i\in S}c_{i}\;x^{i}$ which leads to a linear system of equations in the $c_{i}$ with coefficients from K. Solving this system gives the solution space^9^ *L* and we can check if the considered **m** from $\overset{˜}{M}$ is contained in M(f,H). In this way we can generate the subset $M^{\prime }=\overset{˜}{M}\cap M(f,H)$. Denote by $b_{i}\in K^{n}$ the ith unit vector. We show that

(36) $$\mathrm{span}(M^{\prime }\cup \{\lambda_{1}\;b_{1},\ldots ,\lambda_{n}\;b_{n}\})=M(f,H).$$

Namely, since M(f,H) is a Z-module (see the remarks above Example 6.11) and since $\lambda_{i}\;b_{i}\in M(f,H)$, the left hand side is contained in the right hand side. Conversely, suppose that $\left(m_{1},\ldots ,m_{n})\in M(f,H\right)$. Then let $m_{i}^{\prime }=m_{i}\;\mathrm{mod}\;\lambda_{i}$, i.e., $0\leq m_{i}^{\prime }<\lambda_{i}$ with $m_{i}=m_{i}^{\prime }+z_{i}\;\lambda_{i}$ for some $z_{i}\in Z$. Thus $\left(m_{1},\ldots ,m_{n})=(m_{1}^{\prime },\ldots ,m_{n}^{\prime })+(\lambda_{1}\;z_{1},\ldots ,\lambda_{n}\;z_{n}\right)$ where $(m_{1}^{\prime },\ldots ,m_{n}^{\prime })\in \overset{˜}{M}$ and $\left(\lambda_{1}\;z_{1},\ldots ,\lambda_{n}\;z_{n})=z_{1}\;(\lambda_{1}\;b_{1})+\cdots +z_{n}\;(\lambda_{n}\;b_{n}\right)$. Consequently, $\left(m_{1},\ldots ,m_{n}\right)$ is an element of the left hand side of (36). Since the number of vectors of the span on the left hand side is finite, we can derive a Z-basis of (36).  □

Remark 6.13 A basis of M(f,H) can be obtained more efficiently as follows. We start with the Z-module which is given by the basis $B=\{\lambda_{1}\;b_{1},\ldots ,\lambda_{n}\;b_{n}\}$ where $b_{i}\in K^{n}$ is the *i*th unit vector. Now go through all elements from $\overset{˜}{M}$. Take the first element **m** from $\overset{˜}{M}$. If it is in span(B) (this can be easily checked), proceed to the next element. Otherwise, if it is an element from M(f,H) (for the check see the proof of Proposition 6.12), put it in *B* and transform the set again to a Z-basis. More precisely, if we compose the rows $b_{i}$ to a matrix, it should yield a matrix in Hermite normal form. In this way, the membership tests for span(B) can be carried out efficiently within the continuing calculation steps. We proceed until all elements of $\overset{˜}{M}$ are visited and update step by step *B* as described above. By construction we have that our span(B) equals the left hand side of (36) and thus equals M(f,H). We remark that *B* consists always of *n* linearly independent vectors. However, the Z-span is more and more refined.

Example 6.14 **Cont. Example 6.11**

Take the *R*-extension (K[x],σ) of (K,σ) with K=Q(ι), σ(x)=ιx and ord(x)=4. We calculate a basis of M(f,K[x]) with f=(x,x,ι) as presented in Remark 6.13. We start with {(4,0,0),(0,4,0),(0,0,4)} whose rows form a matrix in Hermite normal form. Now we go through all elements of $\overset{˜}{M}$, say in the order

$$\overset{˜}{M}=\{(1,0,0),(2,0,0),(3,0,0),(0,1,0),(0,2,0),(0,3,0),(0,0,1),\;\;(0,0,2),(0,0,3),(1,1,0),\ldots \}.$$

Since (1,0,0)∉span(B), we check if there is a g∈K[x]∖{0} with $\sigma (g)=x^{1}\;x^{0}\;\iota^{0}\;g$: this is not the case. We continue with (2,0,0). Here we have that (2,0,0)∉span(B). Now we check if there is a g∈K[x]∖{0} with $\sigma (g)=x^{2}\;x^{0}\;\iota^{0}\;g$. Plugging in $g=g_{0}+g_{1}\;x+g_{2}\;x^{2}+g_{3}\;x^{3}$ into $\sigma (g)=x^{2}\;g$ gives the constraint $(g_{0}-g_{2})x^{0}+\iota x(g_{1}+\iota g_{3})+x^{2}(-g_{0}-g_{2})+x^{3}(-g_{1}-ig_{3})=0$ which leads to the solution $g=x+\iota \;x^{3}$. A basis of span(B∪{(2,0,0)}) is {(2,0,0),(0,4,0),(0,0,4)}. Thus we update *B* to B={(2,0,0),(0,4,0),(0,0,4)}. We have (3,0,0)∉span(B), but there is no g∈K[x]∖{0} with $\sigma (g)=x^{3}\;g$. Similarly to (1,0,0), also (0,1,0) does not change *B*, and similarly to (2,0,0), (0,2,0) leads to the updated basis B={(2,0,0),(0,2,0),(0,0,4)}. (0,3,0) does not change *B*. However, for (0,0,1)∉span(B) we find g=x with $\sigma (g)=x^{0}\;x^{0}\;\iota^{1}\;g$ which yields B={(2,0,0),(0,2,0),(0,0,1)}. We have that (0,0,2),(0,0,3)∈span(B). Now we consider (1,1,0)∉span(B). We find $g=x+\iota \;x^{3}$ with $\sigma (g)=x^{2}\;g$ (as already above). Hence we update *B* to B={(1,1,0),(0,2,0),(0,1,0)} (where the rows form a matrix in Hermite normal form). As it turns out, no further element from $\overset{˜}{M}$ changes *B*. Thus the found *B* is a basis of M(f,K[x]).

Proof 6.15 **Theorem 2.26.(2)**

By Lemma 4.10 we can reorder the generators of the $R\Pi \Sigma^{⁎}$-extension such that $\left(\overset{¯}{E},\sigma \right)$ is an $F^{⁎}$-simple *R*-extension of (F,σ) and (E,σ) is a *G*-simple $\Pi \Sigma^{⁎}$-extension of $\left(\overset{¯}{E},\sigma \right)$ with $G={\left(F^{⁎}\right)}_{F}^{\overset{¯}{E}}$. Let $\overset{¯}{E}=F[x_{1}]\ldots [x_{e}]$ with $u_{i}$, $\alpha_{i}$ and $f\in G^{n}$ with ${\overset{˜}{f}}_{i}$ and $h_{i}$ as given in Lemma 6.10. By assumption we can compute a basis of $M_{1}$ as given in Lemma 6.10. Since Problem O is solvable in $K^{⁎}$, we can compute $o_{i}=\mathrm{ord}(x_{i})$ and $\lambda_{i}=\mathrm{ord}(u_{i})$ by Corollary 5.6.(4). Thus we can use Proposition 6.12 to compute a basis of $M_{2}$ as posed in Lemma 6.10, and we get a basis of (33). Summarizing, we can solve Problem PMT in $\left(\overset{¯}{E},\sigma \right)$ for *G*. In particular, ${\mathrm{sconst}}_{G}\;\overset{¯}{E}\setminus \{0\}\leq {\overset{¯}{E}}^{⁎}$ by Corollary 4.3. Hence by Theorem 6.1 we can solve Problem PMT for (E,σ) in $G_{\overset{¯}{E}}^{E}$. Since $G_{\overset{¯}{E}}^{E}={\left(F^{⁎}\right)}_{F}^{E}$ by Lemma 4.12, the theorem is proven.  □

To this end, we work out the following shortcut, resp. refined version of Theorem 2.12.(3).

Corollary 6.16 *Let* (F,σ) *be a strong constant-stable difference field with constant field* K*, and let* $G\leq F^{⁎}$ *with* ${\mathrm{sconst}}_{G}\;F\setminus \{0\}\leq F^{⁎}$*. Let* (H,σ) *with* $H=F[x_{1}]\ldots [x_{r}]$ *be a G-simple R-extension of* (F,σ) *and let* (E,σ) *be a* $G_{F}^{H}$*-simple* $\Pi \Sigma^{⁎}$*-extension of* (H,σ)*.*

(1) *If* $f\in G_{F}^{H}$ *with* ord(f)>0*, then* $f\in {\left(K^{⁎}\cap G\right)}_{F}^{H}$*.*
(2) $M(f,E)=M(f,K[x_{1}]\ldots [x_{r}])$ *for any* $f=(f_{1},\ldots ,f_{n})\in {\left(G_{F}^{H}\right)}^{n}$ *with* $\mathrm{ord}(f_{i})>0$*.*
(3) *Let* $\alpha \in G_{F}^{H}$ *with* ord(α)>0*. Then there is an R-extension* (E[t],σ) *of* (E,σ) *with* $\frac{\sigma (t)}{t}=\alpha$ *iff there is an R-extension* $\left(K[x_{1}]\ldots [x_{r}][t],\sigma \right)$ *of* $\left(K[x_{1}]\ldots [x_{r}],\sigma \right)$ *with* $\frac{\sigma (t)}{t}=\alpha$*.*

Proof (1) Let $f\in G_{F}^{H}$, i.e., $f=\alpha \;x_{1}^{m_{1}}\ldots x_{r}^{m_{r}}$ where α∈G and $m_{i}\in N$. With ord(f)>0 and Corollary 5.6.(3) we have that ord(α)>0. Since (F,σ) is strong constant-stable, $\alpha \in K^{⁎}$. Thus $\alpha \in K^{⁎}\cap G$ and hence $f\in {\left(K^{⁎}\cap G\right)}_{F}^{H}$.

(2) Let $f\in {\left(G_{F}^{H}\right)}^{n}$ be given as above. By part 1, $f_{i}=\alpha_{i}\;x_{1}^{m_{i,1}}\ldots x_{1}^{m_{i,r}}$ where the $\alpha_{i}\in K$ are roots of unity and $m_{i,j}\in N$. By Lemma 4.13 we may suppose that $E=H\langle t_{1}\rangle \ldots \langle t_{k}\rangle [s_{1}]\ldots [s_{e}]$ where the $t_{i}$ are Π-monomials and the $s_{i}$ are $\Sigma^{⁎}$-monomials. By Corollary 6.5 we have that $M(f,E)=M(f,H\langle t_{1}\rangle \ldots \langle t_{k}\rangle )$. Now let $\left(m_{1},\ldots ,m_{n})\in M(f,H\langle t_{1}\rangle \ldots \langle t_{k}\rangle \right)$. Then there is a $g\in H\langle t_{1}\rangle \ldots \langle t_{k}\rangle \setminus \{0\}$ with

(37) σ(g)=ug

for some $u=a\;x_{1}^{\mu_{1}}\ldots x_{r}^{\mu_{r}}$ with $\mu_{i}\in N$ and with *a* being a root of unity from K. By Corollary 5.6.(3) we get μ:=ord(u)>0; in addition we have that $\mu^{\prime }=\mathrm{ford}(u)>0$. By Theorem 4.9 it follows that $g=q\;t_{1}^{\nu_{1}}\ldots t_{k}^{\nu_{k}}$ with $q\in {\mathrm{sconst}}_{G}\;H\setminus \{0\}$ and $\nu_{i}\in Z$. Since $u^{\mu }=1$, it follows with (37) that $\sigma (g^{\mu })=g^{\mu }$. Now suppose that *g* depends on $t_{m}$ with 1≤m≤k being maximal. Then $g^{\mu }$ depends also on $t_{m}$ which contradicts to $\mathrm{const}\;H\langle t_{1}\rangle \ldots \langle t_{k}\rangle =\mathrm{const}\;H$. Consequently $g=q\in {\mathrm{sconst}}_{G}\;H\setminus \{0\}$. By Corollary 6.9 it follows that $g=\overset{˜}{g}\;h$ with $h\in K[x_{1}]\ldots {\left[x_{r}\right]}^{⁎}$ and $\overset{˜}{g}\in F^{⁎}$ with σ(h)=λuh where $\lambda \in K^{⁎}$ is a root of unity. Recall that $\mu^{\prime }=\mathrm{ford}(u)>0$ and hence $\mu^{\prime \prime }:=\mathrm{lcm}(\mu^{\prime },\mathrm{ord}(\lambda ))>0$. Since $\sigma^{\mu^{\prime \prime }}(h)=h$ and (F,σ) is constant-stable, it follows that $h\in K^{⁎}$. Therefore $g\in K[x_{1}]\ldots {\left[x_{r}\right]}^{⁎}$. Summarizing, $\left(m_{1},\ldots ,m_{n})\in M(f,K[x_{1}]\ldots [x_{r}]\right)$ and we conclude that $M(f,E)\subseteq M(f,K[x_{1}]\ldots [x_{r}])$. The other direction is immediate.

(3) The third part follows by parts 1 and 2 of the corollary and Theorem 3.17.  □

## 7 The algorithmic machinery III: Problem PFLDE

We aim at proving Theorems 2.23.(2) and 2.26.(3), i.e., providing recursive algorithms that reduce Problem PFLDE from a given $R\Pi \Sigma^{⁎}$-extension to its ground ring (resp. field). If we are considering single-rooted $R\Pi \Sigma^{⁎}$-extensions (Theorem 2.23.(2)), we rely heavily on the fact that for a given difference ring (G,σ) with constant field K and given group $G\leq G^{⁎}$ we have that ${\mathrm{sconst}}_{G}(G,\sigma )\setminus \{0\}\leq G^{⁎}$. This property allows us to assume that for any $f\in G^{n}$ and any u∈G the K-vector space V=V(u,f,(G,σ)) has a basis with dimension ≤n+1; see Lemma 2.17. In particular, our reduction algorithm is based on the assumption that there are algorithms available that solve Problems PFLDE and PMT in (G,σ) for *G*. For general simple $R\Pi \Sigma^{⁎}$-extensions over a strong constant-stable difference field (G,σ) (Theorem 2.26.(3)) we need stronger properties: all what we stated above should hold not only for (G,σ) but must hold for $\left(G,\sigma^{l}\right)$ with l≥1. For the currently explored difference fields (G,σ) with these properties we refer to Subsection 2.3.3.

### 7.1 A reduction strategy for $\Pi \Sigma^{⁎}$-extensions

In this subsection we present a reduction method for $\Pi \Sigma^{⁎}$-extensions which can be summarized with the following theorem.

Theorem 7.1 *Let* (A,σ) *be a computable difference ring and let* $G\leq A^{⁎}$ *with* ${\mathrm{sconst}}_{G}\;A\setminus \{0\}\leq A^{⁎}$*. Let* (A〈t〉,σ) *be a G-simple* $\Pi \Sigma^{⁎}$*-extension of* (A,σ)*.*

(1) *If t is a* $\Sigma^{⁎}$*-monomial and Problem PFLDE is solvable in* (A,σ) *for G, then Problem PFLDE is solvable in* (A〈t〉,σ) *for* $G_{A}^{A\langle t\rangle }$*.*
(2) *If t is a* Π*-monomial and Problems PFLDE and PMT are solvable in* (A,σ) *for G, then Problem PFLDE is solvable in* (A〈t〉,σ) *for* $G_{A}^{A\langle t\rangle }$*.*

In the following let (A〈t〉,σ)≥(A,σ) be a $\Pi \Sigma^{⁎}$-extension as given in the theorem with σ(t)=αt+β where α∈G and β=0, or α=1 and β∈A. Furthermore, we define $\overset{˜}{G}=G_{A}^{A\langle t\rangle }$ and suppose that we are given a $u\in \overset{˜}{G}$, i.e.,

(38) $$u=v\;t^{m},\;\text{with }v\in G,\;m\in Z,$$

and an $f=(f_{1},\ldots ,f_{n})\in {\overset{˜}{G}}^{n}$. By Theorem 3.20 we have that ${\mathrm{sconst}}_{\overset{˜}{G}}\;A\langle t\rangle \setminus \{0\}\leq A{\langle t\rangle }^{⁎}$ and hence by Lemma 2.17 a basis of V(u,f,A〈t〉) with dimension ≤n+1 exists.

Subsequently, we will prove Theorem 7.1, i.e., we will work out a reduction strategy that provides a basis of V(u,f,A〈t〉) under the assumption that one can solve Problem PFLDE in (A,σ) for *G* if *t* is a $\Sigma^{⁎}$-monomial, resp. Problems PMT and PFLDE in (A,σ) for *G* if *t* is a Π-monomial. The two main steps of this reduction will be described in the following two Subsections 7.1.1 and 7.1.2.

#### 7.1.1 Degree bounds

The first essential step is to search for degree bounds: we will determine a,b∈Z such that

(39) $$V(u,f,A{\langle t\rangle }_{a,b})=V(u,f,A\langle t\rangle )$$

holds; for the definition of the truncated set of (Laurent) polynomials see (18). For technical reasons we also require that the constraint

(40) $$\max(b,b+m)\geq \overset{˜}{b}$$

holds where *m* and $\overset{˜}{b}$ are given by (38) and

(41) $$\overset{˜}{b}=\max(\deg(f_{1})\ldots ,\deg(f_{n})).$$

The recovery of these bounds (see Lemmas 7.2 and 7.5 below) is based on generalizations of ideas given in Karr (1981); for further details and proofs in the setting of difference fields see also Schneider (2004a, 2005a).

If *t* is a $\Sigma^{⁎}$-monomial, then A〈t〉=A[t] forms a polynomial ring, α=1 and $\overset{˜}{G}=G$; in particular we have m=0 in (38). In this case, we can utilize the following lemma.

Lemma 7.2 *Let* (A[t],σ) *be a* $\Sigma^{⁎}$*-extension of* (A,σ) *and let* $G\leq A^{⁎}$ *such that* ${\mathrm{sconst}}_{G}\;A\setminus \{0\}\leq A^{⁎}$ *holds. Let* f∈A[t] *and* u∈G*. Then any solution* g∈A[t] *of* σ(g)−ug=f *is bounded by* deg⁡(g)≤max⁡(deg⁡(f)+1,0)*.*

Proof Suppose there is a g∈A[t] with deg⁡(g)>max⁡(deg⁡(f)+1,0). Thus by Lemma 3.8 there is a γ∈A with σ(γ)−γ=σ(t)−t which contradicts to Theorem 2.12.(1).  □

Thus we can set a=0 and $b=\max(\overset{˜}{b}+1,0)$ to guarantee that (39) and (40) hold.

Example 7.3 **Cont. Example 2.15**

Consider the $\Sigma^{⁎}$-extension (A[S],σ) of (A,σ) with A=Q(k)[x][y][s] and $\sigma (S)=S+\frac{-x\;y}{k+1}$ from Example 2.15.(2). As stated in Example 2.15.(3), we want to determine a g∈A[S] with σ(g)−g=f where $f=y\;k^{2}\;s$, i.e., we want to find a basis of V(1,f,A[S]) with $f=(k^{2}sy)\in A{\left[S\right]}^{1}$. Using Lemma 7.2 it follows that deg⁡(g)≤1. Consequently, $V(1,f,A[S])=V(1,f,A{\left[S\right]}_{0}^{1})$. Using our methods below (see Example 7.8) we get the basis {(1,g),(0,1)} with *g* as given in (14).

If *t* is a Π-monomial, then $A\langle t\rangle =A[t,\frac{1}{t}]$ is a ring of Laurent polynomials and β=0.

First suppose that u∉A, i.e., m∈Z∖{0} as given in (38).

If $f_{i}=0$ for all *i*, it is easy to see that V(u,f,A〈t〉)=V(u,f,{0}), i.e., a=0 and b=−1 fulfill the properties (39) and (40).

Otherwise, if not all $f_{i}$ are 0, we can use the following fact; the proof is left to the reader.

Lemma 7.4 *Let* (A〈t〉,σ) *be a* Π*-extension of* (A,σ)*. Let* $v\in A^{⁎}$*,* m∈Z∖{0}*,* $f=\sum_{i=\lambda }^{\mu }f_{i}\;t^{i}\in A\langle t\rangle$ *with* λ,μ∈Z *and* $g=\sum_{i=\overset{˜}{\lambda }}^{\overset{˜}{\mu }}g_{i}t^{i}\in A\langle t\rangle$ *with* $\overset{˜}{\lambda },\overset{˜}{\mu }\in Z$ *and* $g_{\overset{˜}{\lambda }}\neq 0\neq g_{\overset{˜}{\mu }}$ *such that* $\sigma (g)-v\;t^{m}\;g=f$*. Then* $\max(\lambda ,\lambda -m)\leq \overset{˜}{\lambda }$ *and* $\overset{˜}{\mu }\leq \min(\mu ,\mu -m)$*.*

Namely, define

$$\overset{˜}{a}=\min(\mathrm{ldeg}(f_{1})\ldots ,\mathrm{ldeg}(f_{n})).$$

Note that in this scenario we have that $\overset{˜}{a},\overset{˜}{b}\in Z$; for the definition of $\overset{˜}{b}$ see (41). Hence by setting $a=\overset{˜}{a}$ and $b=\overset{˜}{b}$, we can conclude with Lemma 7.4 that (39) and (40) hold.

What remains to consider is the case u∈G with m=0. Here we utilize

Lemma 7.5 *Let* (A〈t〉,σ) *be a* Π*-extension of* (A,σ) *with* $G\leq A^{⁎}$ *where* ${\mathrm{sconst}}_{G}\;A\setminus \{0\}\leq A^{⁎}$ *and* α=σ(t)/t∈G*. Let* u∈G*,* $f=\sum_{i=\lambda }^{\mu }f_{i}\;t^{i}\in A\langle t\rangle$ *and* $g=\sum_{i=\overset{˜}{\lambda }}^{\overset{˜}{\mu }}g_{i}t^{i}\in A\langle t\rangle$ *with* $g_{\overset{˜}{\lambda }}\neq 0\neq g_{\overset{˜}{\mu }}$ *and*

(42) σ(g)−ug=f.

*If there is a* ν∈Z *with*

(43) $$\sigma (\gamma )=\alpha^{-\nu }\;u\;\gamma$$

*for some* $\gamma \in {\mathrm{sconst}}_{G}\;A\setminus \{0\}$*, then ν is uniquely determined and we have that* $\min(\lambda ,\nu )\leq \overset{˜}{\lambda }$ *and* $\overset{˜}{\mu }\leq \max(\mu ,\nu )$*. If there is not such a ν, we have that* $\lambda \leq \overset{˜}{\lambda }$ *and* $\overset{˜}{\mu }\leq \mu$*.*

Proof Suppose there is a ν∈Z with (43) for some $\gamma \in {\mathrm{sconst}}_{G}\;A\setminus \{0\}$. Take in addition, $\overset{˜}{\nu }\in Z$ such that $\sigma (\overset{˜}{\gamma })=\alpha^{-\overset{˜}{\nu }}\;u\overset{˜}{\gamma }$ holds for some $\overset{˜}{\gamma }\in {\mathrm{sconst}}_{G}\;A\setminus \{0\}$. Then $\sigma (\gamma /\overset{˜}{\gamma })=\alpha^{\overset{˜}{\nu }-\nu }\;\gamma /\overset{˜}{\gamma }$. Since *t* is a Π-monomial it follows by Theorem 2.12.(2) that $\nu =\overset{˜}{\nu }$, i.e., *ν* is uniquely determined. Now suppose that there is an *i* with $g_{i}\neq 0$ where we have i<min⁡(λ,ν) or i>max⁡(μ,ν). Then by coefficient comparison in (42) we get $\sigma (g_{i})=u\;\alpha^{-i}\;g_{i}$ with $g_{i}\in {\mathrm{sconst}}_{G}\;A\setminus \{0\}$. Consequently ν=i, a contradiction. Otherwise, suppose that there is not such a ν∈Z. Then by the same arguments it follows that $\overset{˜}{\lambda }<\lambda$ or $\overset{˜}{\mu }>\mu$ is not possible, i.e., $\overset{˜}{\lambda }\geq \lambda$ and $\overset{˜}{\mu }\leq \mu$. This completes the proof.  □

Therefore we derive the desired bounds as follows. First, solve Problem PMT and compute a basis of M((α,u),A). Then given a basis, we can decide constructively if there is a ν∈Z such that (43) holds. If yes, take the uniquely determined *ν* and we can take $a=\min(\overset{˜}{a},\nu )$ and $b=\max(\overset{˜}{b},\nu )$ to obtain (39) and (40). Otherwise, if there is not such a *ν*, we can set $a=\min(\overset{˜}{a},0)$ and $b=\max(\overset{˜}{b},-1)$.

Example 7.6 **Cont. Example 2.18**

Take the difference field (K(k)[x]〈t〉,σ) with α=σ(t)/t=xk defined in Example 2.18. In order to find the identity (7), we need a basis of V=V(u,(0),K(k)[x]〈t〉) with $u=\frac{-x}{k+1}\in G:={\left(K{\left(k\right)}^{⁎}\right)}_{K(k)}^{K(k)[x]\langle t\rangle }$; note that $G\leq K(k)[x]{\langle t\rangle }^{⁎}$ with ${\mathrm{sconst}}_{G}\;K(k)[x]\langle t\rangle \setminus \{0\}\leq K(k)[x]{\langle t\rangle }^{⁎}$. In this setting we apply Lemma 7.5. I.e., we compute a basis of $M((\alpha ,u),K(k)[x])=M((k\;x,\frac{-x}{\left(k+1\right)}),K(k)[x])$. As worked out in Example 6.11, a basis is {(1,1)}. Thus we find ν=−1 such that there is a g∈K(k)[x]∖{0} with (43). We conclude that $V=V(u,(0),K(k)[x]{\langle t\rangle }_{-1}^{-1})$. Using our methods below (see Example 7.7) we arrive at the basis $\left(0,x(\iota +x^{2})/k/t),(1,0)\right\}$ of *V*.

Summarizing, we obtain bounds a,b∈Z such that (39) and (40) hold. For Π-monomials we rely on the extra assumption that Problem PMT is solvable in (A,σ) for *G*.

#### 7.1.2 Degree reduction

The following degree reduction has been introduced in Karr (1981) in the setting of difference fields. Subsequently, we present the basic ideas in the setting of difference rings; further technical details can be found in Schneider (2001, Thm. 3.2.2) and Schneider (2005d, 2015).

We want to determine all $c_{1},\ldots ,c_{n}\in K=\mathrm{const}\;A$ and $g_{i}\in A$ in $g=\sum_{i=a}^{b}g_{i}\;t_{i}$ such that the following parameterized equation holds:

(44) $$\sigma (g)-u\;g=c_{1}\;f_{1}+\cdots +c_{n}\;f_{n}.$$

If b<a, we are in the base case: g=0 and a basis of V(u,f,A〈t〉)=V(u,f,{0}) can be determined by linear algebra.

Otherwise, we continue as follows. Due to (40), it follows that λ:=max⁡(b,b+m) is the highest possible exponent in (44). Let ${\overset{˜}{f}}_{i}$ be the coefficient of the term $t^{\lambda }$ in $f_{i}$. Then by coefficient comparison w.r.t. $t^{\lambda }$ in (44) we get the following constraints:

Ifm>0, (45) $$-v\;g_{b}=c_{1}\;\overset{˜}{f_{1}}+\cdots +c_{n}\;{\overset{˜}{f}}_{n};$$
ifm=0, (46) $$\alpha^{b}\;\sigma (g_{b})-v\;g_{b}=c_{1}\;\overset{˜}{f_{1}}+\cdots +c_{n}\;{\overset{˜}{f}}_{n};$$
ifm<0, (47) $$\alpha^{b}\;\sigma (g_{b})=c_{1}\;\overset{˜}{f_{1}}+\cdots +c_{n}\;{\overset{˜}{f}}_{n}.$$

For the cases m>0 and m<0 one can easily determine a basis of the K-vector spaces $\left\{(c_{1},\ldots ,c_{n},g_{m})|\;\text{(45) holds}\right\}$ and $\left\{(c_{1},\ldots ,c_{n},g_{m})|\;\text{(47) holds}\right\}$ by linear algebra. Moreover, if m=0, Eq. (46) can be written in the form $\sigma (g_{b})-v\;\alpha^{-b}\;g_{b}=c_{1}\;\overset{˜}{f_{1}}\alpha^{-b}+\cdots +c_{n}\;{\overset{˜}{f}}_{n}\alpha^{-b}$ where $v\;\alpha^{-b}\in G$ and ${\overset{˜}{f}}_{i}\alpha^{-b}\in A$. Thus a basis of

(48) $$V(v\;\alpha^{-b},(\overset{˜}{f_{1}}\;\alpha^{-b},\ldots ,{\overset{˜}{f}}_{n}\;\alpha^{-b}),A)$$

can be determined under our assumption that one can solve Problem PFLDE in (A,σ) for *G*. Now we plug in this partial solution (i.e., the possible leading coefficient $g_{b}$ with the corresponding linear combinations of the $f_{i}$), and end up at a new first-order parameterized difference equation where the highest possible coefficient is λ−1. In other words, we reduced the problem by *degree reduction*. We continue to search for the next highest coefficient $g_{b-1}$. Hence we proceed recursively by updating λ→λ−1 and b→b−1 and determine a basis of the reduced problem (with highest degree λ−1). Finally, given a basis of this solution space and given the basis of (48), one can determine a basis of $V(u,f,A{\langle t\rangle }_{a,b})$; for further technical details we refer to Karr (1981, Thm. 12) or Schneider (2015, Section 3.1).

Summarizing, solving various instances of Problem PFLDE with the degree reductions b→b−1→⋯→a−1 leads to the base case and we eventually produce a basis of V(u,f,A〈t〉). This concludes the proof of Theorem 7.1.

Example 7.7 **Cont. Example 7.6**

We know that $g=g_{-1}t^{-1}$. Plugging in *g* into $\sigma (g)+\frac{x}{k+1}=0$ yields $\sigma (g_{-1})+\frac{x^{2}\;k}{k+1}\;g_{-1}=0$. Therefore we look for a basis of $V(\frac{-x^{2}\;k}{k+1},(0),K(k)[x])$. By using the algorithms presented in Subsection 7.2 we get the basis $\left\{(1,x(\iota +x^{2})/k),(0,1)\right\}$. This finally gives the basis $\left(0,x(\iota +x^{2})/k/t),(1,0)\right\}$ of $V(\frac{-x}{k+1},(0),K(k)[x]\langle t\rangle )$.

Example 7.8 **Cont. Example 7.3**

We want to find a basis of $V=V(1,f,A{\left[S\right]}_{0}^{1})$ with A=Q(k)[x][y][s] and $f=(y\;k^{2}\;s)$. Hence we make the Ansatz $(c_{1},g_{0}+g_{1}\;S)\in V$ with the indeterminates $c_{1}\in Q$ and $g_{0},g_{1}\in A$ such that

(49) $$\sigma (g_{0}+g_{1}\;S)-(g_{0}+g_{1}\;S)=c_{1}\;y\;k^{2}\;s$$

holds. Doing coefficient comparison w.r.t. $S^{1}$ yields the constraint $\sigma (g_{1})-g_{1}=c_{1}\;0$; compare (46). Thus we get all solutions by determining a basis of $V(1,\overset{˜}{f},Q(k)[x][s])$ with $\overset{˜}{f}=(0)\in A^{1}$. In this particular instance, the Q-basis {(1,0),(0,1)} is immediate utilizing the fact that the constants are precisely Q. Summarizing, the solutions are $(c_{1},g_{1})\in Q^{2}$. Consequently, our Ansatz can be refined with $(c_{1},g_{0}+c_{2}\;S)\in V$ where $c_{1}\in Q$, $c_{2}(=g_{1})\in Q$ and $g_{0}\in A$ such that $\sigma (g_{0}+c_{2}\;S)-(g_{0}+c_{2}\;S)=c_{1}\;k^{2}sy$ holds. Bringing the $c_{2}\;S$ part to the right hand side yields the new equation^10^

(50) $$\sigma (g_{0})-g_{0}=c_{1}\;y\;k^{2}\;s-c_{2}\;h$$

with $h=\sigma (S)-S=\frac{-x\;y}{k+1}\in A$. In other words, we need a basis of V(1,h,A) with $h=(y\;k^{2}\;s,\frac{x\;y}{k+1})\in A^{2}$. Now we apply again the reduction method, but this time in the smaller ring A without the $\Sigma^{⁎}$-monomial *S*. We skip all the details, but refer to a particular subproblem that we will consider in Example 7.13. Finally, we get the basis

$$\left\{(0,0,1),(1,\frac{1}{2},(\frac{1}{4}(1-2k)-\frac{1}{4}x)y+s(\frac{1}{2}(k-1)(k+1)x-\frac{1}{2}(k-2)k)y\right\}$$

of V(1,h,A). Thus we can reconstruct the basis {(1,g),(0,1)} of *V* with *g* given in (14).

Note that the reduction of Theorem 7.1 simplifies to Karr's field version given in Karr (1981) if one specializes A to a field and sets $G=A^{⁎}=A\setminus \{0\}$. However, the presented version works not only for a field, but for any difference ring (A,σ) as specified in Theorem 7.1. Subsequently, we will exploit this enhancement in order to treat (nested) *R*-extensions.

### 7.2 A reduction strategy for *R*-extensions and thus for $R\Pi \Sigma^{⁎}$-extensions

In order to treat simple and single-rooted $R\Pi \Sigma^{⁎}$-extensions (Theorem 2.23.(2)), we utilize the following proposition.

Proposition 7.9 *Let* (A,σ) *be a computable difference ring with* $G\leq A^{⁎}$ *and* ${\mathrm{sconst}}_{G}\;A\setminus \{0\}\leq A^{⁎}$*. Let* (A[t],σ) *be an R-extension of* (A,σ) *of given order d with* $\frac{\sigma (t)}{t}\in G$*.*

*Then Problem PFLDE is solvable in* (A[t],σ) *for G if it is solvable in* (A,σ) *for G.*

Proof The proof follows by an adapted degree reduction presented in the proof of Theorem 7.1; see Subsection 7.1.2. Let u∈G and $f=(f_{1},\ldots ,f_{n})\in A{\left[t\right]}^{n}$. By definition, it follows that a solution g∈A[t] and $c_{1},\ldots ,c_{n}\in K=\mathrm{const}\;A$ of (44) is of the form $g=\sum_{i=a}^{b}g_{i}t^{i}$ with a:=0 and b:=d−1. Thus the bounds are immediate (under the assumption that *d* has been determined; see Section 5). Since $\sum_{i=0}^{d-1}h_{i}\;t^{i}=\sum_{i=0}^{d-1}{\overset{¯}{h}}_{i}\;t^{i}$ iff $h_{i}={\overset{¯}{h}}_{i}$, we can activate the degree reduction as outlined in Subsection 7.1.2. Namely, by coefficient comparison of the highest term we always enter in the case (46) (note that m=0 in (38)). By assumption we can solve Problem PFLDE in (A,σ) for *G* and thus we can determine a basis of (48). By recursion we finally obtain a basis of V(u,f,A[t]).  □

Proof 7.10 **Theorem 2.23.(2)**

Since Problem PFLDE is solvable in (G,σ) for *G*, it follows by iterative applications of Theorem 7.1 and Corollary 4.6.(1) that Problem PFLDE is solvable in (H,σ) for $\overset{˜}{G}$ with $H=G\langle t_{1}\rangle \ldots \langle t_{r}\rangle$ and that ${\mathrm{sconst}}_{\overset{˜}{G}}\;H\setminus \{0\}\leq H^{⁎}$. Thus by iterative applications of Propositions 7.9 and 3.23 we conclude that Problem PFLDE is solvable in $\left(\overset{¯}{H},\sigma \right)$ for $\overset{˜}{G}$ with $\overset{¯}{H}=H\langle x_{1}\rangle \ldots \langle x_{u}\rangle$ and that ${\mathrm{sconst}}_{\overset{˜}{G}}\;\overset{¯}{H}\setminus \{0\}\leq {\overset{¯}{H}}^{⁎}$. Finally, by applying iteratively Theorem 7.1 and Corollary 4.6.(1) it follows that Problem PFLDE is solvable in (E,σ) for $\overset{˜}{G}$. Note that in Proposition 7.9 we have to know the values $\mathrm{ord}(x_{i})=\mathrm{ord}(\alpha_{i})$ with $\alpha_{i}\in G$ (either as input or by computing them first by solving instances of Problem O in *G*).  □

Finally, we present the underlying reduction method for simple $R\Pi \Sigma^{⁎}$-extensions (Theorem 2.26.(3)) which is based on the following lemma and proposition.

Lemma 7.11 *Let* (A,σ) *be a difference ring,* f∈A*,* $u\in A^{⁎}$ *and* λ∈N∖{0}*. Then* σ(g)−ug=f *implies that*

(51) $$\sigma^{\lambda }(g)-u_{\left(\lambda \right)}\;g=\sum_{j=0}^{\lambda -1}\frac{u_{\left(\lambda \right)}}{u_{\left(j+1\right)}}\;\sigma^{j}(f).$$

Proof From σ(g)−ug=f we get $\sigma^{j+1}(g)-\sigma^{j}(u)\sigma^{j}(g)=\sigma^{j}(f)$ for all j∈N. Multiplying it with $u_{\left(\lambda \right)}/u_{\left(j+1\right)}$ yields $\frac{u_{\left(\lambda \right)}}{u_{\left(j+1\right)}}\sigma^{j+1}(g)-\frac{u_{\left(\lambda \right)}}{u_{\left(j\right)}}\sigma^{j}(g)=\frac{u_{\left(\lambda \right)}}{u_{\left(j+1\right)}}\sigma^{j}(f)$. Summing this equation over *j* from 0 to λ−1 produces (51).  □

Proposition 7.12 *Let* (A,σ) *be a constant-stable and computable difference ring with constant field* K*. Let* $G\leq A^{⁎}$ *be closed under σ with* ${\mathrm{sconst}}_{G}(A,\sigma^{l})\setminus \{0\}\leq A^{⁎}$ *for all* l>0*. Let* (E,σ) *with* $E=A\langle x_{1}\rangle \ldots \langle x_{r}\rangle$ *be a G-simple R-extension of* (A,σ) *where* $\mathrm{ord}(x_{i})>0$ *and* $\mathrm{per}(x_{i})>0$ *for* 1≤i≤r *are given and where* ${\mathrm{sconst}}_{\left(G_{A}^{E}\right)}\;E\setminus \{0\}\leq E^{⁎}$*.*

*If Problem PFLDE is solvable in* $\left(G,\sigma^{l}\right)$ *for G for all* l>0*, it is solvable in* (E,σ) *for* $G_{A}^{E}$*.*

Proof Let K=constA, let $E=A\langle x_{1}\rangle \ldots \langle x_{r}\rangle$, let $f=(f_{1},\ldots ,f_{n})\in E^{n}$ and let $u=v\;x_{1}^{m_{1}}\ldots x_{r}^{m_{r}}\in G_{A}^{E}$ with v∈G and $m_{i}\in N$. We will present a reduction method to obtain a basis of V(u,f,E). Set $\alpha :=x_{1}^{m_{1}}\ldots x_{r}^{m_{r}}$. Then by Lemma 5.2 it follows that ord(α)>0 can be computed by the given values of $\mathrm{ord}(x_{i})$ with 1≤i≤r. Hence we can activate Lemma 5.3.(4) and can compute ford(α)>0. Now take

(52) $$\lambda =\mathrm{lcm}(\mathrm{ford}(\alpha ),\mathrm{per}(x_{1}),\ldots ,\mathrm{per}(x_{r})).$$

Thus we have that^11^ $\alpha_{\left(\lambda \right)}=1\text{ and }\sigma^{\lambda }(x_{i})=x_{i}$ for all 1≤i≤r. Finally, define

(53) $$w:=u_{\left(\lambda \right)}={\left(\alpha \;v\right)}_{\left(\lambda \right)}=v_{\left(\lambda \right)}\in G.$$

Now let $\left(c_{1},\ldots ,c_{n},g)\in V(u,f,E\right)$, i.e., we have that (44). Thus Lemma 7.11 yields

(54) $$\sigma^{\lambda }(g)-w\;g=c_{1}\;{\overset{˜}{f}}_{1}+\cdots +c_{n}\;{\overset{˜}{f}}_{n}$$

with

(55) $${\overset{˜}{f}}_{i}=\sum_{j=0}^{\lambda -1}\frac{u_{\left(\lambda \right)}}{u_{\left(j+1\right)}}\sigma^{j}(f_{i}).$$

Hence V(u,f,E) is a subset of

(56) $$\overset{˜}{V}=\{(c_{1},\ldots ,c_{n},g)\in K^{n}\times E|\;\text{(54) holds}\}.$$

Note that $\overset{˜}{V}$ is a K-subspace of $K^{n}\times E$. Thus V(u,f,E) is a subspace of $\overset{˜}{V}$ over K. First, we show that $\overset{˜}{V}$ has a finite basis and show how one can compute it. For this task define $S:=\{(n_{1},\ldots ,n_{r})\in N^{r}|\;0\leq n_{i}<\mathrm{ord}(x_{i})\}$. Write $g=\sum_{i\in S}g_{i}x^{i}$ and ${\overset{˜}{f}}_{j}=\sum_{i\in S}{\overset{˜}{f}}_{j,i}\;x^{i}$ in multi-index notation. Since $\sigma^{\lambda }(x_{i})=x_{i}$, it follows by coefficient comparison that for i∈S we have that

$$\sigma^{\lambda }(g_{i})-w\;g_{i}=c_{1}\;{\overset{˜}{f}}_{1,i}+\cdots +c_{n}\;{\overset{˜}{f}}_{n,i}.$$

By assumption, ${\mathrm{sconst}}_{G}(A,\sigma^{\lambda })\setminus \{0\}\leq A^{⁎}$. In particular, since (A,σ) is constant-stable, we have that $\mathrm{const}(A,\sigma^{\lambda })=K$. Thus with our w∈G and ${\overset{˜}{f}}_{i}=({\overset{˜}{f}}_{1,i},\ldots ,{\overset{˜}{f}}_{n,i})\in A^{n}$ we can solve Problem PFLDE in $\left(A,\sigma^{\lambda }\right)$ with constant field K. Hence we get for all i∈S the bases for

(57) $$V_{i}=V(w,{\overset{˜}{f}}_{i},(A,\sigma^{\lambda })).$$

Note that by construction it follows that $\overset{˜}{V}$ from (56) is given by

(58) $$\overset{˜}{V}=\{(c_{1},\ldots ,c_{n},\sum_{i\in S}g_{i}\;x^{i})|\;(c_{1},\ldots ,c_{n},g_{i})\in V_{i}\}.$$

Thus by linear algebra we get a basis of (58), say $b_{1},\ldots ,b_{s}\in K^{n}\times E$. Recall that V(u,f,(E,σ)) is a K-subspace of (58). To this end, we make the Ansatz $(c_{1},\ldots ,c_{n},g)=d_{1}\;b_{1}+\cdots +d_{s}\;b_{s}$ for indeterminates $d_{1},\ldots ,d_{s}\in K$ and plug in the generic solution into (44). This yields another linear system with unknowns $\left(d_{1},\ldots ,d_{s}\right)$. Solving this system enables one to derive a basis of V(u,f,E).  □

Example 7.13 **Cont. Example 7.8**

In order to compute a basis of V(1,h,A) in Example 7.8, the recursive reduction enters in the following subproblem. We are given the *R*-extension (Q(k)[x],σ) of (Q(k),σ) with σ(x)=−x and need a basis of V(x,f,Q(k)[x]) with $f=(f_{1},f_{2},f_{3})=(\frac{(k^{2}-1)x}{2k}+\frac{-k^{2}-2k}{2k},-\frac{x}{k},0)$. By Example 5.4.(2) we get per(x)=2 and ford(x)=4. Hence using (52) we determine λ=lcm(ford(x),per(x))=4. Using (55) with u=x yields $\left({\overset{˜}{f}}_{1},{\overset{˜}{f}}_{2},{\overset{˜}{f}}_{3})=(-\frac{2k^{2}+4k+1}{k(k+2)}-\frac{x}{\left(k+1)(k+3\right)},-\frac{2x}{\left(k+1)(k+3\right)}-\frac{2}{k(k+2)},0\right)$. Next we write the entries in multi-index notation. Namely, with $S=\{(0),(1)\}\subseteq N^{1}$ we get

$${\overset{˜}{f}}_{\left(0\right)}=({\overset{˜}{f}}_{1,(0)},{\overset{˜}{f}}_{2,(0)},{\overset{˜}{f}}_{3,(0)})=(-\frac{2k^{2}+4k+1}{k(k+2)},-\frac{2}{k(k+2)},0)$$

$${\overset{˜}{f}}_{\left(1\right)}=({\overset{˜}{f}}_{1,(1)},{\overset{˜}{f}}_{2,(1)},{\overset{˜}{f}}_{3,(1)})=(-\frac{1}{\left(k+1)(k+3\right)},-\frac{2}{\left(k+1)(k+3\right)},0)$$

with $f=\sum_{(m)\in S}{\overset{˜}{f}}_{\left(m\right)}x^{m}={\overset{˜}{f}}_{\left(0\right)}+{\overset{˜}{f}}_{\left(1\right)}\;x$. Following (53) we get w=1 and we have to compute bases of (57) with i∈S. Here we obtain the basis $\left\{(0,0,1,0),(1,-\frac{1}{2},0,-k/2)\right\}$ of $V_{\left(0\right)}=V(1,{\overset{˜}{f}}_{\left(0\right)},(Q(k),\sigma^{4}))$ and the basis $\left\{(-1,\frac{1}{2},0,0),(0,0,0,1),(0,0,1,0)\right\}$ of $V_{\left(1\right)}=V(1,{\overset{˜}{f}}_{\left(0\right)},(Q(k),\sigma^{4}))$. Therefore a basis of

$$\overset{˜}{V}=\{(c_{1},c_{2},c_{3},g)\in Q^{3}\times Q(k)[x]|\sigma^{4}(g)-g=c_{1}\;\overset{˜}{f_{1}}+c_{2}\;\overset{˜}{f_{2}}+c_{3}\;\overset{˜}{f_{3}}\}=\{(c_{1},c_{2},c_{3},\sum_{\left(i)\in \{(0),(1)\right\}}g_{i}\;x^{i})|\;(c_{1},c_{2},c_{3},g_{\left(i\right)})\in V_{\left(i\right)}\}$$

can be read off: {(1,−1/2,0,−k/2),(0,0,1,0),(0,0,0,1)}. Since V(x,f,Q(k)[x]) is a Q-subspace of $\overset{˜}{V}$, we plug in $\left(c_{1},c_{2},c_{3},g)=d_{1}(1,-1/2,0,-\frac{k}{2})+d_{2}(0,0,1,0)+d_{3}(0,0,0,x)+d_{4}(0,0,0,1\right)$ with unknowns $d_{1},d_{2},d_{3},d_{4}\in Q$ into (44). Together with our given $f_{i}$ and *u* we get the linear constraint $\frac{1}{2}(d_{1}-2d_{3}+2d_{4})+x(-d_{3}-d_{4})=0$ or equivalently the linear constraints $-d_{3}-d_{4}=0$ and $\frac{1}{2}(d_{1}-2d_{3}+2d_{4})$. This yields $d_{3}=\frac{d_{1}}{4}$ and $d_{4}=-\frac{d_{1}}{4}$. Thus we obtain the generic solution $d_{1}(1,-\frac{1}{2},0,-\frac{k}{2}+\frac{x}{4}-\frac{1}{4})+d_{2}(0,0,1,0)$ of V(x,f,Q(k)[x]), i.e., the basis $\left\{(1,-\frac{1}{2},0,-\frac{k}{2}+\frac{x}{4}-\frac{1}{4}),(0,0,1,0)\right\}$ of V(x,f,Q(k)[x]).

Remark 7.14 (1) In the underlying algorithm of Proposition 7.12 we construct for all i∈S the solution spaces given in (57) and combine them in one stroke as proposed in (58). This approach is interesting if one wants to perform calculations in parallel. Another approach is to apply similar tactics as given in Subsection 7.1: compute a basis of one of the (57), plug in the found solutions and continue with an updated Ansatz in terms of the remaining monomials. In this way, one usually shortens step by step the length of the vectors ${\overset{˜}{f}}_{i}$ in (57) and ends up very soon at a trivial situation (shortcut).

(2) A different approach is to consider an *R*-extension (F[x],σ) of (F,σ) of order *d* as a holonomic expression (Zeilberger, 1990; Chyzak, 2000; Koutschan, 2013) over a difference field. Then as worked out in Schneider (2005b), Eröcal (2011), a solution $g=\sum_{i=0}^{d-1}g_{i}\;x^{i}$ and $c_{i}\in \mathrm{const}\;F$ of (9) leads to a coupled system of first-order difference equations in terms of the $g_{i}$ that can be uncoupled explicitly. More precisely, there is an explicitly given formula that constitutes a higher-order parameterized linear difference equation in $g_{d-1}$ and the parameters $c_{i}$. Solving this difference equation in terms of $g_{d-1}$ and the $c_{i}$ delivers automatically the remaining coefficients $g_{i}$, i.e., the solution *g* of (9). Here one usually has to solve a general higher-order linear difference equation. For further details on the holonomic Ansatz in the context of algebraic ring extensions (also on handling such objects in the basis of idempotent elements, van der Put and Singer, 1997; Hardouin and Singer, 2008) we refer to Eröcal (2011). The advantage of the reduction technique proposed in Proposition 7.12 is that it can be applied in one stroke for nested *R*-extensions. In particular, Problem PFLDE can be always reduced again to Problem PFLDE by possibly switching to $\left(F,\sigma^{k}\right)$ for some k>1. In this way, general higher-order linear difference equations can be avoided.

Combining all algorithmic parts of this article we obtain the following result.

Theorem 7.15 *Let* (E,σ) *with* $E=F\langle t_{1}\rangle \ldots \langle t_{e}\rangle$ *be a simple* $R\Pi \Sigma^{⁎}$*-extension of a constant-stable and computable field* (F,σ)*. Suppose that for all R-monomials the periods are positive, and the orders and periods of the R-monomials are given explicitly. Then Problem PFLDE in* (E,σ) *for* ${\left(F^{⁎}\right)}_{F}^{E}$ *is solvable if one of the following holds.*

(1) *All* $t_{i}$ *are* $R\Sigma^{⁎}$*-monomials and PFLDE is solvable in* $\left(F,\sigma^{k}\right)$ *for* $F^{⁎}$ *for all* k>0*.*
(2) *Problem PMT is solvable in* (F,σ) *for* $F^{⁎}$ *and Problem PFLDE is solvable in* $\left(F,\sigma^{k}\right)$ *for* $F^{⁎}$ *for all* k>0*.*

Proof Let $H={\left(F^{⁎}\right)}_{F}^{E}$. Recall that by Theorem 2.24 we have that ${\mathrm{sconst}}_{H}\;E\setminus \{0\}\leq E^{⁎}$, i.e., Problem PFLDE is applicable in (E,σ) for *H*. By Lemma 4.10 we can reorder the generators of the $R\Pi \Sigma^{⁎}$-extension such that $\left(\overset{¯}{E},\sigma \right)$ is an $F^{⁎}$-simple *R*-extension of (F,σ) and (E,σ) is a *G*-simple $\Pi \Sigma^{⁎}$-extension of $\left(\overset{¯}{E},\sigma \right)$ with $G={\left(F^{⁎}\right)}_{F}^{\overset{¯}{E}}$. Note that the multiplicative group $F^{⁎}$ is closed under *σ*, ${\mathrm{sconst}}_{\left(F^{⁎}\right)}(F,\sigma^{l})=F^{⁎}$ for all l>0 and ${\mathrm{sconst}}_{\left(F^{⁎}\right)}\;\overset{¯}{E}\setminus \{0\}\leq {\overset{¯}{E}}^{⁎}$ by Corollary 4.3. Thus we can apply Proposition 7.12. Hence Problem PFLDE is solvable in $\left(\overset{¯}{E},\sigma \right)$ for *G*. If we are in case (1), i.e., no Π-monomials occur, we can apply iteratively Theorem 7.1 and obtain an algorithm to solve Problem PFLDE in (E,σ) for $G_{\overset{¯}{E}}^{E}=H$. If we are in case (2), i.e., Π-monomials may occur, we exploit in addition our assumptions together with Theorem 2.26.(2). This shows that we can solve Problem PMT in (E,σ) for *H* (and in any sub-difference ring by truncating the tower of extensions). Again the iterative application of Theorem 7.1 shows that Problem PFLDE is solvable in (E,σ) for $G_{\overset{¯}{E}}^{E}=H$.  □

Proof 7.16 **Theorem 2.26.(3)**

Let (E,σ) be a simple $R\Pi \Sigma^{⁎}$-extension of (F,σ) where (F,σ) is computable and strong constant-stable. Then by Corollary 5.6 (parts 3 and 4) the periods and orders of all *R*-monomials are positive and can be computed. Thus Theorem 7.15.(2) is applicable which completes the proof. □

We remark that in Theorem 2.26.(3) one can drop the condition that Problem PMT is solvable in (F,σ) for $F^{⁎}$ if in the $R\Pi \Sigma^{⁎}$-extension no Π-monomials occur, i.e., one applies part one and not part two of Theorem 7.15.

## 8 Conclusion

We provided important building blocks that extend the well established difference field theory to a difference ring theory. In this setting one can handle in addition objects such as (4). We elaborated algorithms for the (multiplicative) telescoping problem (Problems T and MT) and the (multiplicative) parameterized telescoping problem (Problems PT and PMT). In particular, Problem PT enables one to apply Zeilberger's creative telescoping paradigm in the rather general class of simple $R\Pi \Sigma^{⁎}$-extensions. In order to derive these algorithms we showed that certain semi-constants (resp. semi-invariants) of the difference rings under consideration form a multiplicative group.

Currently, the underlying engine of Theorem 2.23 with the ground field machinery of Subsection 2.3.3 is fully implemented within the summation package^12^ Sigma. In this way one can treat big classes of indefinite nested sums and products involving algebraic objects like ${\left(-1\right)}^{k}$. In particular, one can treat d'Alembertian solutions of linear recurrences as worked out in Subsection 2.4. We emphasize that these algorithms are enhanced by the refinements described in Schneider (2004c, 2005c, 2007b, 2008, 2015), Abramov and Petkovšek (2010) in order to find sum representations with certain optimality criteria, like optimal nesting depth.

The machinery to handle nested *R*-extensions (see Theorem 2.26) is not incorporated in Sigma yet. First, further investigations will be necessary so that the new algorithms can be merged with the difference field enhancements of Sigma.

Another challenging task is to push forward the difference ring theory and the underlying algorithms in order to relax the requirements in Theorems 2.23 and 2.26 that the $R\Pi \Sigma^{⁎}$-extensions are simple and/or that the ground difference ring is strong constant-stable. In this regard, we refer to the comments given in Example 2.20.

In any case, the currently developed toolbox widens the class of indefinite nested sums and products in the setting of difference rings. We are looking forward to see new kinds of applications that can be attacked with this machinery.

## Appendix A A short index

M(f,A), 91

V(u,f,A), 91

A〈t〉, 88

$A{\langle t\rangle }_{a,b}$, 97

deg, 97

$\left(A,\sigma )\leq (\overset{˜}{A},\overset{˜}{\sigma }\right)$, 86

$G_{G}^{A}$, 93

ford(f), 107

sconst(A,σ), sconstA, 91

〈S〉, 85

ldeg, 97

ord(f), 87

per(f), 107

${\mathrm{sconst}}_{G}(A,\sigma )$, ${\mathrm{sconst}}_{G}\;A$, 91

$f_{\left(k,\sigma \right)}$, $f_{\left(k\right)}$, 97

$\Pi \Sigma^{⁎}$-field, 88

constant field/ring, 85

difference

 ring/field, 85

 ring/field extension, 85

extension

 (nested) Π, $\Sigma^{⁎}$, *R*, *R*Π, $R\Sigma^{⁎}$, $\Pi \Sigma^{⁎}$,

 $R\Pi \Sigma^{⁎}$, 88

 Π, 86

 *R*, 88

 $\Sigma^{⁎}$, 86

 algebraic, 87

 simple, *G*-simple, 93

 single-rooted, 93

 unimonomial, 86

function

 degree, 97

 factorial order, 107

 order, 87

 period, 107

 rising factorial, 97

monomial

 Π, $\Sigma^{⁎}$, *R*, *R*Π, $R\Sigma^{⁎}$, $\Pi \Sigma^{⁎}$, $R\Pi \Sigma^{⁎}$, 88

 simple, *G*-simple, 93

Problem

 FPLDE, 92

 MT, 89

 O, 89

 PMT, 91

 PT, 85

 T, 85

product group, 93

ring

 (strong) constant-stable, 94

 connected, 97

 constant-stable, 94

 reduced, 97

semi-constant, 91

## Footnotes

1 We restrict Karr's Σ-field extensions to $\Sigma^{⁎}$-field extensions being slightly less general but covering all sums treated explicitly in Karr's work (Karr, 1981).

2 Note: Theorem 2.12.(3) is a shortcut for “part 3 of Theorem 2.12”. The same convention will be applied for other references.

3 Note that this restriction, in particular the choice of *G*, is fundamental: it is the essential step to specify the type of products that one can handle algorithmically; see Definition 2.19.

4 Note: If λ:=ord(α)>0, we have that λ∈M, i.e., the rank of *M* is 1. In particular, we can construct an *R*-extension (A,σ)≤(A[t],σ) with σ(t)=αt iff λ=m>0.

5 Note: If G is a field, any single-rooted $R\Pi \Sigma^{⁎}$-extension is simple by Corollary 4.15.

6 Instead of Problem O it suffices to know the orders of all the *R*-monomials in (G,σ)≤(E,σ).

7 We emphasize that we will always work with the automorphism *σ* during the reduction process. Only in the base cases we might face the problem to solve instances of Problem PFLDE in $\left(F,\sigma^{k}\right)$ with k>1. In a nutshell, we succeed in avoiding to work with $\sigma^{k}$ for some k>1 as much as possible. This strategy is of particular advantage, if (F,σ) is built only by few summation objects. Then the typical phenomenon of the expression swell in symbolic summation due to $\sigma^{k}$ is prevented as much as possible.

8 In principal, we could also take one primitive *r*th root of unity *α*. However, if α∉K, we have to extend the constant field. By efficiency reasons we prefer to stay in the original field. We remark that extending the constant field would not produce further relations.

9 By arguments as in the proof of Lemma 2.16 it follows dim⁡(L)≤1.

10 Note that we reduced the problem to find a polynomial solution of (49) with maximal degree 1 to a polynomial solution of (49) with maximal degree 0. This degree reduction has been achieved by introducing an extra parameter $c_{2}$. In general, the more $\Sigma^{⁎}$-monomials are involved, the more parameters will be introduced within the proposed degree reduction.

11 By a mild modification of the proof it suffices to take a *λ* such that $\alpha_{\left(\lambda \right)}\in \mathrm{const}\;A$ holds.

12 The Sigma package can be downloaded from www.risc.jku.at/research/combinat/software/Sigma/.

## References

[br0030] Ablinger J., Blümlein J., Schneider C. (2011). Harmonic sums and polylogarithms generated by cyclotomic polynomials. J. Math. Phys..

[br0040] Ablinger J., Blümlein J., Schneider C. (2013). Analytic and algorithmic aspects of generalized harmonic sums and polylogarithms. J. Math. Phys..

[br0010] Ablinger J., Behring A., Blümlein J., De Freitas A., Hasselhuhn A., von Manteuffel A., Round M., Schneider C., Wissbrock F. (2014). The 3-loop non-singlet heavy flavor contributions and anomalous dimensions for the structure function $F_{2}(x,Q^{2})$ and transversity. Nucl. Phys. B.

[br0020] Ablinger J., Blümlein J., De Freitas A., Hasselhuhn A., von Manteuffel A., Round M., Schneider C., Wissbrock F. (2014). The transition matrix element $A_{gq}(N)$ of the variable flavor number scheme at $O(\alpha_{s}^{3})$. Nucl. Phys. B.

[br0050] Abramov S.A. (1971). On the summation of rational functions. Zh. Vychisl. Mat. Mat. Fiz..

[br0060] Abramov, S.A., Bronstein, M., Petkovšek, M., Schneider, C., in preparation.

[br0070] Abramov S.A., Petkovšek M., von zur Gathen J. (1994). D'Alembertian solutions of linear differential and difference equations. Proc. ISSAC'94.

[br0080] Abramov S.A., Petkovšek M. (2010). Polynomial ring automorphisms, rational (w,σ)-canonical forms, and the assignment problem. J. Symb. Comput..

[br0090] Bauer A., Petkovšek M. (1999). Multibasic and mixed hypergeometric Gosper-type algorithms. J. Symb. Comput..

[br0100] Becker Thomas, Weispfenning Volker (1993). Gröbner Bases: A Computational Approach to Commutative Algebra.

[br0120] Blümlein J., Klein S., Schneider C., Stan F. (2012). A symbolic summation approach to Feynman integral calculus. J. Symb. Comput..

[br0110] Blümlein J., Hasselhuhn A., Klein S., Schneider C. (2013). The $O(\alpha_{s}^{3}n_{f}T_{F}^{2}C_{A,F})$ contributions to the gluonic massive operator matrix elements. Nucl. Phys. B.

[br0130] Bronstein M. (1997). Symbolic Integration I, Transcendental Functions.

[br0140] Bronstein M. (2000). On solutions of linear ordinary difference equations in their coefficient field. J. Symb. Comput..

[br0150] Chyzak F. (2000). An extension of Zeilberger's fast algorithm to general holonomic functions. Discrete Math..

[br0160] Cohn R.M. (1965). Difference Algebra.

[br0170] Eröcal B. (February 2011). Algebraic extensions for summation in finite terms.

[br0180] Gosper R.W. (1978). Decision procedures for indefinite hypergeometric summation. Proc. Natl. Acad. Sci. USA.

[br0190] Hardouin C., Singer M.F. (2008). Differential Galois theory of linear difference equations. Math. Ann..

[br0200] Hendriks P.A., Singer M.F. (1999). Solving difference equations in finite terms. J. Symb. Comput..

[br0220] Horn P., Koepf W., Sprenger T. (2012). *m*-fold hypergeometric solutions of linear recurrence equations revisited. Math. Comput. Sci..

[br0230] Karpilovsky G. (1983). On finite generation of unit groups of commutative group rings. Arch. Math. (Basel).

[br0240] Karr M. (1981). Summation in finite terms. J. ACM.

[br0250] Karr M. (1985). Theory of summation in finite terms. J. Symb. Comput..

[br0260] Kauers M., Paule P. (2011). The Concrete Tetrahedron: Symbolic Sums, Recurrence Equations, Generating Functions, Asymptotic Estimates.

[br0270] Kauers M., Schneider C., Dumas J.G. (2006). Application of unspecified sequences in symbolic summation. Proc. ISSAC'06.

[br0280] Kauers M., Schneider C. (2006). Indefinite summation with unspecified summands. Discrete Math..

[br0290] Kauers M., Schneider C., Brown C.W. (2007). Symbolic summation with radical expressions. Proc. ISSAC'07.

[br0300] Koutschan C., Schneider C., Blümlein J. (2013). Creative telescoping for holonomic functions. Computer Algebra in Quantum Field Theory: Integration, Summation and Special Functions.

[br0310] Levin A. (2008). Difference Algebra.

[br0320] Neher Erhard (2009). Invertible and nilpotent elements in the group algebra of a unique product group. Acta Appl. Math..

[br0330] Osburn R., Schneider C. (2009). Gaussian hypergeometric series and extensions of supercongruences. Math. Comput..

[br0340] Paule P. (1995). Greatest factorial factorization and symbolic summation. J. Symb. Comput..

[br0350] Paule P., Riese A., Ismail M., Rahman M. (1997). A mathematica *q*-analogue of Zeilberger's algorithm based on an algebraically motivated approach to *q*-hypergeometric telescoping. Special Functions, *q*-Series and Related Topics.

[br0360] Petkovšek M. (1992). Hypergeometric solutions of linear recurrences with polynomial coefficients. J. Symb. Comput..

[br0380] Petkovšek M., Zakrajšek H., Schneider C., Blümlein J. (2013). Solving linear recurrence equations with polynomial coefficients. Computer Algebra in Quantum Field Theory: Integration, Summation and Special Functions.

[br0370] Petkovšek M., Wilf H.S., Zeilberger D. (1996). A=B.

[br0390] Prodinger H., Schneider C., Wagner S. (2011). Unfair permutations. Eur. J. Comb..

[br0400] Risch R. (1969). The problem of integration in finite terms. Trans. Am. Math. Soc..

[br0410] Schneider C. (2000). An implementation of Karr's summation algorithm in mathematica. Sémin. Lothar. Comb.

[br0420] Schneider C. (November 2001). Symbolic summation in difference fields.

[br0430] Schneider C. (2004). A collection of denominator bounds to solve parameterized linear difference equations in ΠΣ-extensions. An. Univ. Timiş., Ser. Mat.-Inform..

[br0440] Schneider C. (2004). The summation package Sigma: underlying principles and a rhombus tiling application. Discrete Math. Theor. Comput. Sci..

[br0450] Schneider C., Gutierrez J. (2004). Symbolic summation with single-nested sum extensions. Proc. ISSAC'04.

[br0460] Schneider C. (2005). Degree bounds to find polynomial solutions of parameterized linear difference equations in ΠΣ-fields. Appl. Algebra Eng. Commun. Comput..

[br0470] Schneider C. (2005). A new Sigma approach to multi-summation. Adv. Appl. Math..

[br0480] Schneider C. (2005). Product representations in ΠΣ-fields. Ann. Comb..

[br0490] Schneider C. (2005). Solving parameterized linear difference equations in terms of indefinite nested sums and products. J. Differ. Equ. Appl..

[br0500] Schneider C. (2007). Apéry's double sum is plain sailing indeed. Electron. J. Comb..

[br0510] Schneider C. (2007). Simplifying sums in ΠΣ-extensions. J. Algebra Appl..

[br0520] Schneider C. (2007). Symbolic summation assists combinatorics. Sémin. Lothar. Comb.

[br0530] Schneider C. (2008). A refined difference field theory for symbolic summation. J. Symb. Comput..

[br0540] Schneider C. (2010). Parameterized telescoping proves algebraic independence of sums. Ann. Comb..

[br0550] Schneider C. (2010). Structural theorems for symbolic summation. Appl. Algebra Eng. Commun. Comput..

[br0560] Schneider C., Carey A., Ellwood D., Paycha S., Rosenberg S. (2010). A symbolic summation approach to find optimal nested sum representations. Motives, Quantum Field Theory, and Pseudodifferential Operators.

[br0570] Schneider C., Schneider C., Blümlein J. (2013). Simplifying multiple sums in difference fields. Computer Algebra in Quantum Field Theory: Integration, Summation and Special Functions.

[br0580] Schneider C. (2014). Modern summation methods for loop integrals in quantum field theory: the packages Sigma, EvaluateMultiSums and SumProduction. J. Phys. Conf. Ser..

[br0590] Schneider C., Weimann M., Guitierrez J., Schicho J. (2015). Fast algorithms for refined parameterized telescoping in difference fields. Computer Algebra and Polynomials, Applications of Algebra and Number Theory.

[br0600] van der Put M., Singer M.F. (1997). Galois Theory of Difference Equations.

[br0210] van Hoeij M. (1999). Finite singularities and hypergeometric solutions of linear recurrence equations. J. Pure Appl. Algebra.

[br0610] Zeilberger D. (1990). A holonomic systems approach to special functions identities. J. Comput. Appl. Math..

[br0620] Zeilberger D. (1991). The method of creative telescoping. J. Symb. Comput..

